# Microwave-assisted synthesis of 4-oxo-2-butenoic acids by aldol-condensation of glyoxylic acid†

**DOI:** 10.1039/d1ra05539a

**Published:** 2021-10-05

**Authors:** Mélanie Uguen, Conghao Gai, Lukas J. Sprenger, Hang Liu, Andrew G. Leach, Michael J. Waring

**Affiliations:** Cancer Research UK Drug Discovery Unit, Newcastle University Centre for Cancer, Chemistry, School of Natural and Environmental Sciences, Newcastle University Bedson Building Newcastle upon Tyne NE1 7RU UK mike.waring@ncl.ac.uk; Organic Chemistry Group, College of Pharmacy, Naval Medical University Shanghai 200433 P. R. China; Division of Pharmacy and Optometry, School of Health Sciences, University of Manchester Manchester M13 9PT UK

## Abstract

4-Oxobutenoic acids are useful as biologically active species and as versatile intermediates for further derivatisation. Currently, routes to their synthesis can be problematic and lack generality. Reaction conditions for the synthesis of 4-oxo-2-butenoic acid by microwave-assisted aldol-condensation between methyl ketone derivatives and glyoxylic acid have been developed. They provide the desired products in moderate to excellent yields for a wide range of substrates, by applying a simple procedure to accessible starting materials. The investigation revealed different conditions are required depending on the nature of the methylketone substituent, with aryl derivatives proceeding best using tosic acid and aliphatic substrates reacting best with pyrrolidine and acetic acid. This substituent effect is rationalised by frontier orbital calculations. Overall, this work provides methods for synthesis of 4-oxo-butenoic acids across a broad range of substrates.

## Abstract 2

A new method for the synthesis of 4-oxo-2-butenoic acids is described by aldol condensation with methyl ketones. Substrate dependent conditions are rationalised mechanistically with quantum mechanically derived molecular orbital energies.

## Graphical abstract

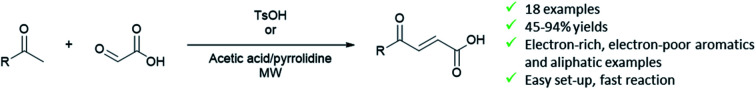

## Introduction

**Fig. 1:**
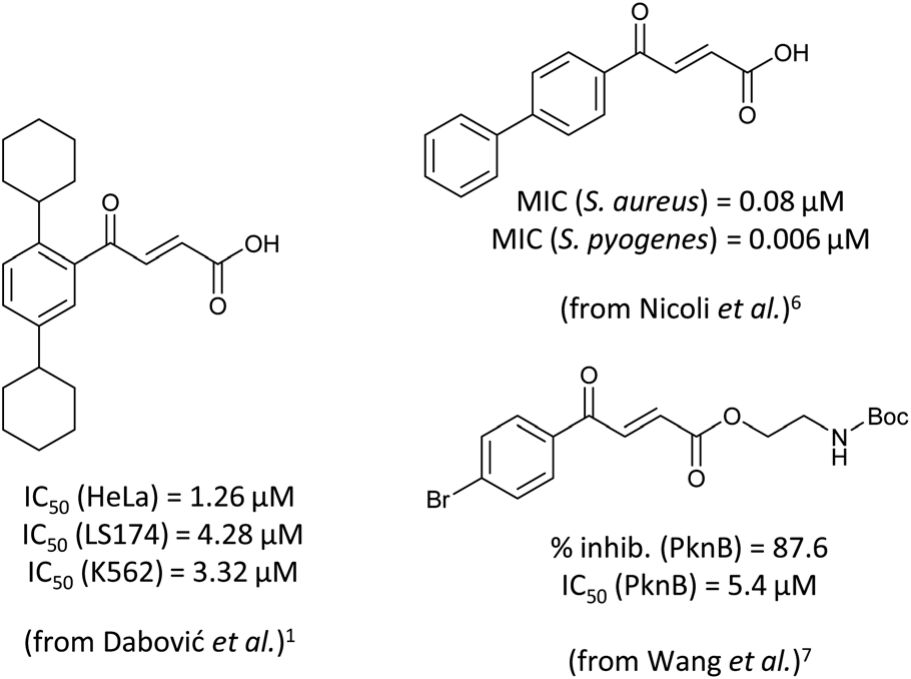
Examples of biologically active 4-oxo-2-butenoic acids and analogues.

4-Oxo-2-butenoic acids are interesting building blocks for drug discovery. Several derivatives have shown biological activity in their own right, for example to treat cancer,^1,2^ neurodegenerative,^3^ metabolic,^4^ and gastric^5^ conditions as well as antimicrobial^6,7^ or antifungal^6^ properties (Fig. 1). In addition, their high reactivity makes them versatile intermediates for further derivatisation.

**Fig. 2:**
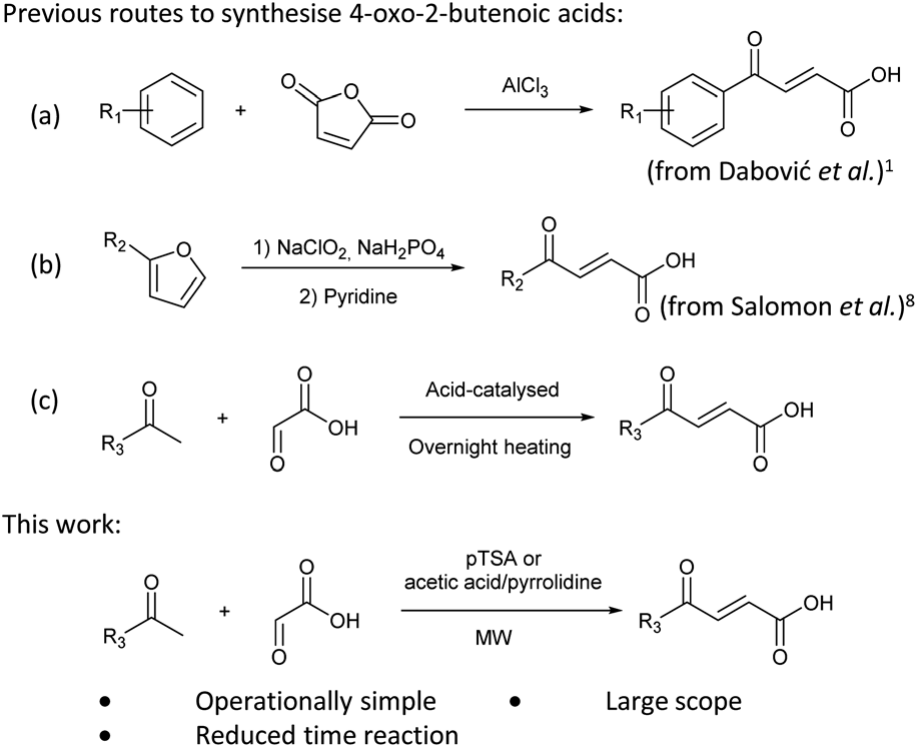
Synthetic routes for the preparation of 4-oxo-2-butenoic acid derivatives.

Preparation of 4-oxo-2-butenoic acids has often proven to be scope-limited, with Friedel–Crafts acylations^1^ used for aromatic substrates (Fig. 2) and oxidative furan-opening^8^ for aliphatic ones (Fig. 2). Although we managed to obtain the desired 4-oxo-2-butenoic acid product when applying the oxidative furan oxidative conditions to an electron-rich aromatic example, we were unable to identify conditions compatible with electron-deficient aromatic substrates.

Aldol-condensation with glyoxylic acid is compatible with a larger range of starting materials with a few examples available in the literature, mainly using acetophenone derivatives as substrates (Fig. 2). Literature reported conditions are typically acid-promoted, most commonly by acetic acid,^7,9–12^ sulphuric acid,^13–17^ phosphoric acid,^13,18^ toluene-4-sulfonic acid^19^ and formic acid,^20^ with two acids frequently used together.^12,19,21–28^ The acid promotors are usually used neat or in large excess under reflux for relatively long periods of time. A few base-promoted procedures are also described in the literature, using potassium carbonate^29,30^ or sodium hydroxide at reflux or under reduced pressure.^31^ All these conditions are quite harsh on the reactants and resulting products, thus limiting the scope of the transformation.

Therefore, we decided to investigate the aldol-condensation with glyoxylic acid to identify efficient conditions for the preparation of 4-oxo-2-butenoic acid derivatives. We aimed to develop operationally simple conditions compatible with a large range of substrates by using microwave heating, which had the potential to reduce reaction times and increase yields.

## Results and discussion

Initially, 4-methoxyacetophenone was used as a test substrate. Reactions with glyoxylic acid under basic conditions were first attempted but showed moderate conversion to the desired product (1) (Table S1†). Reaction *via* the pyrrolidine-derived enamine also showed moderate conversion, with the initial aldol adduct obtained in larger amount than the desired aldol-condensation product. Attempts to force the water elimination by addition of tosyl chloride led to an improved conversion of the starting material. It was proposed that the tosyl chloride was hydrolysed *in situ* and the reaction was catalysed by tosic acid. Accordingly, carrying out the direct tosic acid-promoted aldol-condensation provided the desired product in good yield (70%), confirming the previous hypothesis (Table 1, entry 1). These conditions were applied to the more electron deficient 4-cyanoacetophenone, in which case, even after prolonged heating, some starting ketone remained (Table 1, entry 2). To improve the conversion towards the formation of the product 2 as well as reducing the heating time, microwave-assisted heating was performed using similar conditions, leading to a moderate isolated yield of 2 (32%, Table 1, entry 3). Optimisation of the irradiation time and temperature allowed an increase of the yield to 45% with a reduced reaction time of 1 h (Table 1, entry 4). Shorter reaction time led to incomplete conversion of the starting material whereas increase of the temperature from 160 °C to 180 °C led to partial or total degradation of the desired product (Table 1, entries 5–7), suggesting that heating at 160 °C for 1 hour were giving the best results. These findings suggest that increased temperature and pressure allowed by the microwave reactor are key to drive the reaction while allowing a simple reaction set-up. Therefore, these optimised conditions were also applied to 4-methoxyacetophenone and showed an improved yield of 94% (Table 2).

**Table 1:** Reaction conditions attempted for aldol-condensation with glyoxylic acid

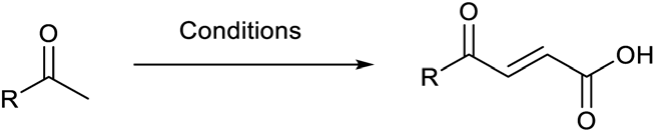						
Entry	R	Cond.^a^	Heat.	Time	Temp. (°C)	Isol. yield
1	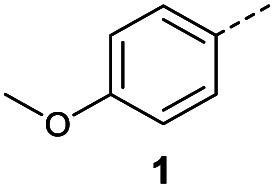	A	Conv.	48 h	80	70%
2	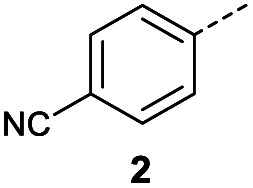	A	Conv.	72 h	80	n.c.^b^
3		A	MW	16 h	100	32%
4		A	MW	1 h	160	45%
5		A	MW	10 min	180	<20%^b^
6		A	MW	15 min	180	40%
7		A	MW	5 min	160	0%
8	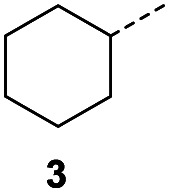	A	MW	16 h	100	0%
9		B	MW	8 h	80	25%
10		B	MW	10 min	100	14%
11		B	MW	8 h	60	52%

a Conditions A: 3.0 eq. glyoxylic acid monohydrate, 1.0 eq. TsOH monohydrate, dioxane; conditions B: 3.0 eq. glyoxylic acid monohydrate, 1.0 eq. pyrrolidine, 1.0 eq. acetic acid, MeOH.

b Products not isolated.

**Table 2:** Scope evaluation of the aldol-condensation reaction with glyoxylic acid promoted by tosic acid^a^

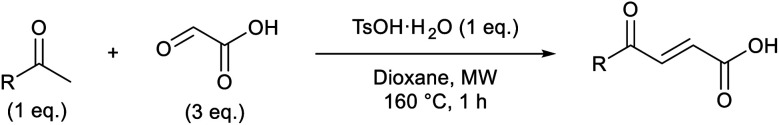					
R	Product	Yield	R	Product	Yield
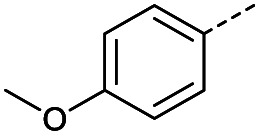	1	94%	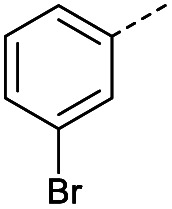	11	78%
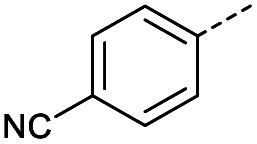	2	45%	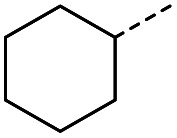	3	0%
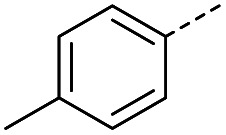	4	66%	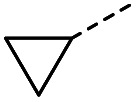	12	0%
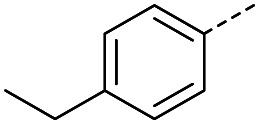	5	61%	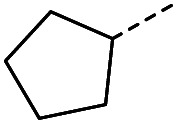	13	0%
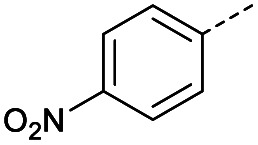	6	93%	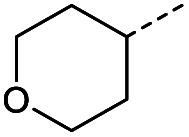	14	0%
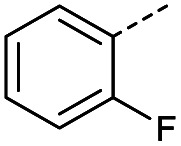	7	76%	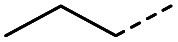	15	30%^b^^,^^c^
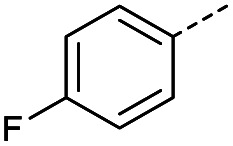	8	62%	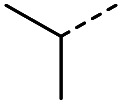	16	0%
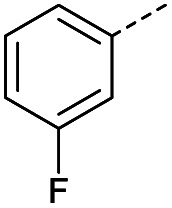	9	52%	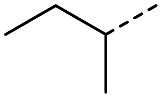	17	0%
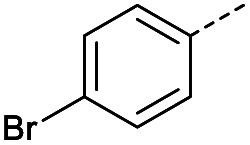	10	55%	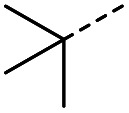	18	0%

a Typical scale: 2.4 mmol, 6 mL of solvent.

b MW temperature and time: 100 °C, 16 h.

c NMR yield.

When applying these conditions to cyclohexylmethyl ketone starting material, no desired product 3 was formed (Table 1, entry 8). The major product was the aldol adduct intermediate. However, treatment of cyclohexylmethyl ketone with glyoxylic acid, in the presence of pyrrolidine and acetic acid (Table 1, entry 9), using microwave-assisted heating, enabled the isolation of the desired product 3 in 25% yield. Increasing the temperature from 80 °C to 100 °C led to product degradation, even with decreased reaction time (Table 1, entry 10), but decreasing the temperature to 60 °C improved the yield to 52% (Table 1, entry 11).

Hence, two sets of microwave-assisted conditions for the formation of 4-oxo-2-butenoic acid by aldol-condensation of glyoxylic acid with acetyl derivative have been developed. A scope evaluation of these conditions was performed on electron-rich aromatic (1), electron-poor aromatic (2, 6–9), halogen-containing electron-neutral aromatic (10 and 11), aliphatic ring (3, 12–13), heteroatom-containing (14) and aliphatic chain (15–18) 4-oxo-2-butenoic acid derivatives (Tables 2 and 3).

TsOH-promoted aldol-condensation provided the desired products in good to excellent yields for aromatic substrates generating products 1, 2 and 4–11 in 45–94% yield (Table 2). Electron donating and withdrawing substituents and *ortho*, *meta* and *para* substitution patterns were well tolerated. No desired product formation was observed when these conditions were applied to aliphatic substrates 3, 12–14 and 16–18. Pentan-2-one, however, yielded the desired product 15 under the TsOH-promoted conditions (by NMR) as a minor component relative to the expected internal aldol-condensation product 19 (ratio 1 : 1.5, Fig. S1†). After heating at 100 °C for 16 hours, 19 was the only product formed (99% yield). Hence, this suggests, as expected, that the tosic acid mediated reaction may not be compatible with methyl ketones bearing an additional enolisable centre.

The pyrrolidine-acetic acid conditions were also applied to the selected substrates (Table 3). With aliphatic substrates, these conditions yielded the desired products 3 and 12–17 with yields between 43% and 92%, in which no product was obtained with the TsOH-promoted conditions. In this case, substrates with additional enolisable centres were tolerated (3, 12–17) and the internal aldol adduct was not observed. No desired product was obtained for the *t*-butyl ketone 18, presumably due to increased steric hindrance. Aromatic products 1, 4, 5 and 7–11 were obtained in poor 4 to 12% yields, much lower than with the TsOH-promoted conditions. However, no product was observed for the electron-poor examples 2 and 6 nor for the aliphatic chain 15 (Table 3).

**Table 3:** Scope evaluation of the aldol-condensation reaction with glyoxylic acid promoted by acetic acid and pyrrolidine^a^

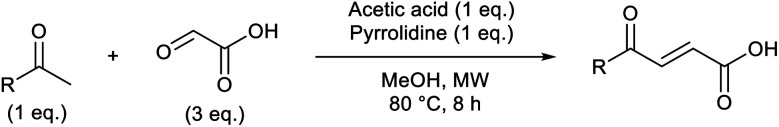					
R	Product	Yield	R	Product	Yield
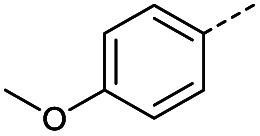	1	12%	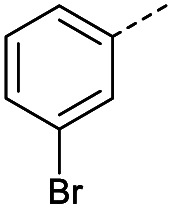	11	<4%
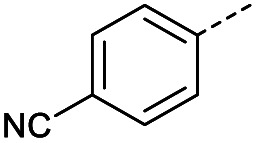	2	0%	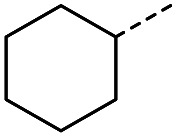	3	52%
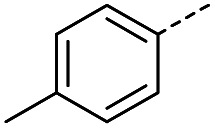	4	5%	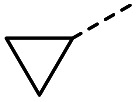	12	63%
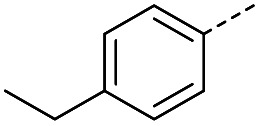	5	5%	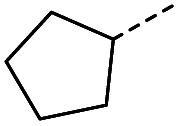	13	52%
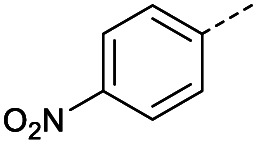	6	0%	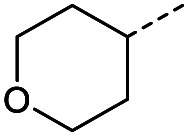	14	43%
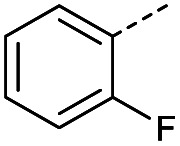	7	5%	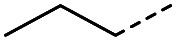	15	0%
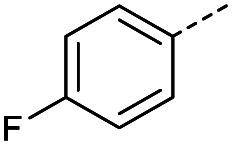	8	5%	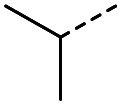	16	71%
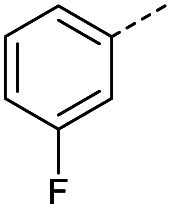	9	4%	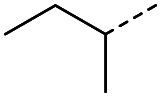	17	92%
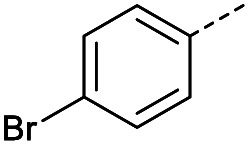	10	4%	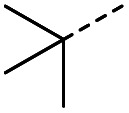	18	0%

a Typical scale: 2.4 mmol, 6 mL of solvent.

For all the examples above, the obtained 4-oxo-2-butenoic acids all had the *E* conformation for the alkene bond confirmed by NMR. No trace of the *Z* alkene was observed, demonstrating the stereoselectivity of this transformation.

Finally, scale-up of the synthesis of 2 from 0.69 mmol to 6.9 mmol demonstrated the scalability of the TsOH-promoted reaction with no change in yield. Scale up of the synthesis of 3 using the pyrrolidine-acetic acid conditions from 4.0 mmol to 7.2 mmol gave increased yield (from 38% to 52%).

**Scheme 1:**
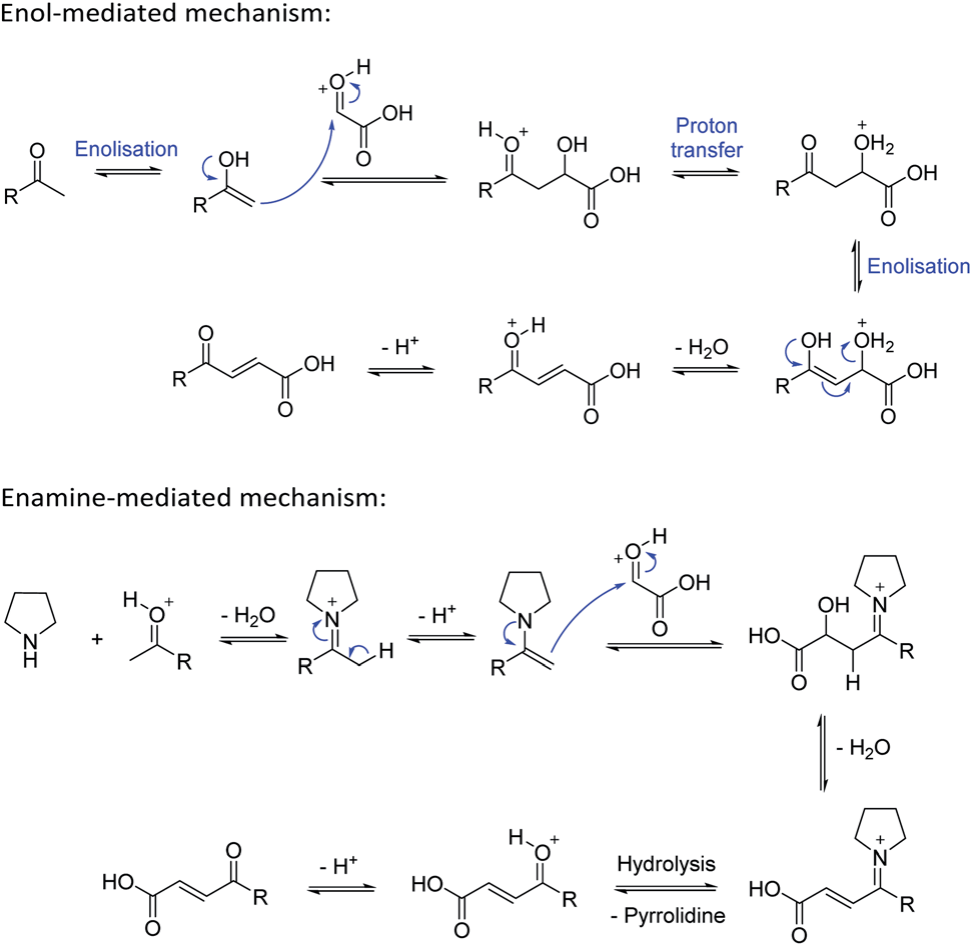
Mechanisms for the aldol condensation reaction.

**Fig. 3:**
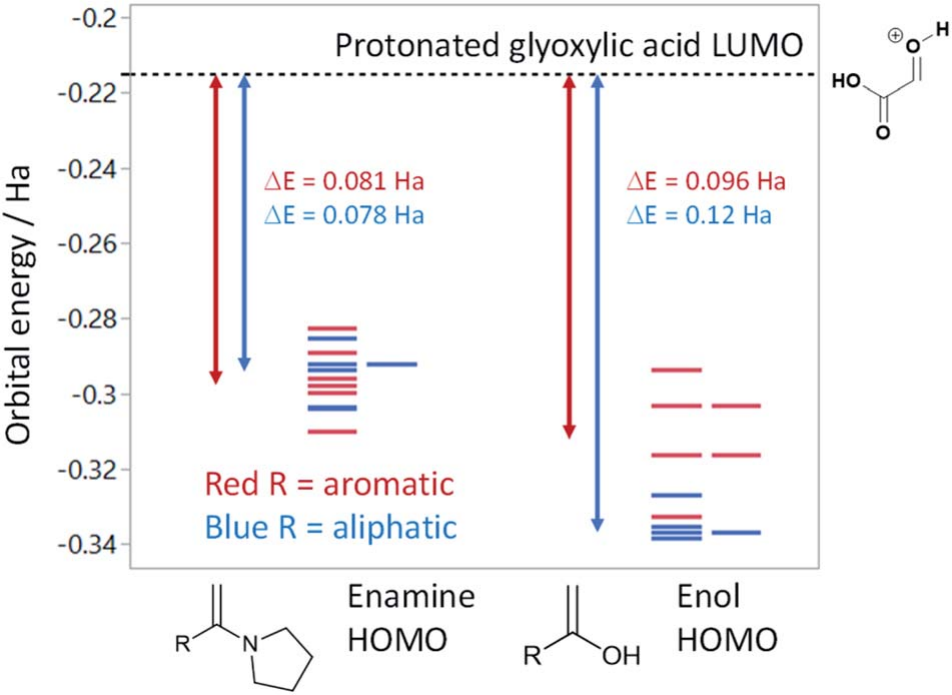
Calculated HOMO energies at the RHF/6-31+G** level for selected enamines and enols relative to the protonated glyoxylic acid LUMO. Double-headed arrows show the average HOMO–LUMO gap for aromatic (red) and aliphatic (blue) substituents.

Based on the assumption that the reaction proceeds by attack of the protonated glyoxylic acid by the enol form of the methylketone under the tosic acid promoted conditions, and by either the enol or the enamine in the presence of pyrrolidine/acetic acid (Scheme 1) as the rate determining step, the observed differences in reactivity were rationalised from the calculated energy gaps between the protonated glyoxylic acid LUMO and the enol or enamine HOMOs (Fig. 3). Calculations employed the RHF/6-31+G** level of theory in the Gaussian09 suite of programs.^32^ Geometries were optimised and frequencies computed to verify that they are minima.

HOMOs of the enol form of the aromatic ketones are consistently higher in energy than those of the aliphatic examples. This explains why the TsOH conditions work better on aromatic compounds as the HOMO–LUMO energy gap is significantly reduced (Δ*E* (aromatic) = 0.096 Ha, Δ*E* (aliphatic) = 0.12 Ha, ΔΔ*E* = 0.024 Ha). For the aliphatic examples, HOMOs of the enamines are higher in energy compared to their corresponding enols (average HOMO–LUMO Δ*E* = 0.078 Ha). This provides an explanation as to why the pyrrolidine-acetic acid conditions work better for aliphatic substrates, and that these reactions likely proceed *via* the enamine as the predominant pathway.

## Conclusions

We successfully identified two sets of conditions that provide *E*-4-oxo-2-butenoic acids using microwave-assisted aldol-condensation with glyoxylic acid. These provide the desired products in good to excellent yields for substrate with various electronic and steric properties (electron-rich, -neutral and -poor aromatic, *ortho*-, *meta*-, and *para*-substituted aromatic, linear and cyclic aliphatic). The reaction can be scaled-up to mmol scale with no effect on the yield. It was observed that tosic acid promoted conditions work better for aromatic substrates whereas acid acetic and pyrrolidine are preferred for aliphatic substrates. These findings can be rationalised by the relative differences in HOMO–LUMO energy gaps for the enol and enamine intermediates. Overall, we describe a convenient and efficient synthesis for 4-oxo-2-butenoic acids which should allow an easier access to these biologically-relevant molecules and their derivatives.

## Experimental

### General procedure A for the synthesis of 1, 2 and 4–11

In a Biotage microwave vial, acetyl derivative (1 eq.), glyoxylic acid monohydrate (3 eq.) and TsOH monohydrate (1 eq.) were dissolved in dioxane (2.5 mL mmol^−1^). The vial was closed with a 20 mm aluminium crimp cap with a PTFE/silicone septum and heated in the microwave for 1 h at 160 °C using the low absorption mode while stirred at 600 rpm with a PTFE stirring bar. 2 M HCl aqueous solution was added to the mixture. This was extracted 3 times with CH_2_Cl_2_. Combined organic phases were dried over MgSO_4_. The solvent was removed under vacuum. A typical scale was 2.4 mmol but the reaction was successfully scaled up and down.

#### (*E*)-4-(4-Methoxyphenyl)-4-oxobut-2-enoic acid (1)

Compound 1 was obtained following General procedure A. Flash chromatography (0 to 10% 0.1 AcOH in MeOH in CH_2_Cl_2_) provided 1 as a bright yellow solid (2.59 g, 94%). *R*_f_ = 0.65 (80% EtOAc in PE); mp = 180–182 °C (from MeOH); UV *λ*_max_ (EtOH/nm) 287.2, 223.6, 200.0; FTIR (cm^−1^) *ν*_max_ 2840br (O–H acid), 1699s (C=O acid), 1661s (C=O ketone), 1592s (C=C alkene), 1511s (C=C aromatic), 1420s (O–H acid), 1167 (s, C–O methoxy); ^1^H NMR (500 MHz; CDCl_3_; Me_4_Si) *δ*_H_ 3.90 (3H, s, CH_3_), 6.88 (1H, d, *J* = 15.5 Hz, C*H*=CH), 6.97–7.02 (2H, m, CH-Ar), 7.97–8.05 (3H, m, CH=C*H* and CH-Ar); ^13^C NMR (126 MHz; CDCl_3_; Me_4_Si) *δ* 55.77 (CH_3_), 114.39 (C-Ar), 129.68 (C-Ar), 130.61 (C=C), 131.55 (C-Ar), 138.77 (C=C), 164.57 (C-Ar), 169.84 (COOH), 187.48 (CO); MS(ES+) *m*/*z* 207.2; HRMS calcd for C_11_H_10_O_4_ [M + H]^+^ 207.0579, found 207.0555.
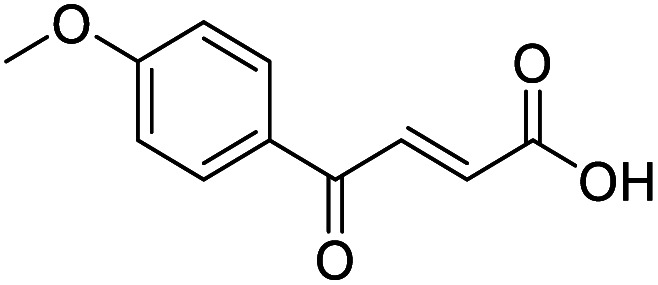


#### (*E*)-4-(4-Cyanophenyl)-4-oxobut-2-enoic acid (2)

Compound 2 was obtained following General procedure A. Normal phase flash chromatography (0 to 10% 0.1% AcOH in MeOH in CH_2_Cl_2_) yielded compounds 2 as a pale yellow solid (443 mg, 32%). *R*_f_ = 0.12 (10% MeOH in CH_2_Cl_2_); mp = 134–140 °C (from MeOH); UV *λ*_max_ (EtOH/nm) 256.4; FTIR (cm^−1^) *ν* 3063br (O–H acid), 2231w (CN); ^1^H NMR (500 MHz; MeOD; Me_4_Si) *δ*_H_ 6.83 (1H, d, *J* = 15.6 Hz, C*H*=CH), 7.91 (1H, d, *J* = 15.6 Hz, CH=C*H*), 7.93 (2H, d, *J* = 8.4 Hz, CH-Ar), 8.17 (2H, d, *J* = 8.4 Hz, CH-Ar); ^13^C NMR (methanol-*d*_4_, 126 MHz) *δ*_C_ 117.86 (CN), 118.87 (C-Ar), 130.43 (C-Ar), 133.93 (C-Ar), 135.05 (C=C), 136.84 (C=C), 141.19 (C-Ar), 168.23 (COOH), 190.30 (CO); MS (ES+) *m*/*z* = 201.1 [M–H]^−^; HRMS calcd for C_11_H_7_NO_3_ 200.0348 [M + H]^+^ found 200.0363.
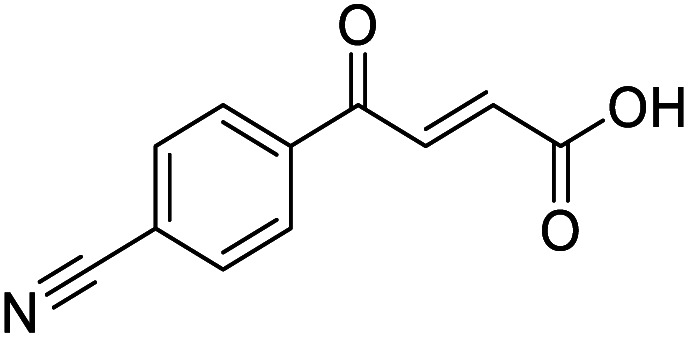


#### (*E*)-4-Oxo-4-(*p*-tolyl)but-2-enoic acid (4)

Compound 4 was obtained following General procedure A. Flash chromatography (0 to 50% EtOAc in petroleum ether) yielded 4 as a yellow solid (300 mg, 66%). *R*_f_ = 0.39 (10% MeOH in CH_2_Cl_2_); mp = 134–136 °C (from MeOH); UV *λ*_max_ (EtOH/nm) 280.0, 231.1; FTIR (cm^−1^) *ν*_max_ 3031br (OH acid), 2923s (CH), 1664s (C=O acid), 1410s (O–H acid); ^1^H NMR (600 MHz; DMSO-*d*_6_; Me_4_Si) *δ*_H_ 2.40 (3H, s, CH_3_), 6.67 (1H, d, *J* = 15.6 Hz, C*H*=CH), 7.38 (2H, d, *J* = 7.8 Hz, CH-Ar), 7.87 (1H, d, *J* = 15.6 Hz, CH=C*H*), 7.94 (2H, d, *J* = 7.8 Hz, CH-Ar), 13.14 (1H, br s, COOH); ^13^C NMR (150 MHz; DMSO-*d*_6_; Me_4_Si) *δ*_C_ 21.67 (CH_3_), 129.34 (C-Ar), 130.04 (C-Ar), 133.09 (C=C), 134.18 (C-Ar), 136.65 (C=C), 145.10 (C-Ar), 166.78 (COOH), 189.30 (CO); MS(ES^+^) *m*/*z* 191.1 [M + H]^+^; HRMS calcd for C_11_H_11_O_3_ [M + H]^+^ 191.0629, found 191.0697.
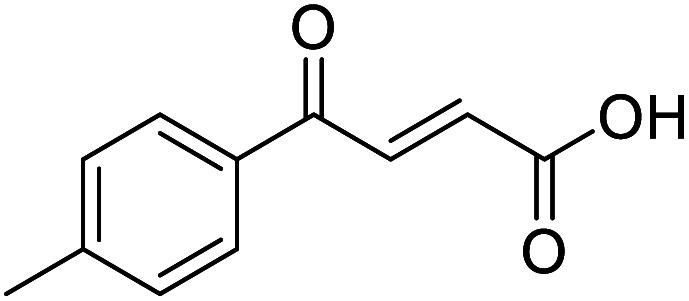


#### (*E*)-4-(4-Ethylphenyl)-4-oxobut-2-enoic acid (5)

Compound 5 was obtained following General procedure A. Flash chromatography (0 to 50% EtOAc in petroleum ether) yielded 5 as a yellow solid (300 mg, 61%). *R*_f_ = 0.42 (10% MeOH in CH_2_Cl_2_); mp = 91–93 °C (from MeOH); UV *λ*_max_ (EtOH/nm) 282.0, 232.1; FTIR (cm^−1^) *ν*_max_ 3052br (OH acid), 2968s (CH), 2934s (CH), 1666s (C=O acid), 1415s (O–H acid); ^1^H NMR (600 MHz; DMSO-*d*_6_; Me_4_Si) *δ*_H_ 1.20 (3H, t, *J* = 7.2 Hz, CH_3_), 2.70 (2H, q, *J* = 7.2 Hz, CH_2_), 6.68 (1H, d, *J* = 15.6 Hz, C*H*=CH), 7.41 (2H, d, *J* = 7.8 Hz, CH-Ar), 7.87 (1H, d, *J* = 15.6 Hz, CH=C*H*), 7.96 (2H, d, *J* = 7.8 Hz, CH-Ar), 13.15 (1H, br s, COOH); ^13^C NMR (150 MHz; DMSO-*d*_6_; Me_4_Si) *δ*_C_ 15.48 (CH_3_), 28.68 (CH_2_), 128.87 (C-Ar), 129.45 (C-Ar), 133.18 (C=C), 134.43 (C-Ar), 136.62 (C=C), 151.07 (C-Ar), 166.80 (COOH), 189.34 (CO); MS(ES^+^) *m*/*z* 205.1 [M + H]^+^; HRMS calcd for C_12_H_13_O_3_ [M + H]^+^ 205.0791, found 205.0865.
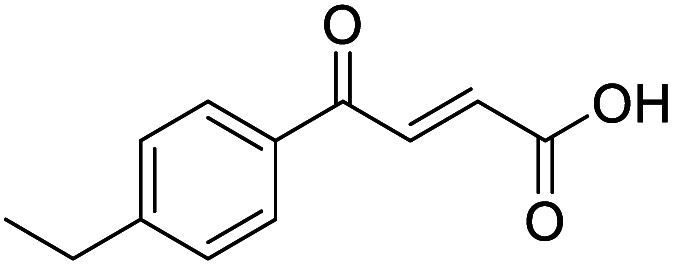


#### (*E*)-4-(4-Nitrophenyl)-4-oxobut-2-enoic acid (6)

Compound 6 was obtained following General procedure A. Flash chromatography (0 to 50% EtOAc in petroleum ether) yielded 6 as a yellow solid (493 mg, 93%). *R*_f_ = 0.40 (10% MeOH in CH_2_Cl_2_); mp = 173–175 °C (from MeOH); UV *λ*_max_ (EtOH/nm) 269.0; FTIR (cm^−1^) *ν*_max_ 2989br (OH acid), 1604s (C=O acid), 1530s (NO_2_), 1419s (O–H acid), 1350s (NO_2_); ^1^H NMR (600 MHz; DMSO-*d*_6_; Me_4_Si) *δ*_H_ 6.71 (1H, d, *J* = 15.6 Hz, C*H*=CH), 7.87 (1H, d, *J* = 15.6 Hz, CH=C*H*), 8.25 (2H, d, *J* = 9.0 Hz, CH-Ar), 8.36 (2H, d, *J* = 9.0 Hz, CH-Ar), 13.25 (1H, br s, COOH); ^13^C NMR (150 MHz; DMSO-*d*_6_; Me_4_Si) *δ*_C_ 124.42 (C-Ar), 130.65 (C-Ar), 134.36 (C=C), 136.23 (C=C), 141.23 (C-Ar), 150.63 (C-Ar), 166.56 (COOH), 189.40 (CO); MS(ES^+^) *m*/*z* 222.1 [M + H]^+^; HRMS calcd for C_10_H_6_NO_5_ [M–H]^−^ 220.0329, found 220.0257.
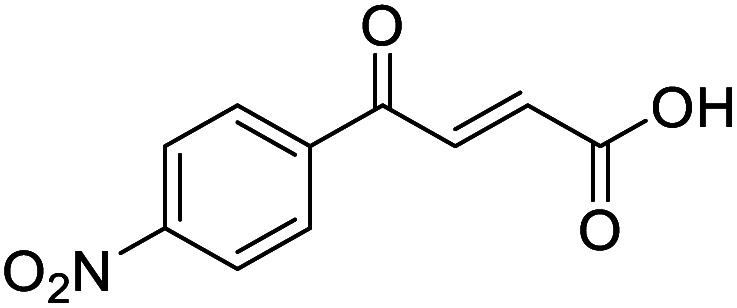


#### (*E*)-4-(2-Fluorophenyl)-4-oxobut-2-enoic acid (7)

Compound 7 was obtained following General procedure A. Flash chromatography (0 to 50% EtOAc in petroleum ether) yielded 7 as a yellow solid (352 mg, 76%). *R*_f_ = 0.41 (10% MeOH in CH_2_Cl_2_); mp = 105–106 °C (from MeOH); UV *λ*_max_ (EtOH/nm) 246.0; FTIR (cm^−1^) *ν*_max_ 3001br (OH acid), 1665s (C=O acid), 1421s (O–H acid); ^1^H NMR (600 MHz; DMSO-*d*_6_; Me_4_Si) *δ*_H_ 6.62 (1H, dd, *J* = 15.6 Hz, *J*_HF_ = 0.6 Hz, C*H*=CH), 7.37–7.41 (2H, m, CH-Ar), 7.57 (1H, dd, *J* = 15.6 Hz, *J*_HF_ = 3.0 Hz, CH=C*H*), 7.70–7.74 (1H, m, CH-Ar), 7.80–7.83 (1H, m, CH-Ar), 13.21 (1H, br s, COOH); ^13^C NMR (150 MHz; DMSO-*d*_6_; Me_4_Si) *δ*_C_ 117.30 (d, *J*_CF_ = 22.2 Hz, C-Ar), 125.50 (d, *J*_CF_ = 2.8 Hz, C-Ar), 125.54 (d, *J*_CF_ = 16.6 Hz, C-Ar), 131.22 (C=C), 133.33 (C=C), 136.06 (d, *J*_CF_ = 9.0 Hz, C-Ar), 139.14 (d, *J*_CF_ = 5.1 Hz, C-Ar), 161.40 (d, *J*_CF_ = 252.0 Hz, CF-Ar), 166.63 (COOH), 188.54 (CO); 19F NMR (282 MHz, DMSO-*d*_6_) *δ*_F_ −111.29; MS(ES^+^) *m*/*z* 195.1 [M + H]^+^; HRMS calcd for C_10_H_8_FO_3_ [M + H]^+^ 195.0379, found 195.0452.
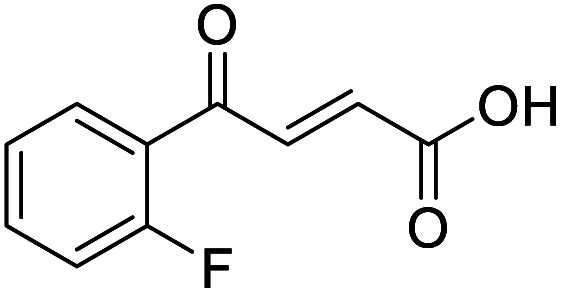


#### (*E*)-4-(4-Fluorophenyl)-4-oxobut-2-enoic acid (8)

Compound 8 was obtained following General procedure A. Flash chromatography (0 to 50% EtOAc in petroleum ether) yielded 8 as a yellow solid (289 mg, 62%). *R*_f_ = 0.41 (10% MeOH in CH_2_Cl_2_); mp = 108–109 °C (from MeOH); UV *λ*_max_ (EtOH/nm) 243.1; FTIR (cm^−1^) *ν*_max_ 2978br (OH acid), 1666s (C=O acid), 1411s (O–H acid); ^1^H NMR (600 MHz; DMSO-*d*_6_; Me_4_Si) *δ*_H_ 6.68 (1H, d, *J* = 15.6 Hz, C*H*=CH), 7.36–7.40 (2H, m, CH-Ar), 7.87 (1H, d, *J* = 15.6 Hz, CH=C*H*), 8.10–8.14 (2H, m, CH-Ar), 13.07 (1H, br s, COOH); ^13^C NMR (150 MHz; DMSO-*d*_6_; Me_4_Si) *δ*_C_ 116.51 (d, *J*_CF_ = 21.9 Hz, C-Ar), 132.33 (d, *J*_CF_ = 9.6 Hz, C-Ar), 133.37 (d, *J*_CF_ = 2.0 Hz, C-Ar), 133.49 (C=C), 136.40 (C=C), 165.90 (d, *J*_CF_ = 251.6 Hz, CF-Ar), 166.70 (COOH), 188.54 (CO); 19F NMR (282 MHz, DMSO-*d*_6_) *δ*_F_ −104.52; MS(ES^+^) *m*/*z* 195.1 [M + H]^+^; HRMS calcd for C_10_H_8_FO_3_ [M + H]^+^ 195.0379, found 195.0468.
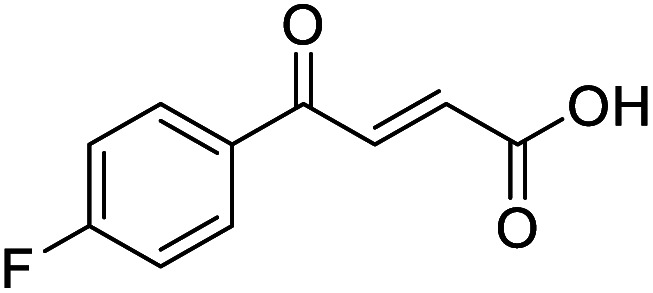


#### (*E*)-4-(3-Fluorophenyl)-4-oxobut-2-enoic acid (9)

Compound 9 was obtained following General procedure A. Flash chromatography (0 to 50% EtOAc in petroleum ether) yielded 9 as a white solid (239 mg, 52%). *R*_f_ = 0.41 (10% MeOH in CH_2_Cl_2_); mp = 109–110 °C (from MeOH); UV *λ*_max_ (EtOH/nm) 246.0; FTIR (cm^−1^) *ν*_max_ 3079br (OH acid), 1668s (C=O acid), 1416s (O–H acid); ^1^H NMR (600 MHz; DMSO-*d*_6_; Me_4_Si) *δ*_H_ 6.70 (1H, d, *J* = 15.6 Hz, C*H*=CH), 7.54–7.58 (1H, m, CH-Ar), 7.62–7.65 (1H, m, CH-Ar), 7.79–7.81 (1H, m, CH-Ar), 7.85 (1H, d, *J* = 15.6 Hz, CH=C*H*), 7.90 (1H, dd, *J* = 7.8 Hz, *J*_HF_ = 0.6 Hz, CH-Ar), 13.18 (1H, br s, COOH); ^13^C NMR (150 MHz; DMSO-*d*_6_; Me_4_Si) *δ*_C_ 115.56 (d, *J*_CF_ = 22.4 Hz, C-Ar), 121.35 (d, *J*_CF_ = 21.3 Hz, C-Ar), 125.55 (d, *J*_CF_ = 1.4 Hz, C-Ar), 131.68 (d, *J*_CF_ = 7.8 Hz, C-Ar), 133.94 (C=C), 136.23 (C=C), 138.80 (d, *J*_CF_ = 6.2 Hz, C-Ar), 162.72 (d, *J*_CF_ = 244.5 Hz, CF-Ar), 166.64 (COOH), 189.02 (CO); ^19^F-NMR (282 MHz, DMSO-*d*_6_) *δ*_F_ −111.78; MS(ES^+^) *m*/*z* 195.1 [M + H]^+^; HRMS calcd for C_10_H_6_FO_3_ [M–H]^−^ 193.0379, found 193.0306.
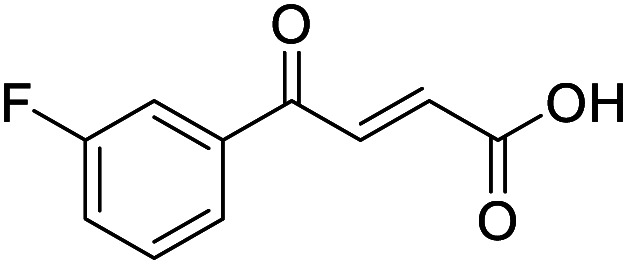


#### (*E*)-4-(4-Bromophenyl)-4-oxobut-2-enoic acid (10)

Compound 10 was obtained following General procedure A. Flash chromatography (0 to 50% EtOAc in petroleum ether) yielded 10 as a white solid (334 mg, 55%). *R*_f_ = 0.41 (10% MeOH in CH_2_Cl_2_); mp = 149–152 °C (from MeOH); UV *λ*_max_ (EtOH/nm) 265.0; FTIR (cm^−1^) *ν*_max_ 3020br (OH acid), 1666s (C=O acid), 1418s (O–H acid); ^1^H NMR (600 MHz; DMSO-*d*_6_; Me_4_Si) *δ*_H_ 6.69 (1H, d, *J* = 15.6 Hz, C*H*=CH), 7.78–7.81 (2H, m, CH-Ar), 7.85 (1H, d, *J* = 15.6 Hz, CH=C*H*), 7.96–7.98 (2H, m, CH-Ar), 13.09 (1H, br s, COOH); ^13^C NMR (150 MHz; DMSO-*d*_6_; Me_4_Si) *δ*_C_ 128.65 (CBr-Ar), 131.21 (C-Ar), 132.55 (C-Ar), 133.78 (C=C), 135.64 (C-Ar), 136.26 (C=C), 166.69 (COOH), 189.28 (CO); MS(ES^+^) *m*/*z* 254.1 [M(^79^Br) + H]^+^ and 256.1 [M(^81^Br) + H]^+^; HRMS calcd for C_10_H_8_^79^BrO_3_ [M + H]^+^ 254.9585, found 254.9652.
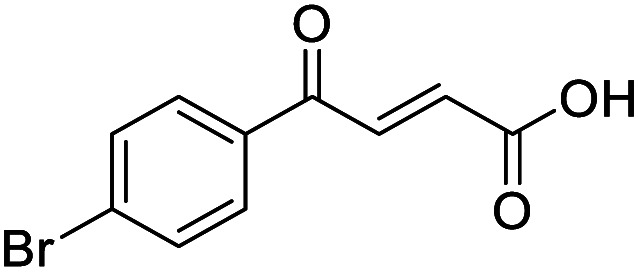


#### (*E*)-4-(3-Bromophenyl)-4-oxobut-2-enoic acid (11)

Compound 11 was obtained following General procedure A. Flash chromatography (0 to 50% EtOAc in petroleum ether) yielded 11 as a white solid (476 mg, 78%). *R*_f_ = 0.41 (10% MeOH in CH_2_Cl_2_); mp = 148–151 °C (from MeOH); UV *λ*_max_ (EtOH/nm) 244.8; FTIR (cm^−1^) *ν*_max_ 2992br (OH acid), 1672s (C=O acid), 1416s (O–H acid); ^1^H NMR (600 MHz; DMSO-*d*_6_; Me_4_Si) *δ*_H_ 6.70 (1H, d, *J* = 15.6 Hz, C*H*=CH), 7.55 (1H, t, *J* = 7.8 Hz, CH-Ar), 7.85 (1H, d, *J* = 15.6 Hz, CH=C*H*), 7.91–7.92 (1H, m, CH-Ar), 8.04–8.05 (1H, m, CH-Ar), 8.15–8.16 (1H, m, CH-Ar), 13.08 (1H, br s, COOH); ^13^C NMR (150 MHz; DMSO-*d*_6_; Me_4_Si) *δ*_C_ 122.81 (CBr-Ar), 128.32 (C-Ar), 131.57 (C-Ar), 131.68 (C-Ar), 134.03 (C=C), 136.24 (C=C), 136.95 (C-Ar), 138.66 (C-Ar), 166.66 (COOH), 189.05 (CO); MS(ES^+^) *m*/*z* 254.1 [M(^79^Br) + H]^+^ and 256.1 [M(^81^Br) + H]^+^; HRMS calcd for C_10_H_6_^79^BrO_3_ [M–H]^−^ 252.9585, found 252.9503.
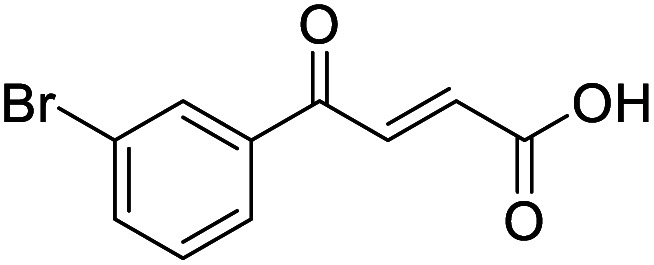


### General procedure B for the synthesis of 3, 12–14 and 16–17

In a Biotage microwave vial, methyl ketone-containing compound (1 eq.), glyoxylic acid monohydrate (3 eq.) and acetic acid (1 eq.) were dissolved in MeOH (2.5 mL mmol^−1^) then pyrrolidine (1 eq.) was added. The vial was sealed with a 20 mm aluminium crimp cap with PTFE/silicon septa and the mixture stirred for 5 min using a PTFE stirring bar. The mixture was irradiated using the MW at 60 °C for 8 h while stirred at 600 rpm. The solvent was removed under vacuum. A typical scale was 2.4 mmol but the reaction was successfully scaled up and down.

#### (*E*)-4-Cyclohexyl-4-oxobut-2-enoic acid (3)

Compound 3 was obtained following General procedure B. Flash chromatography (0 to 15% 0.1% AcOH in MeOH in CH_2_Cl_2_) yielded 3 as a beige solid (277 mg, 38%). *R*_f_ = 0.32 (5% MeOH in CH_2_Cl_2_); mp = 113–115 °C (from MeOH); UV *λ*_max_ (EtOH/nm) 330.8, 219.8; FTIR (cm^−1^) *ν*_max_ 3062br (OH acid), 2922s (CH), 2851s (CH), 1660s (C=O acid), 1427s (O–H acid); 1H NMR (500 MHz; MeOD; Me_4_Si) *δ*_H_ 1.05–1.21 (5H, m, Cy), 1.51–1.67 (5H, m, Cy), 2.50 (1H, tt, *J* = 10.7, 3.4 Hz, Cy), 6.42 (1H, d, *J* = 15.9 Hz, C=C), 6.91 (1H, d, *J* = 15.9 Hz, C=C); ^13^C NMR (126 MHz; MeOD; Me_4_Si) *δ*_C_ 26.49 (Cyc), 29.31 (Cyc), 50.35 (Cyc), 132.21 (C=C), 139.56 (C=C), 168.50 (COOH), 204.38 (CO); MS(ES+) *m*/*z* 183.1 [M + H]^+^; HRMS calcd for C_10_H_14_O_3_ [M–H]^−^ 181.0870, found 181.0870.
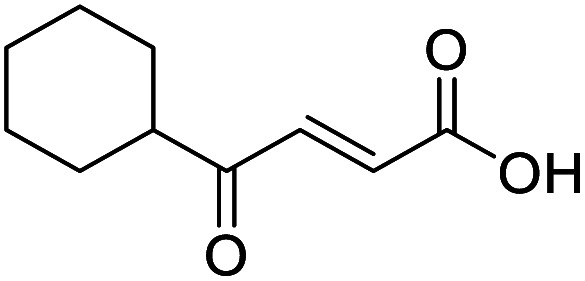


#### (*E*)-4-Cyclopropyl-4-oxobut-2-enoic acid (12)

Compound 12 was obtained following General procedure B. Flash chromatography (0 to 50% EtOAc in petroleum ether) yielded 12 as a yellow oil (213 mg, 63%). *R*_f_ = 0.40 (10% MeOH in CH_2_Cl_2_); UV *λ*_max_ (EtOH/nm) 223.0; FTIR (cm^−1^) *ν*_max_ 3012br (OH acid), 1715s (C=O acid), 1392s (O–H acid); ^1^H NMR (600 MHz; DMSO-*d*_6_; Me_4_Si) *δ*_H_ 0.96–0.98 (2H, m, C*H*H-cyclopropane), 0.99–1.03 (2H, m, CH*H*-cyclopropane), 2.47–2.51 (1H, m, CH-cyclopropane), 6.70 (1H, d, *J* = 16.0 Hz, C*H*=CH), 7.03 (1H, d, *J* = 16.0 Hz, CH=C*H*), 13.06 (1H, br s, COOH); ^13^C NMR (150 MHz; DMSO-*d*_6_; Me_4_Si) *δ*_C_ 12.07 (CH_2_), 19.82 (CH), 131.92 (C=C), 139.56 (C=C), 167.00 (COOH), 200.20 (CO); MS(ES^+^) *m*/*z* 141.1 [M + H]^+^; HRMS calcd for C_7_H_8_O_3_ [M–H]^−^ 139.0473, found 139.0400.
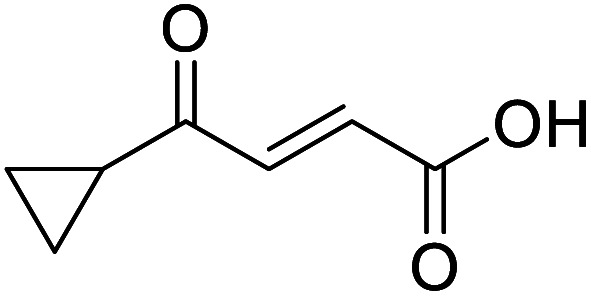


#### (*E*)-4-Cyclopentyl-4-oxobut-2-enoic acid (13)

Compound 13 was obtained following General procedure B. Flash chromatography (0 to 50% EtOAc in petroleum ether) yielded 13 as a yellow oil (210 mg, 52%). *R*_f_ = 0.38 (10% MeOH in CH_2_Cl_2_); UV *λ*_max_ (EtOH/nm) 221.0; FTIR (cm^−1^) *ν*_max_ 3065br (OH acid), 2951s (CH), 2869s (CH), 1687s (C=O acid), 1428s (O–H acid); ^1^H NMR (600 MHz; DMSO-*d*_6_; Me_4_Si) *δ*_H_ 1.54–1.58 (4H, m, CH_2_C*H*_2_-cyclopentane), 1.64–1.85 (4H, m, C*H*_2_CH_2_-cyclopentane), 3.31 (1H, m, CH-cyclopenpane), 6.62 (1H, d, *J* = 15.6 Hz, C*H*=CH), 6.99 (1H, d, *J* = 15.6 Hz, CH=C*H*), 13.09 (1H, br s, COOH); ^13^C NMR (150 MHz; DMSO-*d*_6_; Me_4_Si) *δ*_C_ 26.09 (CH_2_*C*H_3_-cyclopentane), 28.63 (*C*H_2_CH_3_-cyclopentane), 49.34 (CH), 132.00 (C=C), 139.26 (C=C), 166.97 (COOH), 202.25 (CO); MS(ES^+^) *m*/*z* 169.1 [M + H]^+^; HRMS calcd for C_9_H_12_O_3_ [M–H]^−^ 167.0798, found 167.0725.
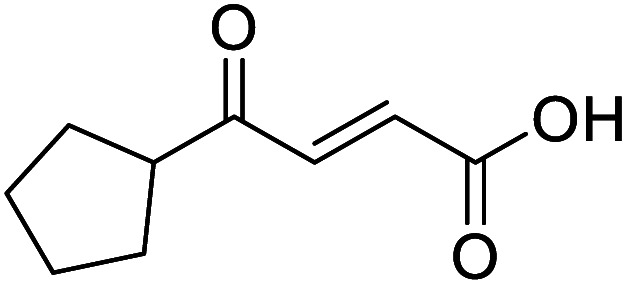


#### (*E*)-4-Oxo-4-(tetrahydro-2*H*-pyran-4-yl)but-2-enoic acid (14)

Compound 14 was obtained following General procedure B. Flash chromatography (0 to 10% 0.1% AcOH in MeOH in CH_2_Cl_2_) yielded 14 as an orange solid (321 mg, 38%). *R*_f_ = 0.41 (5% MeOH in CH_2_Cl_2_); mp = 90–92 °C (from MeOH); UV *λ*_max_ (EtOH/nm) 243.0, 201.7; FTIR (cm^−1^) *ν*_max_ 3067br (O–H acid), 2963 (CH), 2841 (CH), 1669s (C=O acid), 1424s (O–H acid), 1117s (C–O ether); ^1^H NMR (500 MHz; MeOD; Me_4_Si) *δ*_H_ 1.53 (2H, dtd, *J* = 13.4, 11.4, 4.2 Hz, Pyr), 1.71 (2H, ddd, *J* = 13.4, 4.2, 2.1 Hz, Pyr), 2.93 (1H, tt, *J* = 11.4, 3.8 Hz, Pyr), 3.42 (2H, td, *J* = 11.4, 2.1 Hz, Pyr), 3.87 (2H, ddd, *J* = 11.4, 4.2, 2.1 Hz, Pyr), 6.62 (1H, d, *J* = 15.9 Hz, C=C), 7.07 (1H, d, *J* = 15.9 Hz, C=C); ^13^C NMR (126 MHz; MeOD; Me_4_Si) *δ*_C_ 29.06 (Pyr), 47.01 (Pyr), 68.07 (Pyr), 132.75 (C=C), 139.07 (C=C), 168.44 (COOH), 202.59 (CO); MS(ES+) *m*/*z* 185.1 [M + H]^+^; HRMS calcd for C_9_H_12_O_4_ [M + H]^+^ 183.0663, found 183.0644.
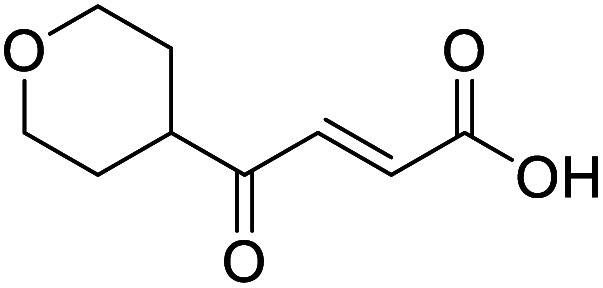


#### (*E*)-5-Methyl-4-oxohex-2-enoic acid (16)

Compound 16 was obtained following General procedure B. Normal phase flash chromatography (0 to 10% 0.1% AcOH in MeOH in CH_2_Cl_2_) yielded compound 16 as a pale yellow oil (53 mg, 37%). *R*_f_ = 0.57 (5% MeOH in CH_2_Cl_2_); UV *λ*_max_ (EtOH/nm) 221.8; FTIR (cm^−1^) *ν*_max_ 3376br (O–H acid), 1686s (C=O acid), 1638s (C=O ketone), 1466 (O–H acid); ^1^H NMR (500 MHz; MeOD; Me_4_Si) *δ*_H_ 0.99 (6H, d, *J* = 6.8 Hz, Me), 2.83 (1H, hept, *J* = 6.8 Hz, CH), 6.54 (1H, d, *J* = 15.9 Hz, C=C), 7.01 (1H, d, *J* = 15.9 Hz, C=C); ^13^C NMR (126 MHz; MeOD; Me_4_Si) *δ* 18.24 (Me), 40.57 (CMe_2_), 132.44 (C=C), 139.36 (C=C), 168.56 (COOH), 205.15 (CO); MS(ES+) *m*/*z* 143.1 [M + H]^+^; HRMS calcd for C_7_H_10_O_3_ [M–H]^−^ 141.0557, found 141.0536.
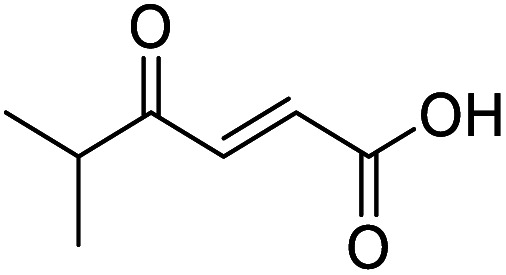


#### (*E*)-5-Methyl-4-oxohept-2-enoic acid (17)

Compound 17 was obtained following General procedure B. Flash chromatography (0 to 50% EtOAc in petroleum ether) yielded 17 as a yellow oil (343 mg, 92%). *R*_f_ = 0.39 (10% MeOH in CH_2_Cl_2_); UV *λ*_max_ (EtOH/nm) 272.4; FTIR (cm^−1^) *ν*_max_ 2968br (OH acid), 1631s (C=O acid), 1454s (O–H acid); ^1^H NMR (600 MHz; DMSO-*d*_6_; Me_4_Si) *δ*_H_ 0.82 (3H, t, *J* = 7.2 Hz, CH_2_C*H*_3_), 1.02 (3H, d, *J* = 6.6 Hz, CH_3_), 1.37 (1H, dq, *J* = 6.6 Hz and 7.2 Hz, C*H*H), 1.37 (1H, dq, *J* = 6.6 Hz and 7.2 Hz, CH*H*), 2.89 (1H, sext, *J* = 6.6 Hz, CH), 6.62 (1H, d, *J* = 15.6 Hz, C*H*=CH), 7.05 (1H, d, *J* = 15.6 Hz, CH=C*H*), 13.10 (1H, br s, COOH); ^13^C NMR (150 MHz; DMSO-*d*_6_; Me_4_Si) *δ*_C_ 11.61 (CH_2_*C*H_3_), 15.59 (CH_3_), 25.53 (*C*H_2_CH_3_), 45.52 (CH), 131.93 (C=C), 138.73 (C=C), 166.92 (COOH), 203.66 (CO); MS(ES^+^) *m*/*z* 157.1 [M + H]^+^; HRMS calcd for C_8_H_12_O_3_ [M–H]^−^ 155.0786, found 155.0713.
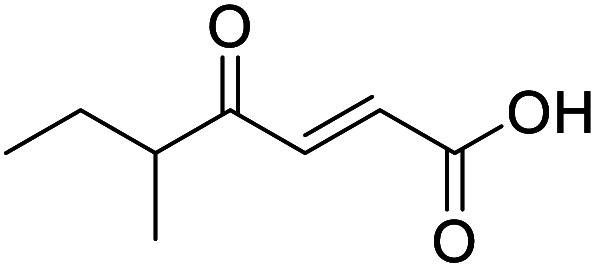


#### (*E*)-3-Ethyl-4-oxopent-2-enoic acid (19)

Compound 19 was obtained following General procedure A. Normal phase flash chromatography (0 to 10% 0.1% AcOH in MeOH in CH_2_Cl_2_) yielded compound 19 as a yellow oil (132 mg, 0.93 mmol, 99%). *R*_f_ = 0.63 (5% MeOH in CH_2_Cl_2_); UV *λ*_max_ (EtOH/nm) 220.9; FTIR (cm^−1^) *ν* 3144br (O–H acid), 2974 (CH), 1681s (C=O acid), 1362 (O–H acid); ^1^H NMR (500 MHz; MeOD; Me_4_Si) *δ*_H_ 0.99 (3H, t, *J* = 7.5 Hz, Me), 2.37 (3H, s, COMe), 2.71 (2H, q, *J* = 7.5 Hz, CH_2_), 6.64 (1H, s, C=C); ^13^C NMR (126 MHz; MeOD; Me_4_Si) *δ*_C_ 14.06 (Me), 20.87 (CH_2_), 26.56 (COMe), 128.17 (C=C), 156.25 (C=C), 169.07 (COOH), 201.87 (CO); MS(ES+) *m*/*z* 143.1 [M + H]^+^; HRMS calcd for C_7_H_10_O_3_ [M–H]^−^ 141.0557, found 141.0560.
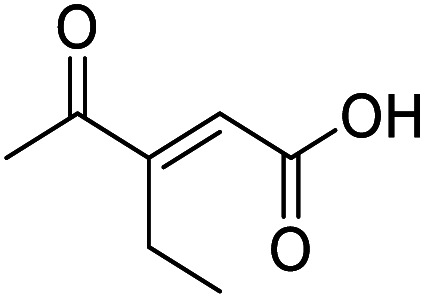


## Author contributions

The investigation was performed by M. U., C. G., L. J. S. and H. L. under the supervision of M. J. W. A. G. L. carried out the molecular orbital calculations. The manuscript was written by M. U. and reviewed by M. J. W.

## Conflicts of interest

There are no conflicts to declare.

## Supplementary Material

RA-011-D1RA05539A-s001
