# Supplementary material for: Microwave-assisted synthesis of 4-oxo-2-butenoic acids by aldol-condensation of glyoxylic acid
Source: RSC Adv. 2021 Oct 5;11(48):30229–36. doi: 10.1039/d1ra05539a (PMC9041125; doi:10.1039/d1ra05539a)
Supplement: RA-011-D1RA05539A-s001 [file RA-011-D1RA05539A-s001.pdf]

Electronic Supplementary Material (ESI) for RSC Advances.
This journal is © The Royal Society of Chemistry 2021

Microwave-assisted synthesis of 4-oxo-2-butenoic acids by aldol-condensation of glyoxylic acid

Mélanie Uguen, [†a]* Conghao Gai,[†b] Lukas J. Sprenger[a], Hang Liu[b] Andrew G. Leach[c] and Michael J. Waring[a]*

[a]Cancer Research UK Drug Discovery Unit, Newcastle University Centre for Cancer, Chemistry, School of Natural and Environmental Sciences, Newcastle University, Bedson Building, Newcastle upon Tyne NE1 7RU, U.K; [b]Organic Chemistry Group, College of Pharmacy, Naval Medical University, Shanghai, P. R. China; [c]Division of Pharmacy and Optometry, School of Health Sciences, University of Manchester, Manchester M13 9PT, U.K. orcid.org/0000-0003-1325-8273; †Co-authors.

Supplementary information

Content

1. Table S1. Optimisation of the transformation of 4-methoxyacetophenone into 1 via aldol-condensation. 2

2. Figure S1. Crude NMR of TsOH-promoted aldol-condensation on pentan-2-one. A mixture of desired product 15 and side-product 19 1:1.5 was obtained. 3

3. Table S2. Calculated HOMO and LUMO energies for different methyl ketone substrates and glyoxylic acid. 4

4. General Information for the Synthesis 5

5. Analytical Equipment 5

6. NMR spectra 6

1. Table S1. Optimisation of the transformation of 4-methoxyacetophenone into 1 via aldol-condensation.

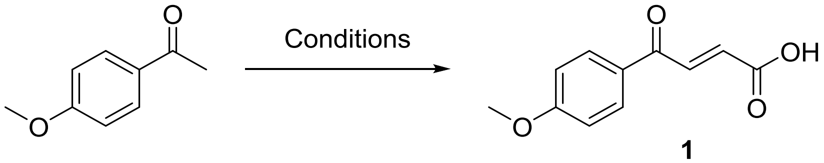

1

Entry	Conditions	Yield after purification
1	1.5 eq. glyoxylic acid monohydrate, 1.7 eq. NaH 60% dispersion in mineral oil, DMSO, 80 °C, 18 h	Low conversion[1]
2	1.5 eq. glyoxylic acid monohydrate, 1.7 eq. NaH 60% dispersion in mineral oil, DMSO, 80 °C, 18h, then 1.5 eq. NaH 60% dispersion in mineral oil, 1.5 eq. TsCl, 80 °C, 5 min	Low conversion[1]
3	1.5 eq. glyoxylic acid monohydrate 1.7 eq. LiOH monohydrate MeOH, 80°C, 18h	Moderate conversion[2]
4	3.0 eq. glyoxylic acid monohydrate, 1.0 eq. pyrrolidine, 1.0 eq. acetic acid, MeOH, MW, 80°C, 8 h	Moderate conversion[2]
5	3.0 eq. glyoxylic acid monohydrate 3.0 eq. TsCl, dioxane, 80°C, 18 h	Moderate conversion[2]
6	3.0 eq. glyoxylic acid monohydrate 3.0 eq. Tf_2O, dioxane, 80°C, 18 h	No product
7	3.0 eq. glyoxylic acid monohydrate 1.0 eq. TsOH monohydrate dioxane, 80°C, 48 h	70%

[1]Low conversion: < 10% desired product by UV and/or ELSD LC-MS analysis. [2]Moderate conversion: < 50% desired product by UV and/or ELSD LC-MS analysis.

2. Figure S1. Crude NMR of TsOH-promoted aldol-condensation on pentan-2-one. A mixture of desired product 15 and side-product 19 1:1.5 was obtained.

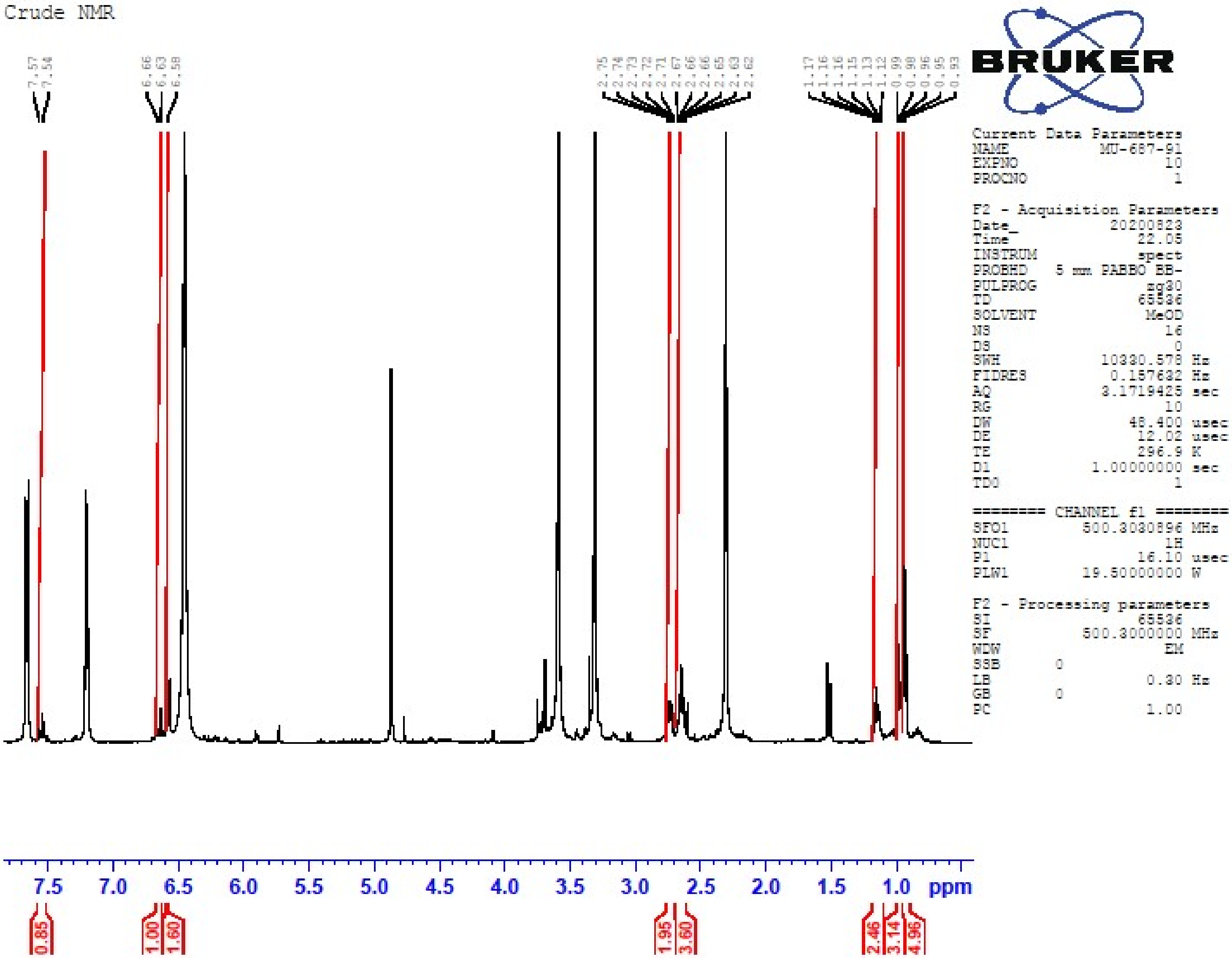

3. Table S2. Calculated HOMO and LUMO energies for different methyl ketone substrates and glyoxylic acid.

Calculations employed the RHF/6-31+G** level of theory in the Gaussian09 suite of programs. Geometries were optimised and frequencies computed to verify that they are minima.

Methyl ketone substituent	Substituent class	ENOL HOMO	ENOL LUMO	ENOLATE HOMO	ENOLATE LUMO	ENAMINE_HOMO	ENAMINE_LUMO
cyclohexyl	aliphatic	-0.33597	0.07163	-0.08481	0.16325	-0.29239	0.06944
cyclopropyl	aliphatic	-0.32685	0.06935	-0.08144	0.18209	-0.2852	0.07097
isopropyl	aliphatic	-0.33841	0.07206	-0.08243	0.18843	-0.29357	0.07363
n-propyl	aliphatic	-0.33828	0.07428	-0.08121	0.17438	-0.29256	0.07546
t-butyl	aliphatic	-0.33857	0.07477	-0.08406	0.18351	-0.30383	0.07418
p-methylphenyl	aromatic	-0.30309	0.06808	-0.09087	0.14946	-0.28913	0.07035
p-ethylphenyl	aromatic	-0.30318	0.06618	-0.09156	0.14344	-0.296	0.06673
p-fluorophenyl	aromatic	-0.31576	0.0643	-0.09751	0.1698	-0.29798	0.06697
p-chlorophenyl	aromatic	-0.3168	0.06266	-0.1016	0.1533	-0.2998	0.06417
p-methoxyphenyl	aromatic	-0.29375	0.06866	-0.09049	0.14403	-0.28246	0.06718
p-cyanophenyl	aromatic	-0.33266	0.0507	-0.11218	0.1525	-0.31016	0.05943

Glyoxylic acid	HOMO	LUMO
Neutral	-0.45302	0.05375
Protonated	-0.72186	-0.2152

4. General Information for the Synthesis

Chemicals were purchased from commercial suppliers and used without further purification. Thin layer chromatography (TLC) was performed on aluminium plates coated with 60 F254 silica from Merck. Flash chromatography was carried out using a Biotage SP4, Biotage Isolera or Varian automated flash system with Silicycle or GraceResolve normal phase silica gel pre-packed columns. Fractions were collected at 254 nm or if necessary on all wavelengths between 200 and 400 nm. Microwave irradiation was performed in a Biotage Initiator Sixty in sealed vials (Biotage microwave vials, Type I, Class A borosilicate, 28 mm outer diameter, 26 mm inner diameter, 83 mm long, round-bottom for 5-20 mL of total reaction volume; Biotage microwave vials, 16 mm outer diameter, 14 mm inner diameter, 83 mm long, round-bottom for 2-5 mL of total reaction volume). Reactions were irradiated at 2.45 GHz and were able to reach temperatures between 60 and 250 °C. Heating was at a rate of 2-5 °C/s and the pressure was able to reach 20 bars.

5. Analytical Equipment

Melting points were measured using a Stuart automatic melting point SMP40 apparatus or a Shanghai ShenGuang WRR apparatus. Fourier Transform InfraRed (FTIR) spectra were measured using an Agilent Cary 630 FTIR or a Bruker TENSOR II FTIR Spectrometer as a neat sample tableting with KBr. The abbreviations for peak description are as follows: b = broad; w = weak; m = medium and s = strong. Ultraviolet (UV) spectra were recorded on a Hitachi U-2900 spectrophotometer or an Agilent Cary 100 UV-Vis spectrophotometer and were performed in ethanol. High resolution mass spectrometry (HRMS) was provided by the ESPRC National Mass Spectrometry Service, University of Wales, Swansea, or the Mass Spectrometry Service, Department of Pharmacy, Naval Medical University and performed by Diya Lyu on an Agilent Technologies 6550 iFunnel Q-TOF LC-MS, or conducted using an Agilent 6550 iFunnel QTOF LC-MS with an Agilent 1260 Infinity UPLC system. The sample was eluted on Acquity UPLC BEH C18 (1.7µm, 2.1 x 50mm) with a flow rate of 0.7 mL/min and run at a gradient of 1.2 min 5-95% 0.1% aq. HCOOH in MeCN.

LC-MS analyses were conducted using a Waters Acquity UPLC system with photo diode array (PDA) and evaporating light scattering detector (ELSD) or using the ESI mass spectra which were performed by Zichao Ding on an Agilent Technologies 6120 Quadrupole LC-MS. When a 2 min gradient was used, the sample was eluted on an Acquity UPLC BEH C18, 1.7µm, 2.1 x 50mm, with a flow rate of 0.6 ml/min using 5-95% 0.1% HCOOH in MeCN. Analytical purity of compounds was determined using Waters XTerra RP18, 5 µm (4.6 × 150 mm) column at 1 ml/min using either 0.1% aq. ammonia and MeCN or 0.1% aq. HCOOH and MeCN with a gradient of 5-100% over 15 min. When a 12 min gradient was used, the sample was eluted on ZORBAX Eclipse XDB-C18, 3.5 µm, 4.6 x 100 mm, with a flow rate of 0.4 ml/min using 30-70% 0.1% HCOOH in MeCN.

^{1}H and ^{13}C NMR spectra were obtained using a Bruker Avance III 500 spectrometer using a frequency of 500 MHz, and 123 MHz, respectively, or using a Bruker Avance III 600 spectrometer operating at a frequency of 600 MHz, and 150 MHz, respectively. ^{19}F NMR spectra were acquired using the Bruker Avance III 300 spectrometer using a frequency of 282 MHz. The abbreviations for spin multiplicity are as follows: s = singlet; d = doublet; t = triplet; q = quartet, p = quintuplet, h = sextuplet and m = multiplet. Combinations of these abbreviations are employed to describe more complex splitting patterns (e.g. dd = doublet of doublets).

6. NMR spectra

Compound 1

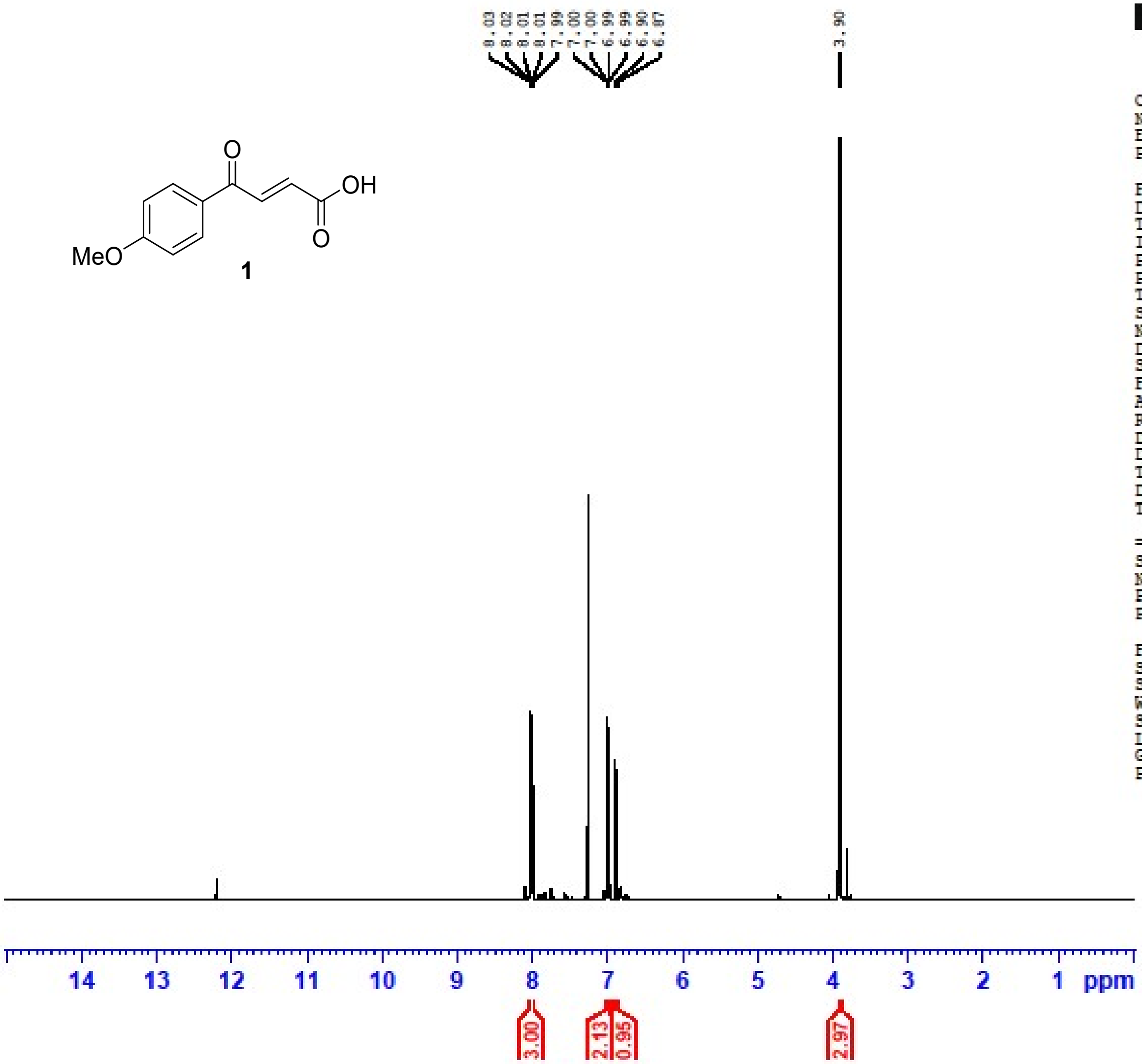

Current Data Parameters
NAME MU-646-33-F1
EXPNO 20
PROCNO 1

F2 - Acquisition Parameters
Date_ 20190524
Time 9.25
INSTRUM spect
PROBHD 5 mm PABBO BB-
PULPROG zg30
TD 65536
SOLVENT CDCl3
NS 16
DS 0
SWH 10330.578 Hz
FIDRES 0.157632 Hz
AQ 3.1719425 sec
RG 645
DW 48.400 usec
DE 12.02 usec
TE 295.9 K
D1 1.00000000 sec
TD0 1

======== CHANNEL f1 ========
SFO1 500.3030896 MHz
NUC1 1H
P1 16.10 usec
PLW1 19.50000000 W

F2 - Processing parameters
SI 65536
SF 500.3000000 MHz
WDW EM
SSB 0
LB 0.30 Hz
GB 0
PC 1.00

Compound 1

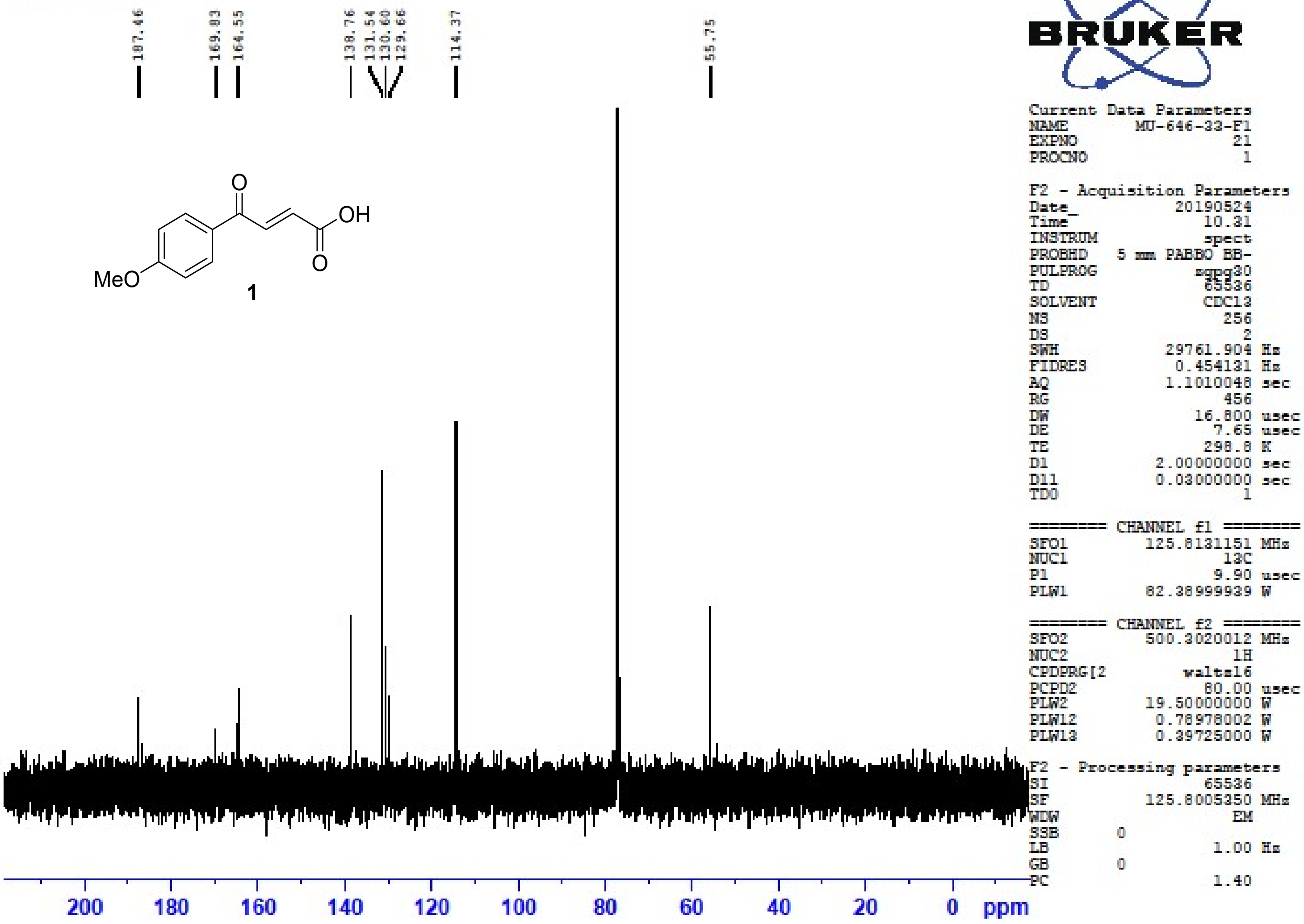
187.46
169.83
164.55
138.76
131.54
130.60
129.66
114.37
55.75
OH
O
O
MeO
1
BRUKER
Current Data Parameters
NAME MU-646-33-F1
EXPNO 21
PROCNO 1
F2 - Acquisition Parameters
Date_ 20190524
Time 10.31
INSTRUM spect
PROBHD 5 mm PABBO BB-
PULPROG zgpg30
TD 65536
SOLVENT CDCl3
NS 256
DS 2
SWH 29761.904 Hz
FIDRES 0.454131 Hz
AQ 1.1010048 sec
RG 456
DW 16.800 usec
DE 7.65 usec
TE 298.8 K
D1 2.00000000 sec
D11 0.03000000 sec
TD0 1
======== CHANNEL f1 ========
SFO1 125.8131151 MHz
NUC1 13C
P1 9.90 usec
PLW1 82.38999939 W
======== CHANNEL f2 ========
SFO2 500.3020012 MHz
NUC2 1H
CPDPRG[2 waltz16
PCPD2 80.00 usec
PLW2 19.50000000 W
PLW12 0.78978002 W
PLW13 0.39725000 W
F2 - Processing parameters
SI 65536
SF 125.8005350 MHz
WDW EM
SSB 0
LB 1.00 Hz
GB 0
PC 1.40
200
180
160
140
120
100
80
60
40
20
0
ppm

Compound 2

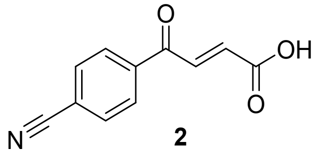

2

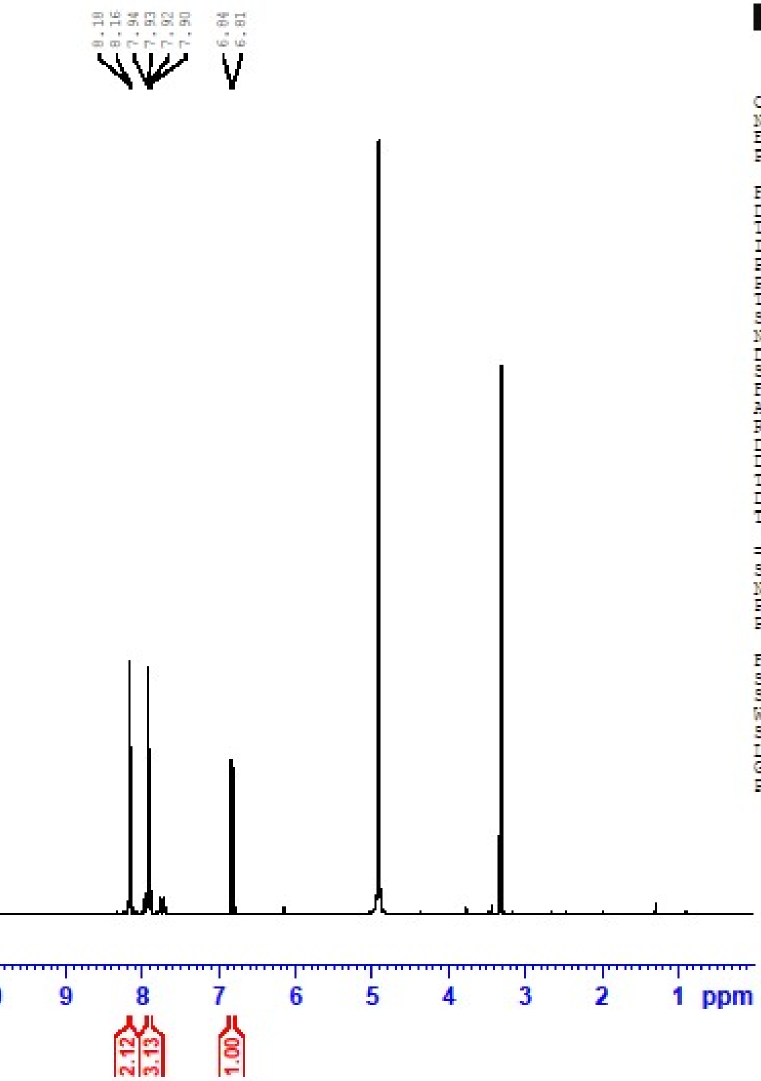

Current Data Parameters
NAME MU-666-123-F4
EXPNO 20
PROCNO 1

F2 - Acquisition Parameters
Date_ 20200214
Time 4.23
INSTRUM spect
PROBHD 5 mm PABBO BB-
PULPROG zg30
TD 65536
SOLVENT MeOD
NS 16
DS 0
SWH 10330.578 Hz
FIDRES 0.157632 Hz
AQ 3.1719425 sec
RG 512
DW 48.400 usec
DE 12.02 usec
TE 292.3 K
D1 1.00000000 sec
TD0 1

======== CHANNEL f1 ========
SFO1 500.3030896 MHz
NUC1 1H
P1 16.10 usec
PLW1 19.50000000 W

F2 - Processing parameters
SI 65536
SF 500.3000000 MHz
WDW EM
SSB 0
LB 0.30 Hz
GB 0
PC 1.00

Compound 2

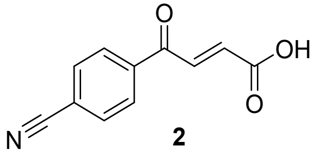

2

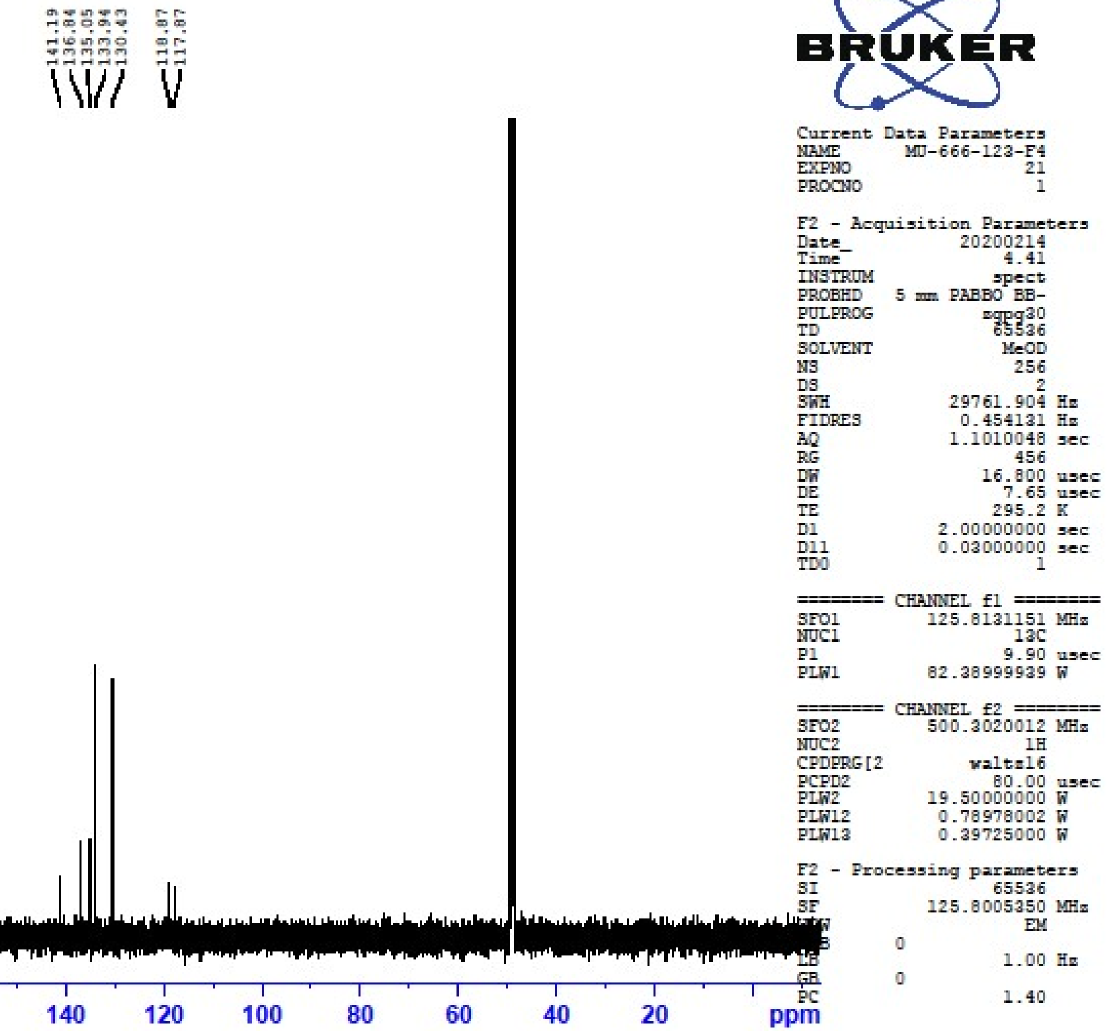
190.30
168.23
141.19
136.84
135.05
133.94
130.43
118.87
117.87
BRUKER
Current Data Parameters
NAME MU-666-123-F4
EXPNO 21
PROCNO 1
F2 - Acquisition Parameters
Date_ 20200214
Time 4.41
INSTRUM spect
PROBHD 5 mm PABBO BB-
PULPROG zgpg30
TD 65536
SOLVENT MeOD
NS 256
DS 2
SWH 29761.904 Hz
FIDRES 0.454131 Hz
AQ 1.1010048 sec
RG 456
DW 16.800 usec
DE 7.65 usec
TE 295.2 K
D1 2.00000000 sec
D11 0.03000000 sec
TD0 1
======== CHANNEL f1 ========
SFO1 125.8131151 MHz
NUC1 13C
P1 9.90 usec
PLW1 82.38999939 W
======== CHANNEL f2 ========
SFO2 500.3020012 MHz
NUC2 1H
CPDPRG[2 waltz16
PCPD2 80.00 usec
PLW2 19.50000000 W
PLW12 0.78978002 W
PLW13 0.39725000 W
F2 - Processing parameters
SI 65536
SF 125.8005350 MHz
EM
0
LB 1.00 Hz
GB 0
PC 1.40
200
180
160
140
120
100
80
60
40
20
ppm

Compound 3

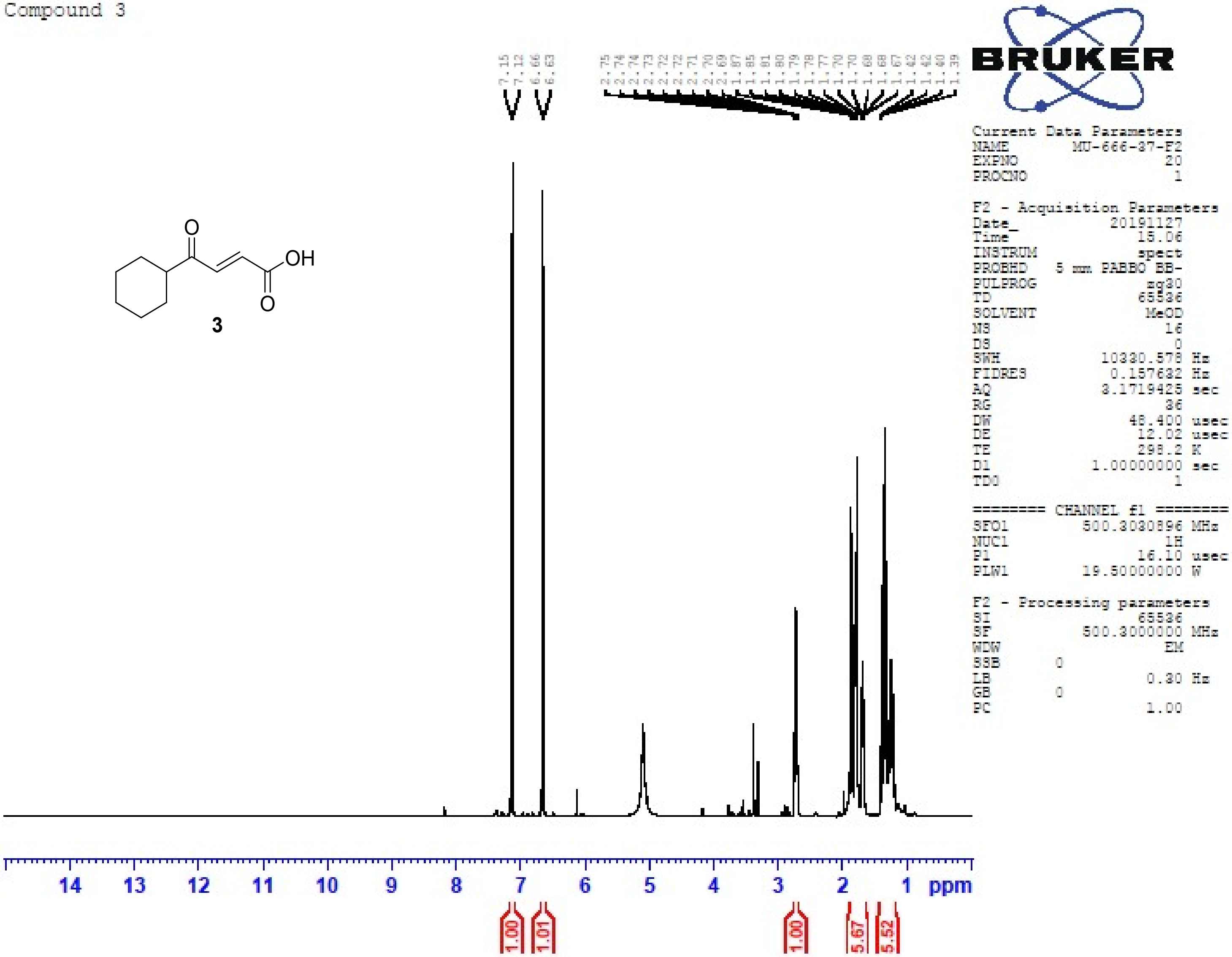
O
OH
O
3
7.15
7.12
6.66
6.63
2.75
2.74
2.74
2.73
2.72
2.72
2.71
2.70
2.69
1.87
1.85
1.81
1.80
1.79
1.78
1.77
1.70
1.70
1.68
1.67
1.42
1.40
1.39
BRUKER
Current Data Parameters
NAME MU-666-37-F2
EXPNO 20
PROCNO 1
F2 - Acquisition Parameters
Date_ 20191127
Time 15.06
INSTRUM spect
PROBHD 5 mm PABBO BB-
PULPROG zg30
TD 65536
SOLVENT MeOD
NS 16
DS 0
SWH 10330.578 Hz
FIDRES 0.157632 Hz
AQ 3.1719425 sec
RG 36
DW 48.400 usec
DE 12.02 usec
TE 298.2 K
D1 1.00000000 sec
TD0 1
======== CHANNEL f1 ========
SFO1 500.3030896 MHz
NUC1 1H
P1 16.10 usec
PLW1 19.50000000 W
F2 - Processing parameters
SI 65536
SF 500.3000000 MHz
WDW EM
SSB 0
LB 0.30 Hz
GB 0
PC 1.00
14 13 12 11 10 9 8 7 6 5 4 3 2 1 ppm
1.00
1.01
1.00
5.67
5.52

Compound 3

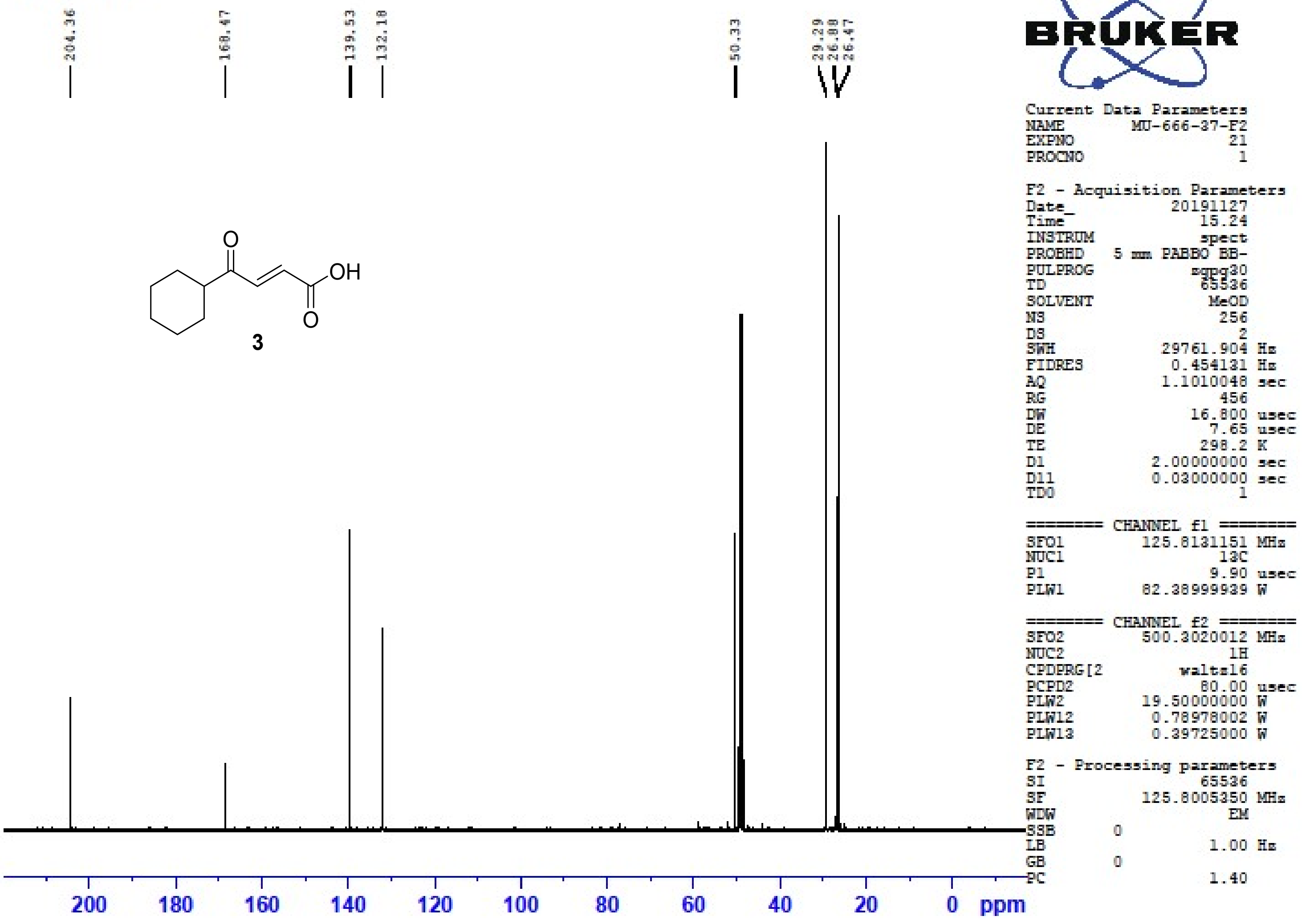
204.36
168.47
139.53
132.18
50.33
29.29
26.88
26.47
O
OH
O
3
200
180
160
140
120
100
80
60
40
20
0
ppm
BRUKER
Current Data Parameters
NAME MU-666-37-F2
EXPNO 21
PROCNO 1
F2 - Acquisition Parameters
Date_ 20191127
Time 15.24
INSTRUM spect
PROBHD 5 mm PABBO BB-
PULPROG zgpg30
TD 65536
SOLVENT MeOD
NS 256
DS 2
SWH 29761.904 Hz
FIDRES 0.454131 Hz
AQ 1.1010048 sec
RG 456
DW 16.800 usec
DE 7.65 usec
TE 298.2 K
D1 2.00000000 sec
D11 0.03000000 sec
TD0 1
======== CHANNEL f1 ========
SFO1 125.8131151 MHz
NUC1 13C
P1 9.90 usec
PLW1 82.38999939 W
======== CHANNEL f2 ========
SFO2 500.3020012 MHz
NUC2 1H
CPDPRG[2 waltz16
PCPD2 80.00 usec
PLW2 19.50000000 W
PLW12 0.78978002 W
PLW13 0.39725000 W
F2 - Processing parameters
SI 65536
SF 125.8005350 MHz
WDW EM
SSB 0
LB 1.00 Hz
GB 0
PC 1.40

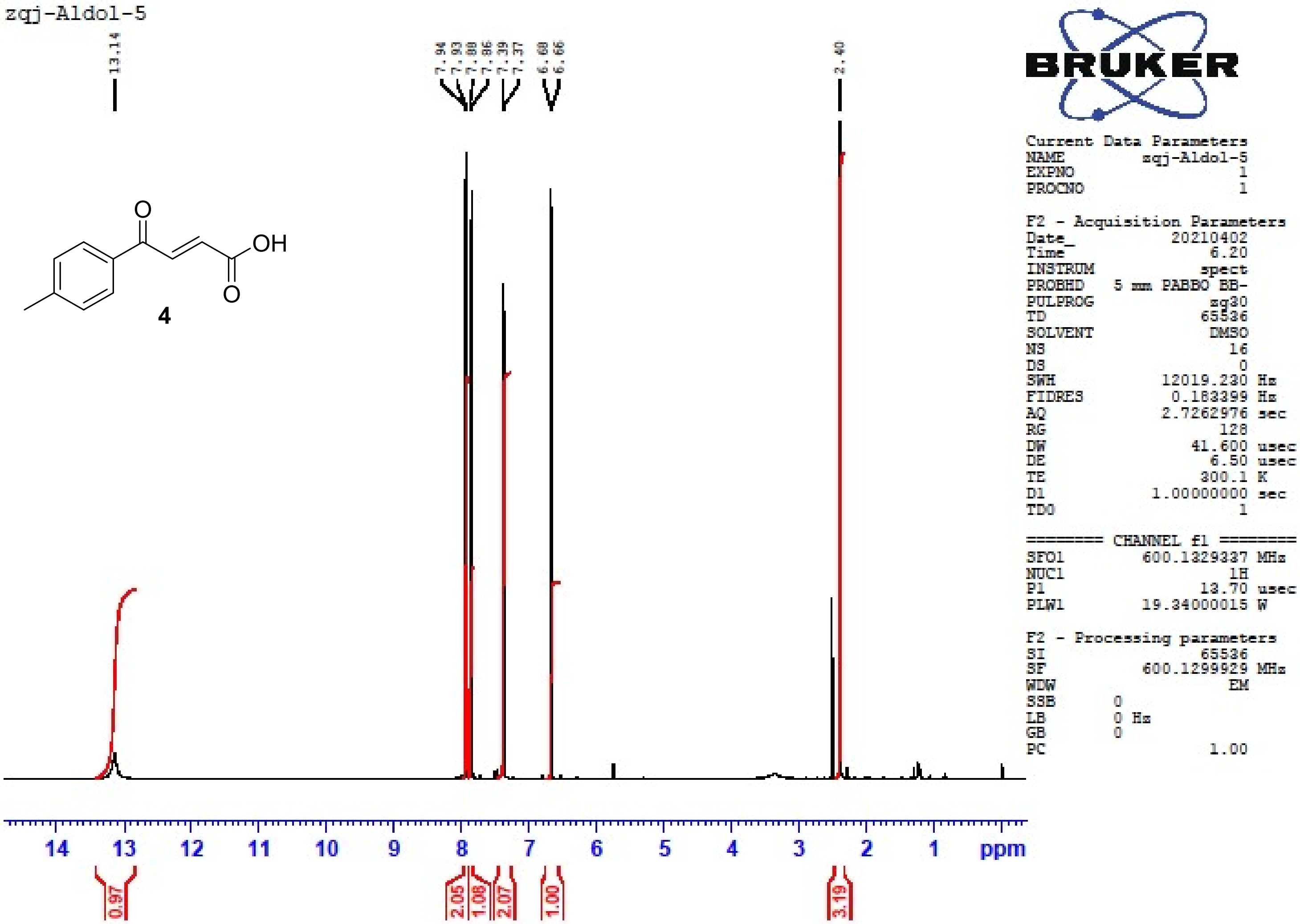
zqj-Aldol-5
13.14
7.94 7.93 7.88 7.86 7.39 7.37
6.68 6.66
2.40
O
OH
O
4
BRUKER
Current Data Parameters
NAME zqj-Aldol-5
EXPNO 1
PROCNO 1
F2 - Acquisition Parameters
Date_ 20210402
Time 6.20
INSTRUM spect
PROBHD 5 mm PABBO BB-
PULPROG zg30
TD 65536
SOLVENT DMSO
NS 16
DS 0
SWH 12019.230 Hz
FIDRES 0.183399 Hz
AQ 2.7262976 sec
RG 128
DW 41.600 usec
DE 6.50 usec
TE 300.1 K
D1 1.00000000 sec
TD0 1
======== CHANNEL f1 ========
SFO1 600.1329337 MHz
NUC1 1H
P1 13.70 usec
PLW1 19.34000015 W
F2 - Processing parameters
SI 65536
SF 600.1299929 MHz
WDW EM
SSB 0
LB 0 Hz
GB 0
PC 1.00
14 13 12 11 10 9 8 7 6 5 4 3 2 1 ppm
0.97
2.05 1.08 2.07
1.00
3.19

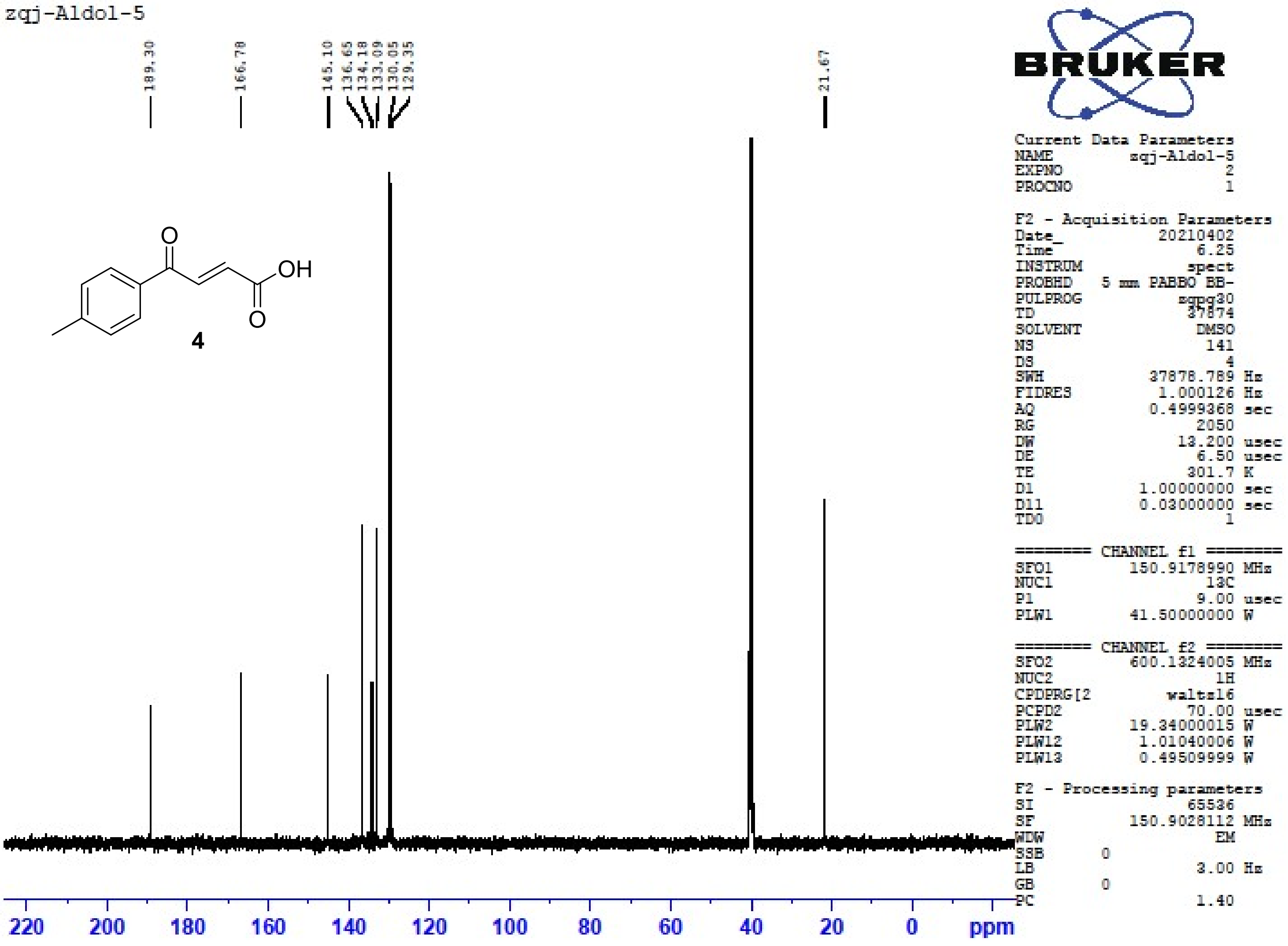
zqj-Aldol-5
189.30
166.78
145.10
136.65
134.18
133.09
130.05
129.35
21.67
OH
O
O
4
220 200 180 160 140 120 100 80 60 40 20 0 ppm
BRUKER
Current Data Parameters
NAME zqj-Aldol-5
EXPNO 2
PROCNO 1
F2 - Acquisition Parameters
Date_ 20210402
Time 6.25
INSTRUM spect
PROBHD 5 mm PABBO BB-
PULPROG zgpg30
TD 37874
SOLVENT DMSO
NS 141
DS 4
SWH 37878.789 Hz
FIDRES 1.000126 Hz
AQ 0.4999368 sec
RG 2050
DW 13.200 usec
DE 6.50 usec
TE 301.7 K
D1 1.00000000 sec
D11 0.03000000 sec
TD0 1
======== CHANNEL f1 ========
SFO1 150.9178990 MHz
NUC1 13C
P1 9.00 usec
PLW1 41.50000000 W
======== CHANNEL f2 ========
SFO2 600.1324005 MHz
NUC2 1H
CPDPRG[2 waltz16
PCPD2 70.00 usec
PLW2 19.34000015 W
PLW12 1.01040006 W
PLW13 0.49509999 W
F2 - Processing parameters
SI 65536
SF 150.9028112 MHz
WDW EM
SSB 0
LB 3.00 Hz
GB 0
PC 1.40

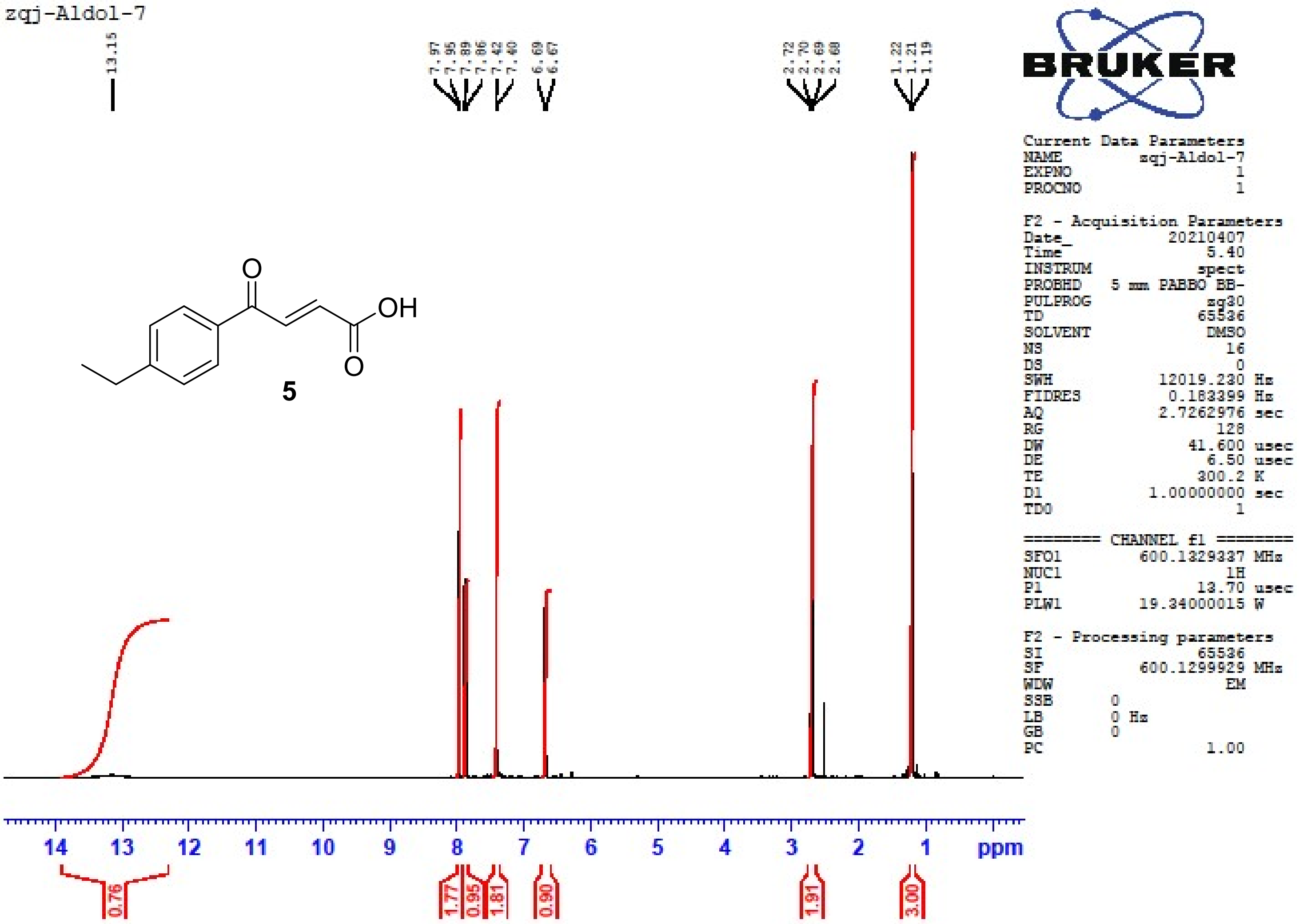
zqj-Aldol-7
13.15
7.97 7.95 7.89 7.86 7.42 7.40 6.69 6.67
2.72 2.70 2.69 2.68
1.22 1.21 1.19
O
OH
O
5
BRUKER
Current Data Parameters
NAME zqj-Aldol-7
EXPNO 1
PROCNO 1
F2 - Acquisition Parameters
Date_ 20210407
Time 5.40
INSTRUM spect
PROBHD 5 mm PABBO BB-
PULPROG zg30
TD 65536
SOLVENT DMSO
NS 16
DS 0
SWH 12019.230 Hz
FIDRES 0.183399 Hz
AQ 2.7262976 sec
RG 128
DW 41.600 usec
DE 6.50 usec
TE 300.2 K
D1 1.00000000 sec
TD0 1
======== CHANNEL f1 ========
SFO1 600.1329337 MHz
NUC1 1H
P1 13.70 usec
PLW1 19.34000015 W
F2 - Processing parameters
SI 65536
SF 600.1299929 MHz
WDW EM
SSB 0
LB 0 Hz
GB 0
PC 1.00
14 13 12 11 10 9 8 7 6 5 4 3 2 1 ppm
0.76
1.77 0.95 1.81 0.90
1.91
3.00

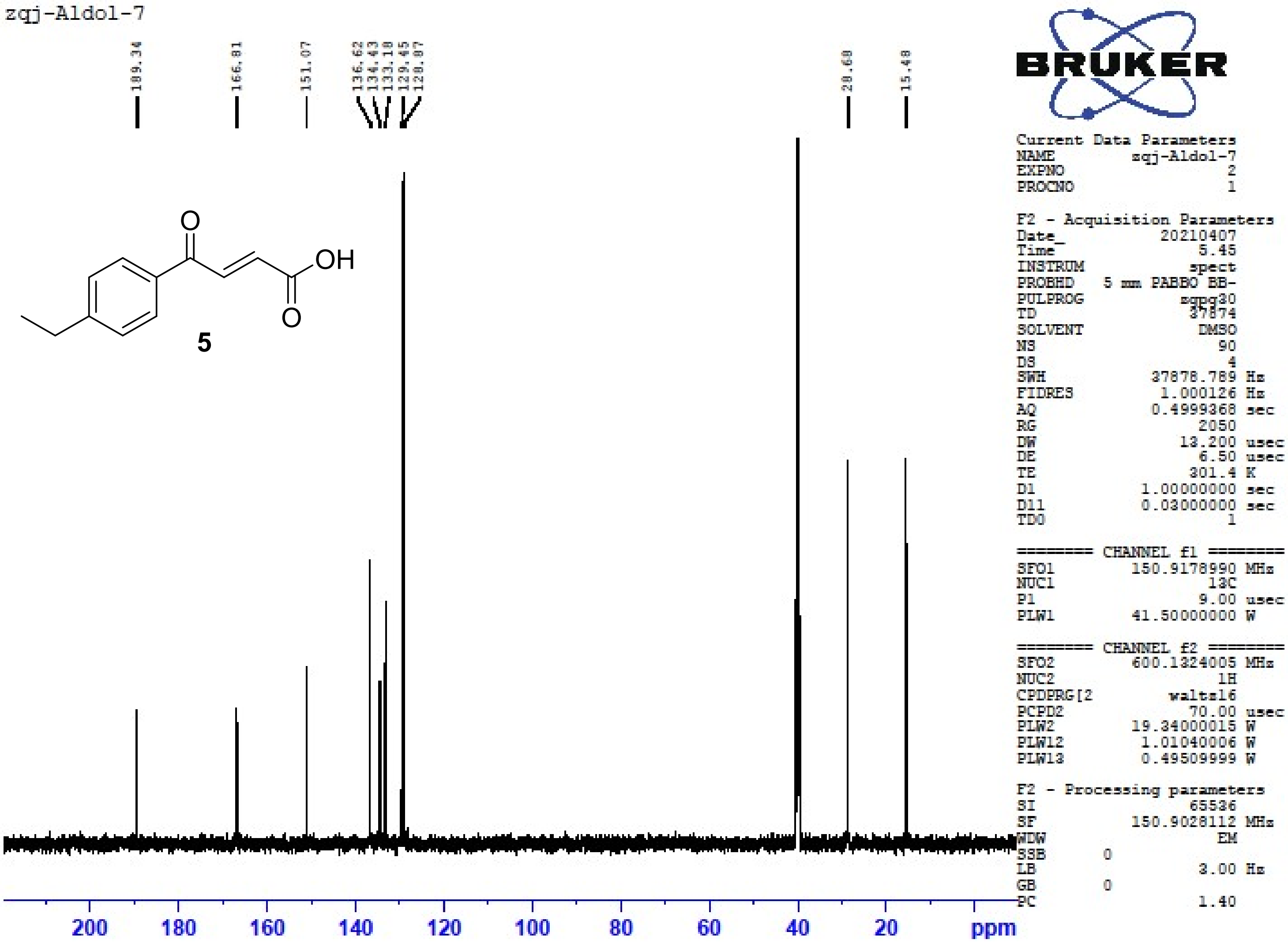
zqj-Aldol-7
189.34
166.81
151.07
136.62
134.43
133.18
129.45
128.87
28.68
15.48
O
OH
O
5
200
180
160
140
120
100
80
60
40
20
ppm
Current Data Parameters
NAME zqj-Aldol-7
EXPNO 2
PROCNO 1
F2 - Acquisition Parameters
Date_ 20210407
Time 5.45
INSTRUM spect
PROBHD 5 mm PABBO BB-
PULPROG zgpg30
TD 37874
SOLVENT DMSO
NS 90
DS 4
SWH 37878.789 Hz
FIDRES 1.000126 Hz
AQ 0.4999368 sec
RG 2050
DW 13.200 usec
DE 6.50 usec
TE 301.4 K
D1 1.00000000 sec
D11 0.03000000 sec
TD0 1
======== CHANNEL f1 ========
SFO1 150.9178990 MHz
NUC1 13C
P1 9.00 usec
PLW1 41.50000000 W
======== CHANNEL f2 ========
SFO2 600.1324005 MHz
NUC2 1H
CPDPRG[2 waltz16
PCPD2 70.00 usec
PLW2 19.34000015 W
PLW12 1.01040006 W
PLW13 0.49509999 W
F2 - Processing parameters
SI 65536
SF 150.9028112 MHz
WDW EM
SSB 0
LB 3.00 Hz
GB 0
PC 1.40

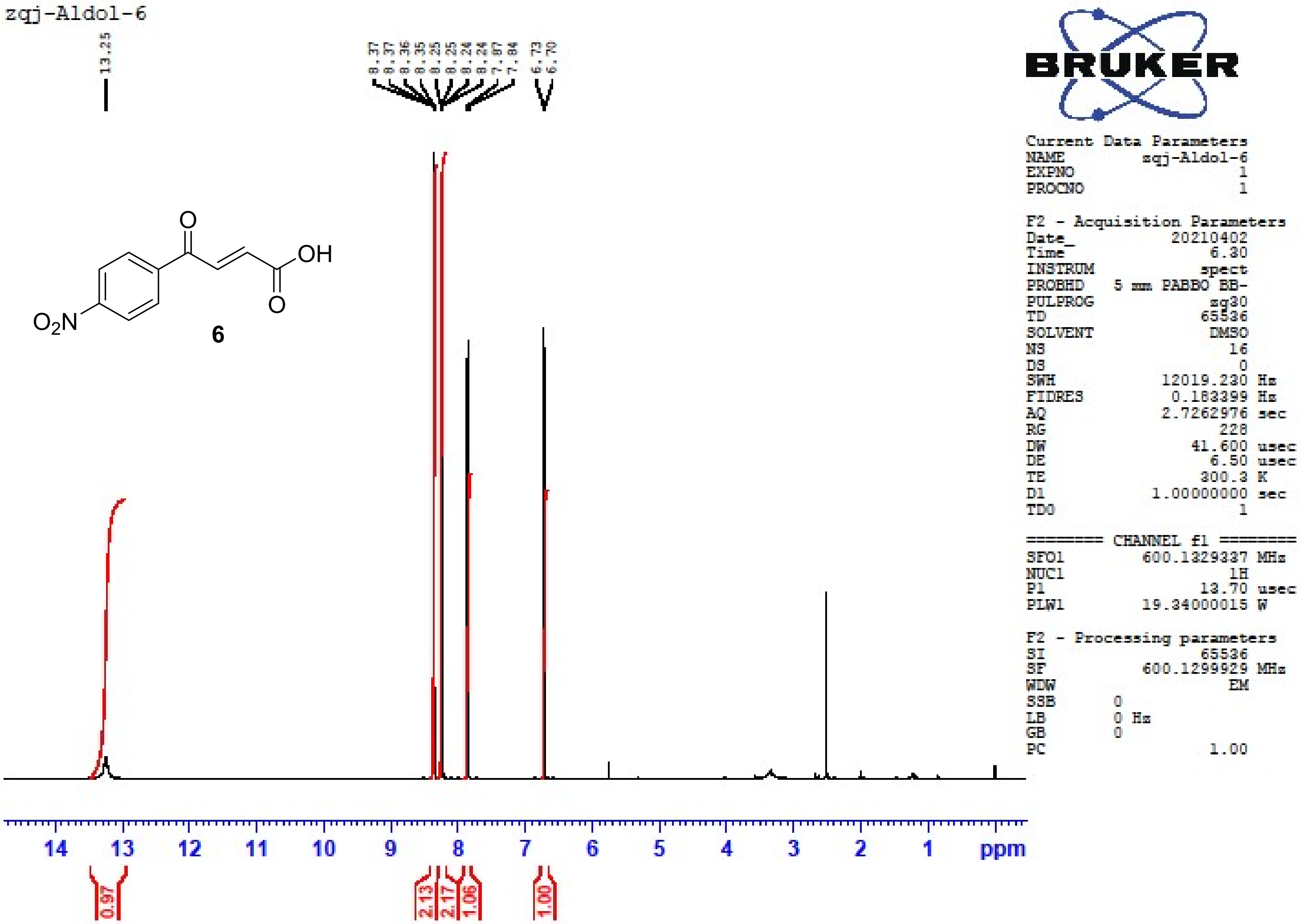
zqj-Aldol-6
13.25
8.37 8.37 8.36 8.35 8.25 8.25 8.24 8.24 7.87 7.84
6.73 6.70
OH
O
O
O2N
6
BRUKER
Current Data Parameters
NAME zqj-Aldol-6
EXPNO 1
PROCNO 1
F2 - Acquisition Parameters
Date_ 20210402
Time 6.30
INSTRUM spect
PROBHD 5 mm PABBO BB-
PULPROG zg30
TD 65536
SOLVENT DMSO
NS 16
DS 0
SWH 12019.230 Hz
FIDRES 0.183399 Hz
AQ 2.7262976 sec
RG 228
DW 41.600 usec
DE 6.50 usec
TE 300.3 K
D1 1.00000000 sec
TD0 1
======== CHANNEL f1 ========
SFO1 600.1329337 MHz
NUC1 1H
P1 13.70 usec
PLW1 19.34000015 W
F2 - Processing parameters
SI 65536
SF 600.1299929 MHz
WDW EM
SSB 0
LB 0 Hz
GB 0
PC 1.00
14 13 12 11 10 9 8 7 6 5 4 3 2 1 ppm
0.97
2.13 2.17 1.06
1.00

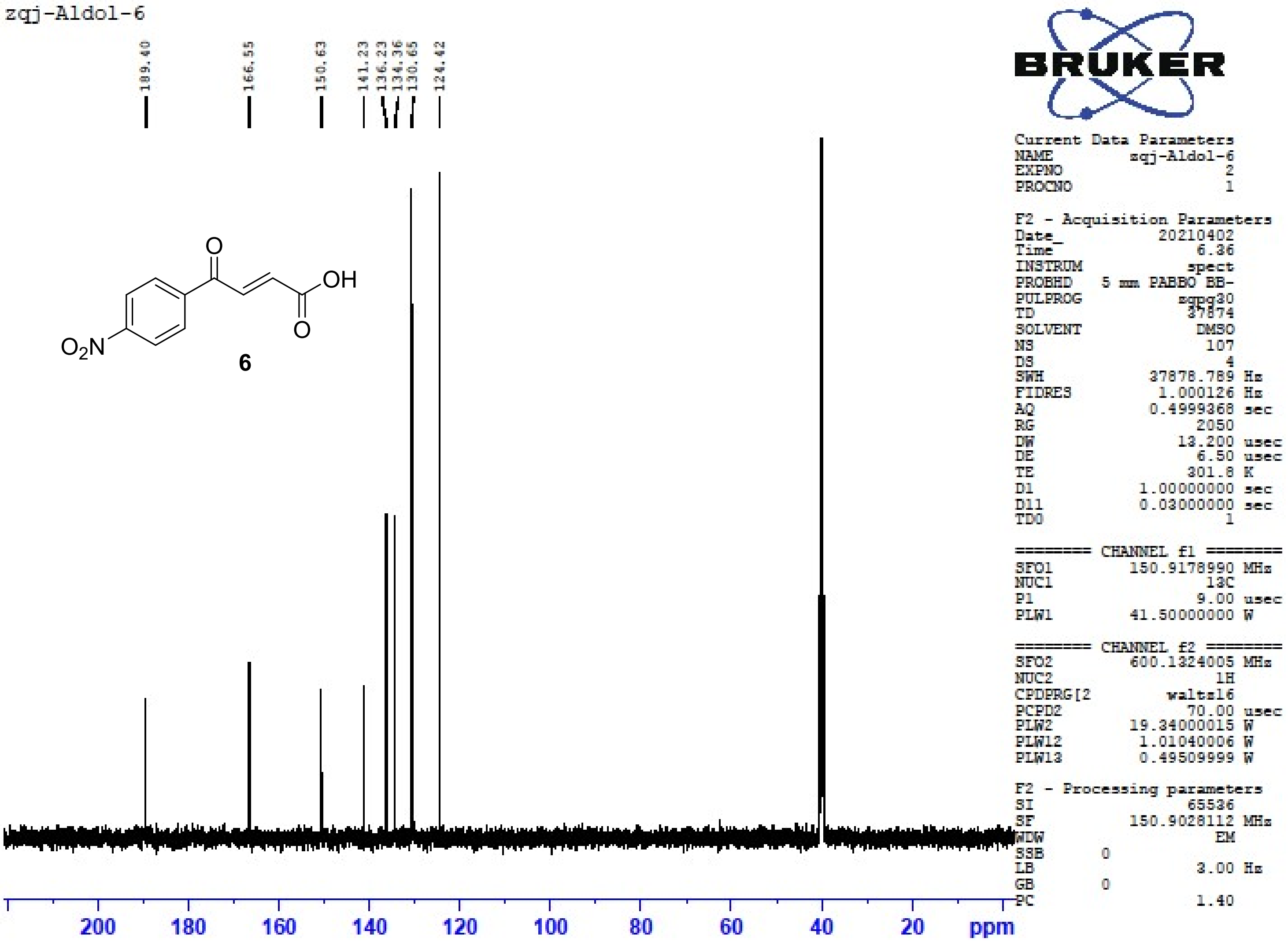
zqj-Aldol-6
189.40
166.55
150.63
141.23
136.23
134.36
130.65
124.42
O
OH
O
O_2N
6
BRUKER
Current Data Parameters
NAME zqj-Aldol-6
EXPNO 2
PROCNO 1
F2 - Acquisition Parameters
Date_ 20210402
Time 6.36
INSTRUM spect
PROBHD 5 mm PABBO BB-
PULPROG zgpg30
TD 37874
SOLVENT DMSO
NS 107
DS 4
SWH 37878.789 Hz
FIDRES 1.000126 Hz
AQ 0.4999368 sec
RG 2050
DW 13.200 usec
DE 6.50 usec
TE 301.8 K
D1 1.00000000 sec
D11 0.03000000 sec
TD0 1
======== CHANNEL f1 ========
SFO1 150.9178990 MHz
NUC1 13C
P1 9.00 usec
PLW1 41.50000000 W
======== CHANNEL f2 ========
SFO2 600.1324005 MHz
NUC2 1H
CPDPRG[2 waltz16
PCPD2 70.00 usec
PLW2 19.34000015 W
PLW12 1.01040006 W
PLW13 0.49509999 W
F2 - Processing parameters
SI 65536
SF 150.9028112 MHz
WDW EM
SSB 0
LB 3.00 Hz
GB 0
PC 1.40
200 180 160 140 120 100 80 60 40 20 ppm

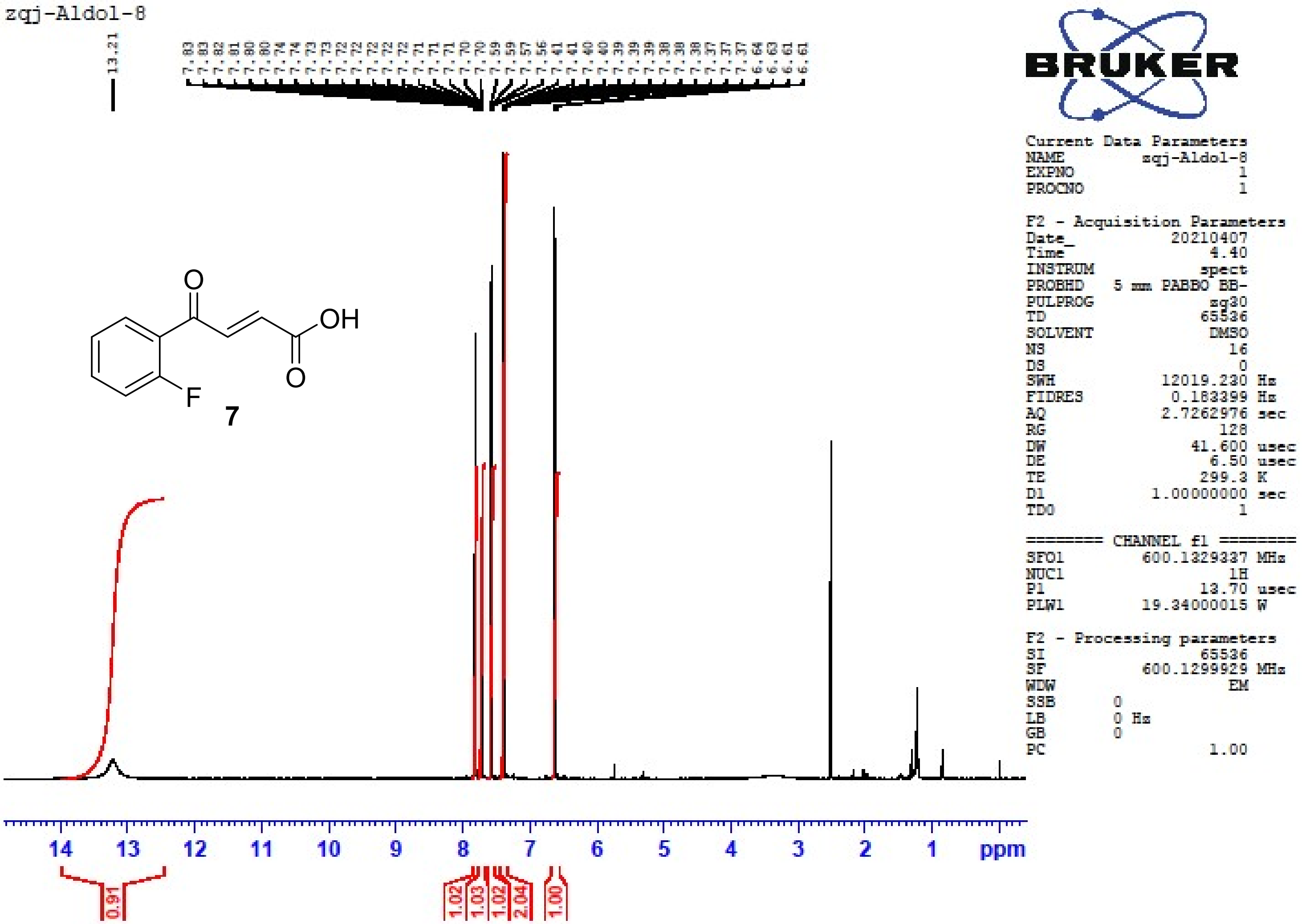
zqj-Aldol-8
13.21
OH
O
F
7
BRUKER
Current Data Parameters
NAME zqj-Aldol-8
EXPNO 1
PROCNO 1
F2 - Acquisition Parameters
Date_ 20210407
Time 4.40
INSTRUM spect
PROBHD 5 mm PABBO BB-
PULPROG zg30
TD 65536
SOLVENT DMSO
NS 16
DS 0
SWH 12019.230 Hz
FIDRES 0.183399 Hz
AQ 2.7262976 sec
RG 128
DW 41.600 usec
DE 6.50 usec
TE 299.3 K
D1 1.00000000 sec
TD0 1
======== CHANNEL f1 ========
SFO1 600.1329337 MHz
NUC1 1H
P1 13.70 usec
PLW1 19.34000015 W
F2 - Processing parameters
SI 65536
SF 600.1299929 MHz
WDW EM
SSB 0
LB 0 Hz
GB 0
PC 1.00
14 13 12 11 10 9 8 7 6 5 4 3 2 1 ppm
0.91
1.02 1.03 1.02 2.04 1.00

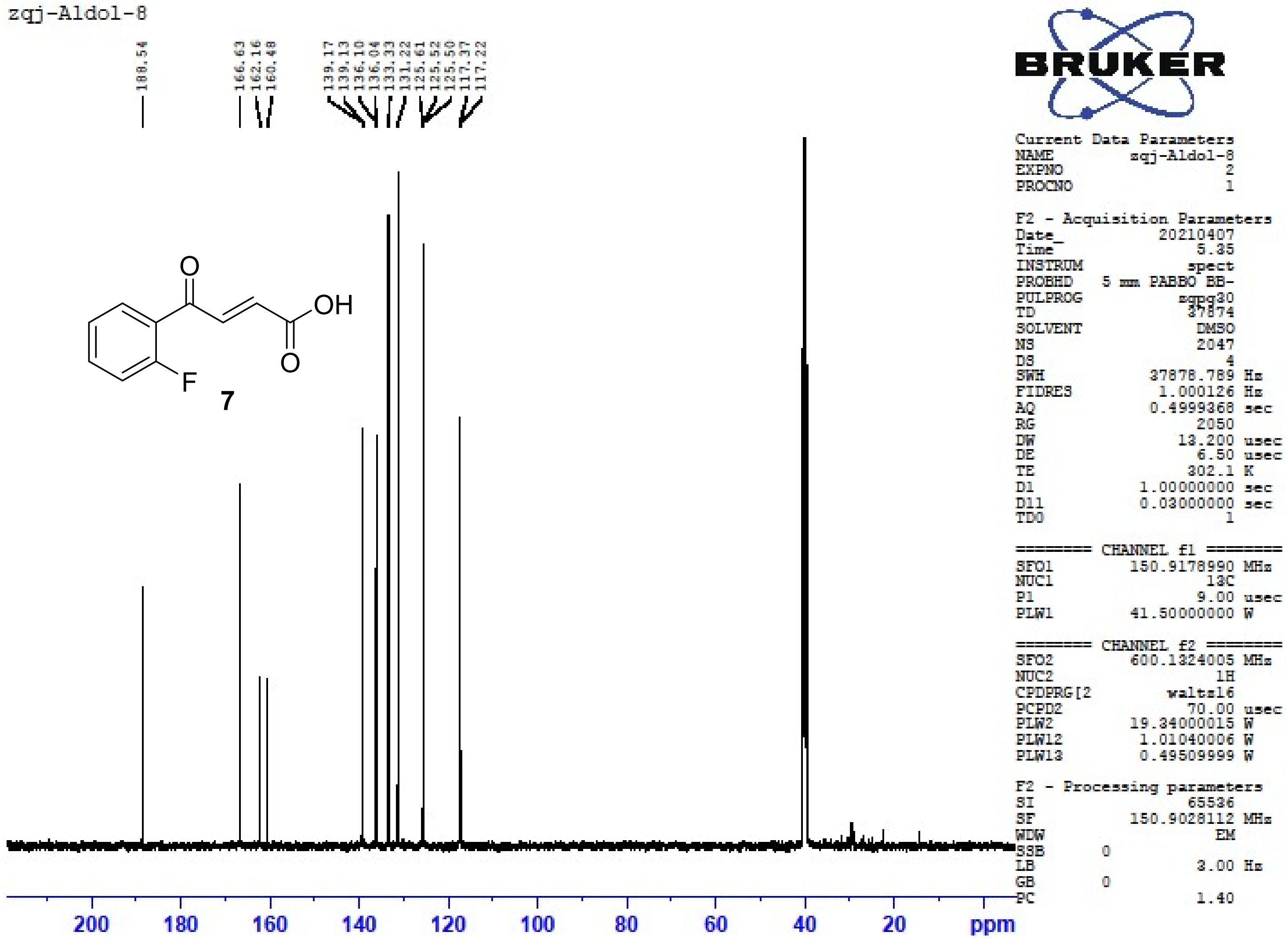

zqj-Aldol-8
188.54
166.63
162.16
160.48
139.17
139.13
136.10
136.04
133.33
131.22
125.61
125.52
125.50
117.37
117.22
OH
O
O
F
7
BRUKER
Current Data Parameters
NAME zqj-Aldol-8
EXPNO 2
PROCNO 1
F2 - Acquisition Parameters
Date_ 20210407
Time 5.35
INSTRUM spect
PROBHD 5 mm PABBO BB-
PULPROG zgpg30
TD 37874
SOLVENT DMSO
NS 2047
DS 4
SWH 37878.789 Hz
FIDRES 1.000126 Hz
AQ 0.4999368 sec
RG 2050
DW 13.200 usec
DE 6.50 usec
TE 302.1 K
D1 1.00000000 sec
D11 0.03000000 sec
TD0 1
======== CHANNEL f1 ========
SFO1 150.9178990 MHz
NUC1 13C
P1 9.00 usec
PLW1 41.50000000 W
======== CHANNEL f2 ========
SFO2 600.1324005 MHz
NUC2 1H
CPDPRG[2 waltz16
PCPD2 70.00 usec
PLW2 19.34000015 W
PLW12 1.01040006 W
PLW13 0.49509999 W
F2 - Processing parameters
SI 65536
SF 150.9028112 MHz
WDW EM
SSB 0
LB 3.00 Hz
GB 0
PC 1.40
200 180 160 140 120 100 80 60 40 20 ppm

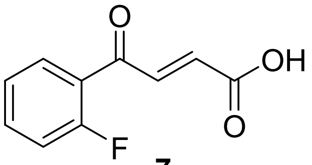

7

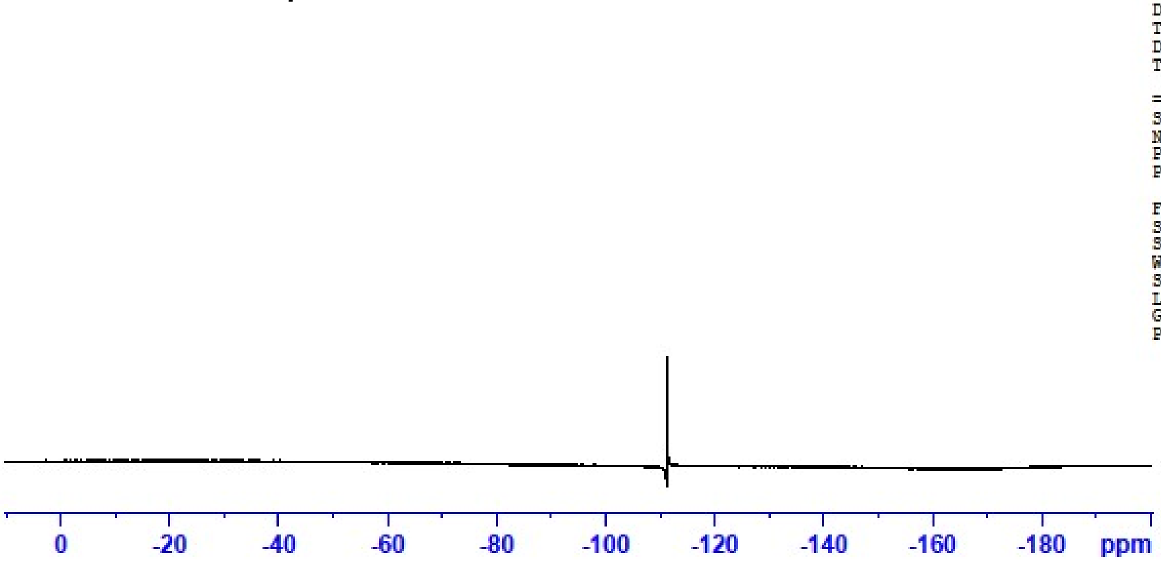

BRUKER

```
Current Data Parameters
NAME                A-8-F
EXPNO                   3
PROCNO                  1

F2 - Acquisition Parameters
Date_            20210506
Time                16.45
INSTRUM             spect
PROBHD   5 mm PABBO BB-
PULPROG            zgflqn
TD                 131072
SOLVENT              DMSO
NS                     32
DS                      4
SWH             66964.289 Hz
FIDRES           0.510897 Hz
AQ              0.9786710 sec
RG                    128
DW                  7.467 usec
DE                   6.50 usec
TE                  300.0 K
D1             1.00000000 sec
TD0                     1

======== CHANNEL f1 ========
SFO1          282.3761148 MHz
NUC1                  19F
P1                  19.50 usec
PLW1          11.00000000 W

F2 - Processing parameters
SI                  65536
SF            282.4043550 MHz
WDW                    EM
SSB      0
LB                   3.00 Hz
GB       0
PC                   1.00
```

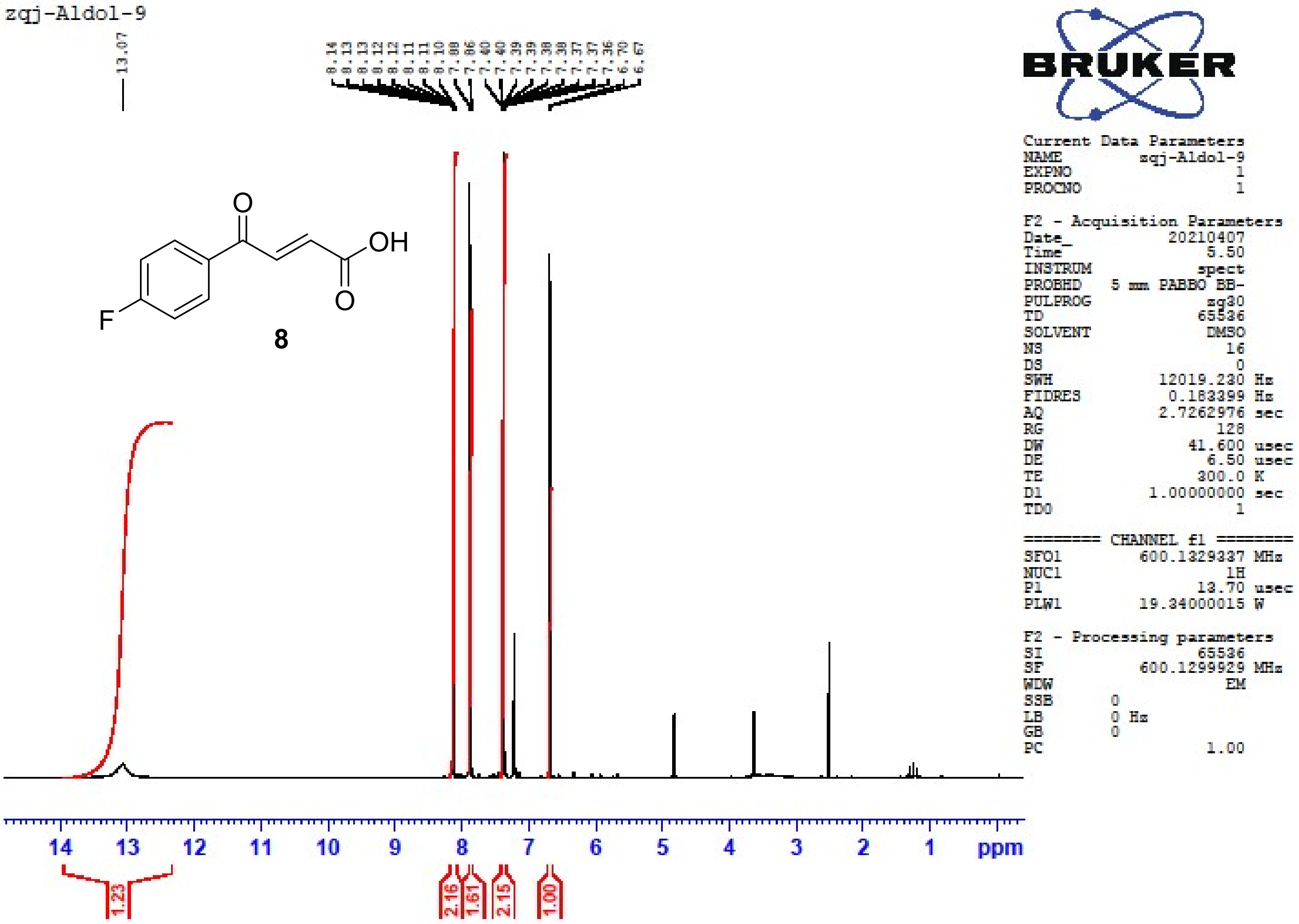
zqj-Aldol-9
13.07
8.14 8.13 8.13 8.12 8.12 8.11 8.11 8.10 7.88 7.86 7.40 7.40 7.39 7.39 7.38 7.38 7.37 7.36 6.70 6.67
O
OH
F
O
8
14 13 12 11 10 9 8 7 6 5 4 3 2 1 ppm
1.23
2.16
1.61
2.15
1.00
BRUKER
Current Data Parameters
NAME zqj-Aldol-9
EXPNO 1
PROCNO 1
F2 - Acquisition Parameters
Date_ 20210407
Time 5.50
INSTRUM spect
PROBHD 5 mm PABBO BB-
PULPROG zg30
TD 65536
SOLVENT DMSO
NS 16
DS 0
SWH 12019.230 Hz
FIDRES 0.183399 Hz
AQ 2.7262976 sec
RG 128
DW 41.600 usec
DE 6.50 usec
TE 300.0 K
D1 1.00000000 sec
TD0 1
======== CHANNEL f1 ========
SFO1 600.1329337 MHz
NUC1 1H
P1 13.70 usec
PLW1 19.34000015 W
F2 - Processing parameters
SI 65536
SF 600.1299929 MHz
WDW EM
SSB 0
LB 0 Hz
GB 0
PC 1.00

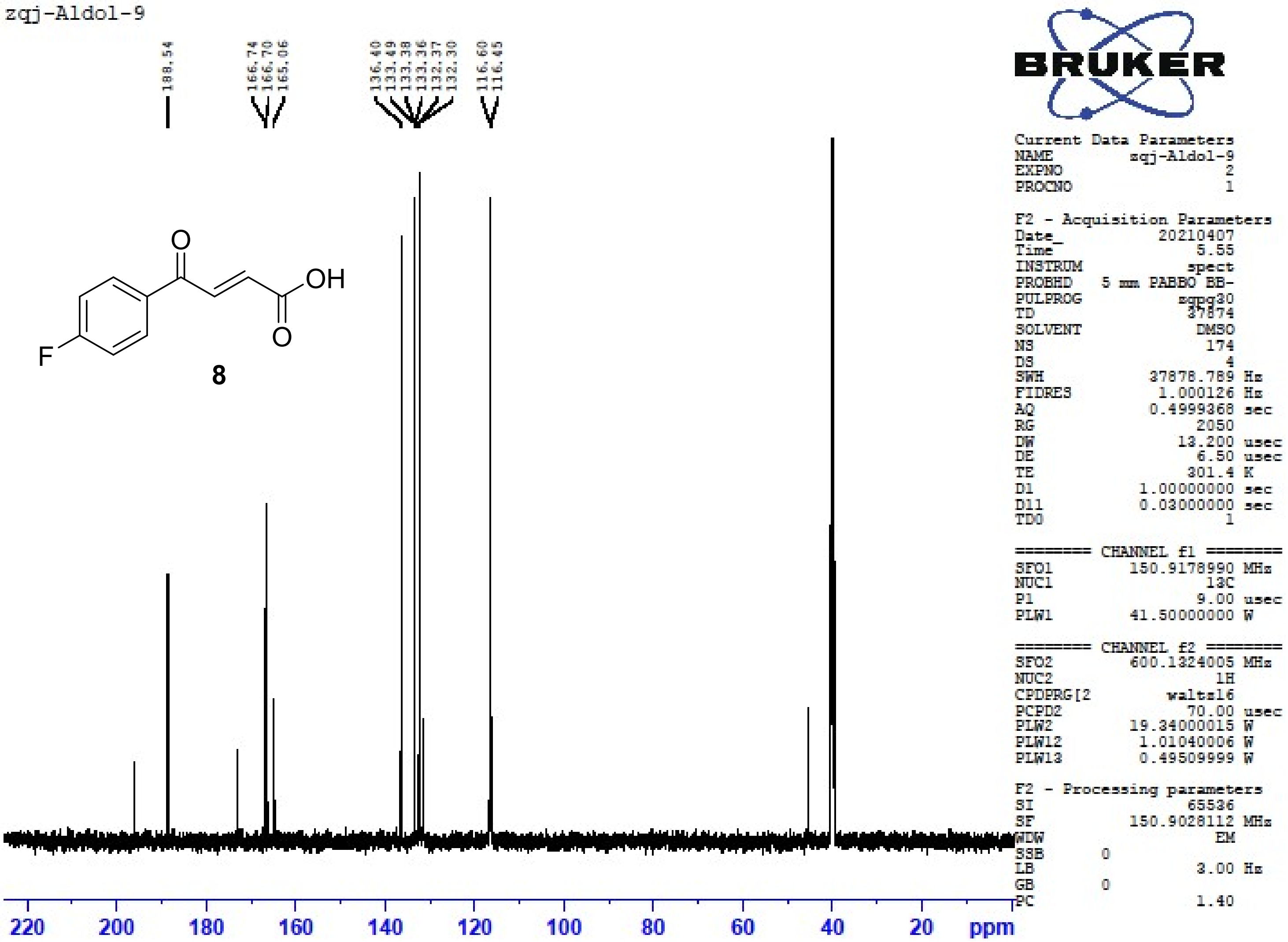
zqj-Aldol-9
188.54
166.74
166.70
165.06
136.40
133.49
133.38
133.36
132.37
132.30
116.60
116.45
O
OH
O
F
8
BRUKER
Current Data Parameters
NAME zqj-Aldol-9
EXPNO 2
PROCNO 1
F2 - Acquisition Parameters
Date_ 20210407
Time 5.55
INSTRUM spect
PROBHD 5 mm PABBO BB-
PULPROG zgpg30
TD 37874
SOLVENT DMSO
NS 174
DS 4
SWH 37878.789 Hz
FIDRES 1.000126 Hz
AQ 0.4999368 sec
RG 2050
DW 13.200 usec
DE 6.50 usec
TE 301.4 K
D1 1.00000000 sec
D11 0.03000000 sec
TD0 1
======== CHANNEL f1 ========
SFO1 150.9178990 MHz
NUC1 13C
P1 9.00 usec
PLW1 41.50000000 W
======== CHANNEL f2 ========
SFO2 600.1324005 MHz
NUC2 1H
CPDPRG[2 waltz16
PCPD2 70.00 usec
PLW2 19.34000015 W
PLW12 1.01040006 W
PLW13 0.49509999 W
F2 - Processing parameters
SI 65536
SF 150.9028112 MHz
WDW EM
SSB 0
LB 3.00 Hz
GB 0
PC 1.40
220
200
180
160
140
120
100
80
60
40
20
ppm

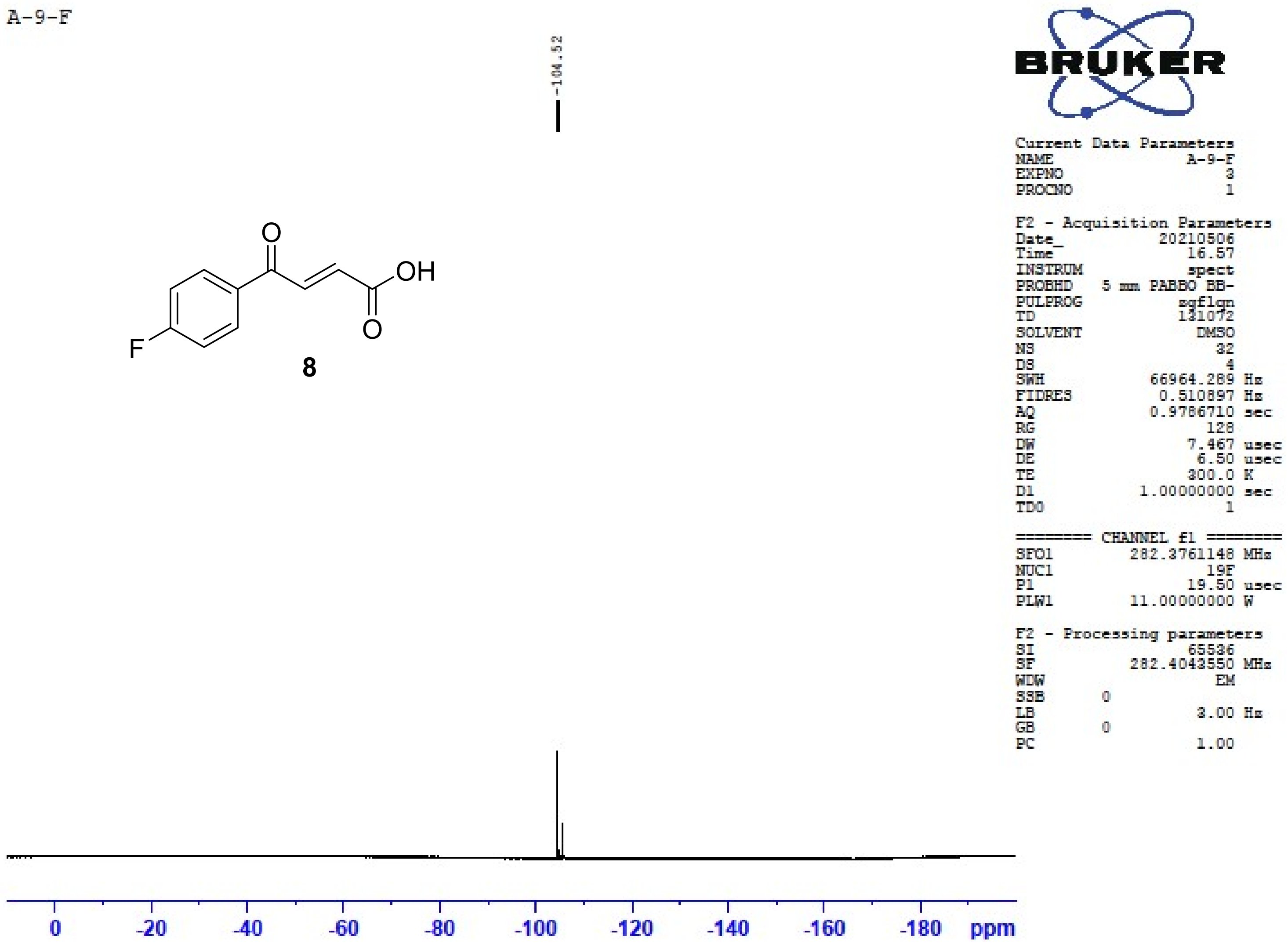
A-9-F
-104.52
O
OH
O
F
8
Current Data Parameters
NAME A-9-F
EXPNO 3
PROCNO 1
F2 - Acquisition Parameters
Date_ 20210506
Time 16.57
INSTRUM spect
PROBHD 5 mm PABBO BB-
PULPROG zgflqn
TD 131072
SOLVENT DMSO
NS 32
DS 4
SWH 66964.289 Hz
FIDRES 0.510897 Hz
AQ 0.9786710 sec
RG 128
DW 7.467 usec
DE 6.50 usec
TE 300.0 K
D1 1.00000000 sec
TD0 1
======== CHANNEL f1 ========
SFO1 282.3761148 MHz
NUC1 19F
P1 19.50 usec
PLW1 11.00000000 W
F2 - Processing parameters
SI 65536
SF 282.4043550 MHz
WDW EM
SSB 0
LB 3.00 Hz
GB 0
PC 1.00
0
-20
-40
-60
-80
-100
-120
-140
-160
-180
ppm

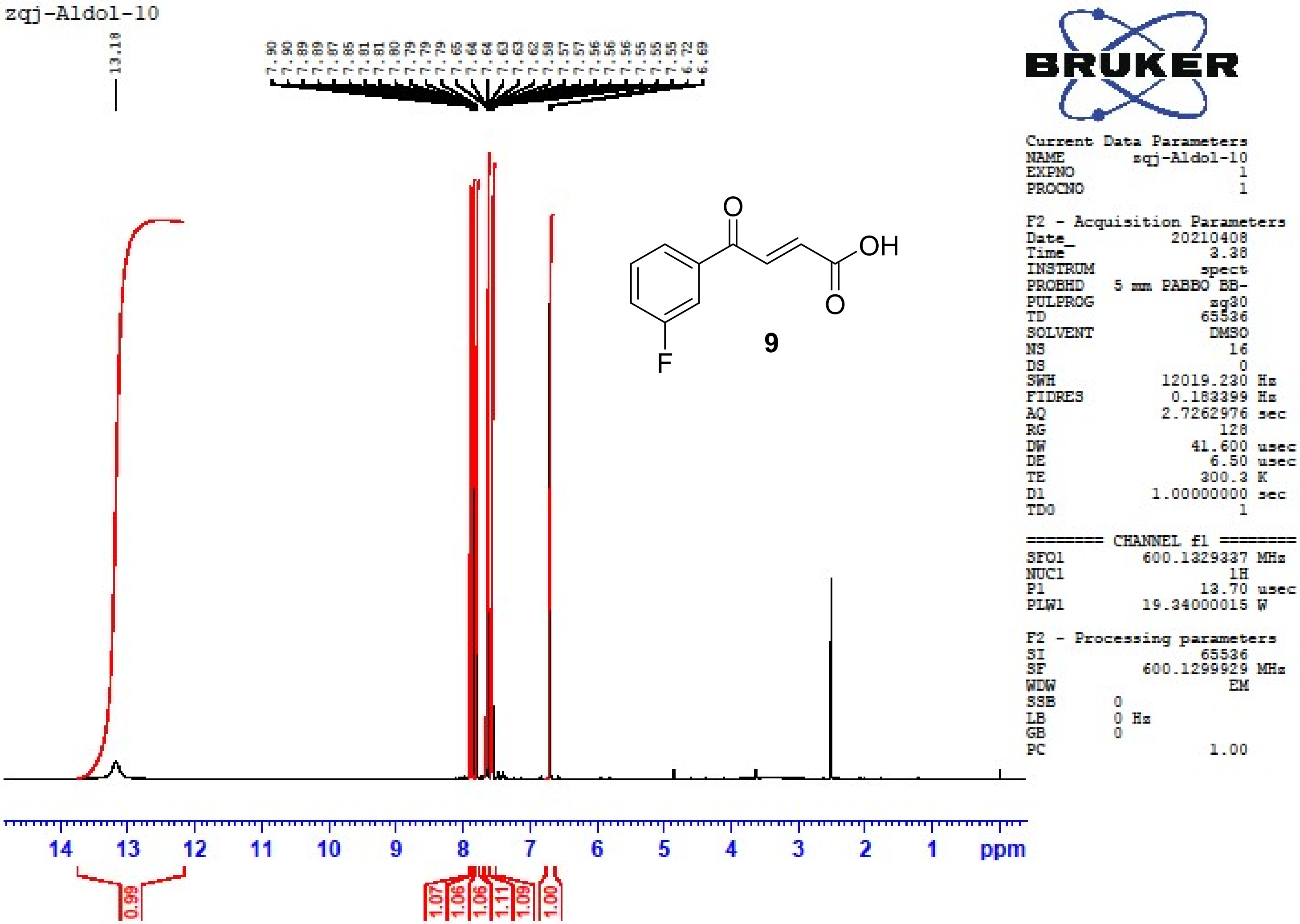
zqj-Aldol-10
13.18
7.90 7.90 7.89 7.87 7.85 7.81 7.81 7.80 7.79 7.79 7.79 7.65 7.64 7.64 7.63 7.63 7.62 7.58 7.57 7.57 7.56 7.56 7.56 7.55 7.55 7.55 6.72 6.69
O
OH
O
F
9
BRUKER
Current Data Parameters
NAME zqj-Aldol-10
EXPNO 1
PROCNO 1
F2 - Acquisition Parameters
Date_ 20210408
Time 3.38
INSTRUM spect
PROBHD 5 mm PABBO BB-
PULPROG zg30
TD 65536
SOLVENT DMSO
NS 16
DS 0
SWH 12019.230 Hz
FIDRES 0.183399 Hz
AQ 2.7262976 sec
RG 128
DW 41.600 usec
DE 6.50 usec
TE 300.3 K
D1 1.00000000 sec
TD0 1
======== CHANNEL f1 ========
SFO1 600.1329337 MHz
NUC1 1H
P1 13.70 usec
PLW1 19.34000015 W
F2 - Processing parameters
SI 65536
SF 600.1299929 MHz
WDW EM
SSB 0
LB 0 Hz
GB 0
PC 1.00
14 13 12 11 10 9 8 7 6 5 4 3 2 1 ppm
0.99
1.07 1.06 1.06 1.11 1.09 1.00

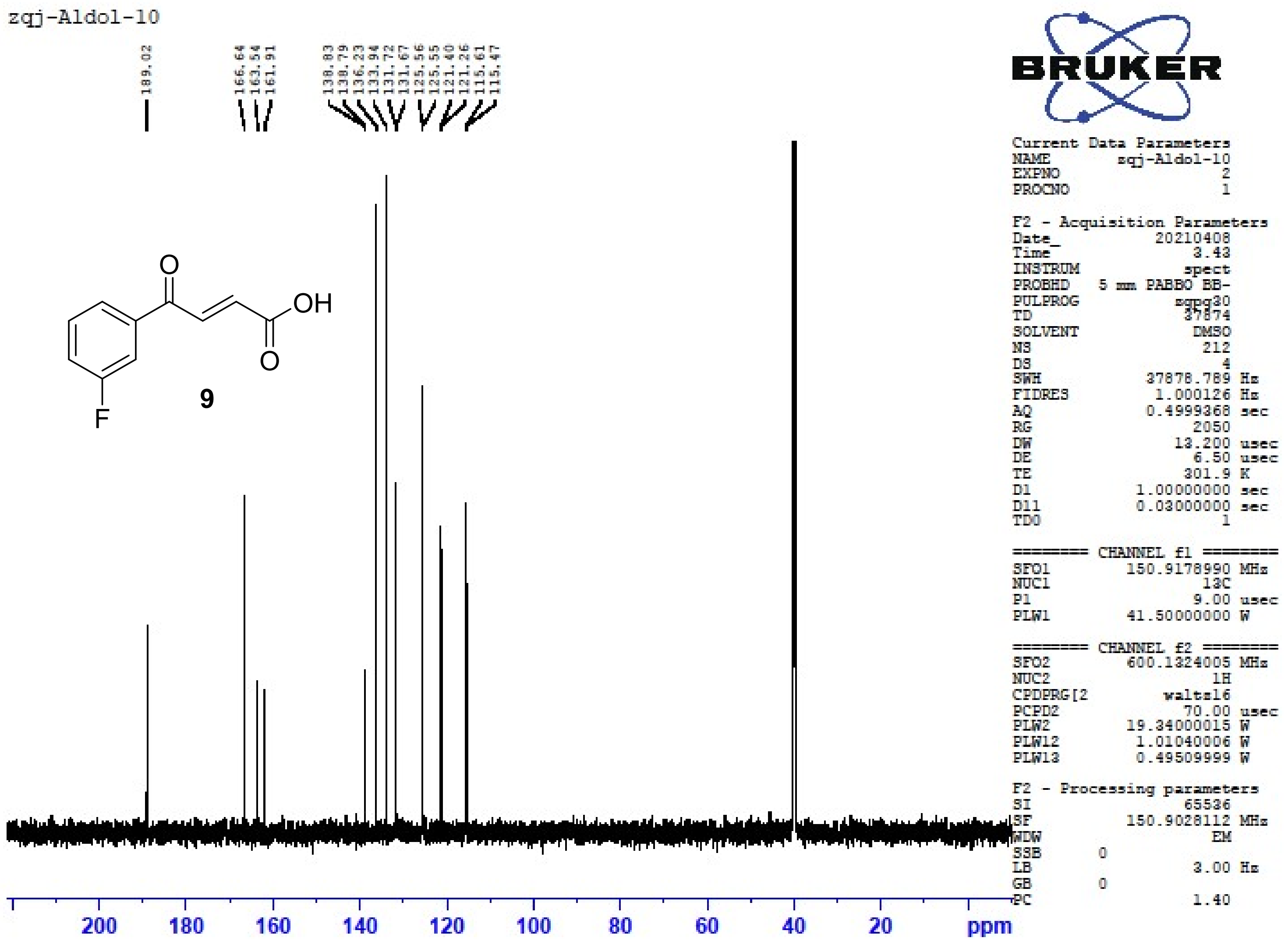
zqj-Aldol-10
189.02
166.64
163.54
161.91
138.83
138.79
136.23
133.94
131.72
131.67
125.56
125.55
121.40
121.26
115.61
115.47
O
OH
O
F
9
BRUKER
Current Data Parameters
NAME zqj-Aldol-10
EXPNO 2
PROCNO 1
F2 - Acquisition Parameters
Date_ 20210408
Time 3.43
INSTRUM spect
PROBHD 5 mm PABBO BB-
PULPROG zgpg30
TD 37874
SOLVENT DMSO
NS 212
DS 4
SWH 37878.789 Hz
FIDRES 1.000126 Hz
AQ 0.4999368 sec
RG 2050
DW 13.200 usec
DE 6.50 usec
TE 301.9 K
D1 1.00000000 sec
D11 0.03000000 sec
TD0 1
======== CHANNEL f1 ========
SFO1 150.9178990 MHz
NUC1 13C
P1 9.00 usec
PLW1 41.50000000 W
======== CHANNEL f2 ========
SFO2 600.1324005 MHz
NUC2 1H
CPDPRG[2 waltz16
PCPD2 70.00 usec
PLW2 19.34000015 W
PLW12 1.01040006 W
PLW13 0.49509999 W
F2 - Processing parameters
SI 65536
SF 150.9028112 MHz
WDW EM
SSB 0
LB 3.00 Hz
GB 0
PC 1.40
200
180
160
140
120
100
80
60
40
20
ppm

A-10-F

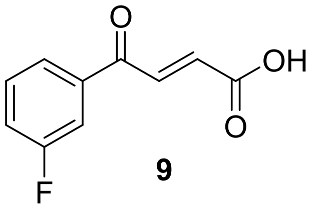

9

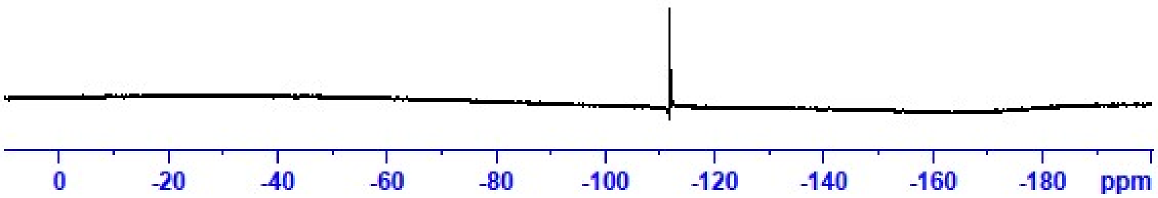


```
Current Data Parameters
NAME                A-10-F
EXPNO                    3
PROCNO                   1

F2 - Acquisition Parameters
Date_             20210506
Time                 16.51
INSTRUM              spect
PROBHD   5 mm PABBO BB-
PULPROG             zgflqn
TD                  131072
SOLVENT               DMSO
NS                      32
DS                       4
SWH              66964.289 Hz
FIDRES            0.510897 Hz
AQ               0.9786710 sec
RG                     128
DW                   7.467 usec
DE                    6.50 usec
TE                   300.0 K
D1              1.00000000 sec
TD0                      1

======== CHANNEL f1 ========
SFO1          282.3761148 MHz
NUC1                   19F
P1                   19.50 usec
PLW1           11.00000000 W

F2 - Processing parameters
SI                   65536
SF            282.4043550 MHz
WDW                     EM
SSB       0
LB                    3.00 Hz
GB        0
PC                    1.00
```

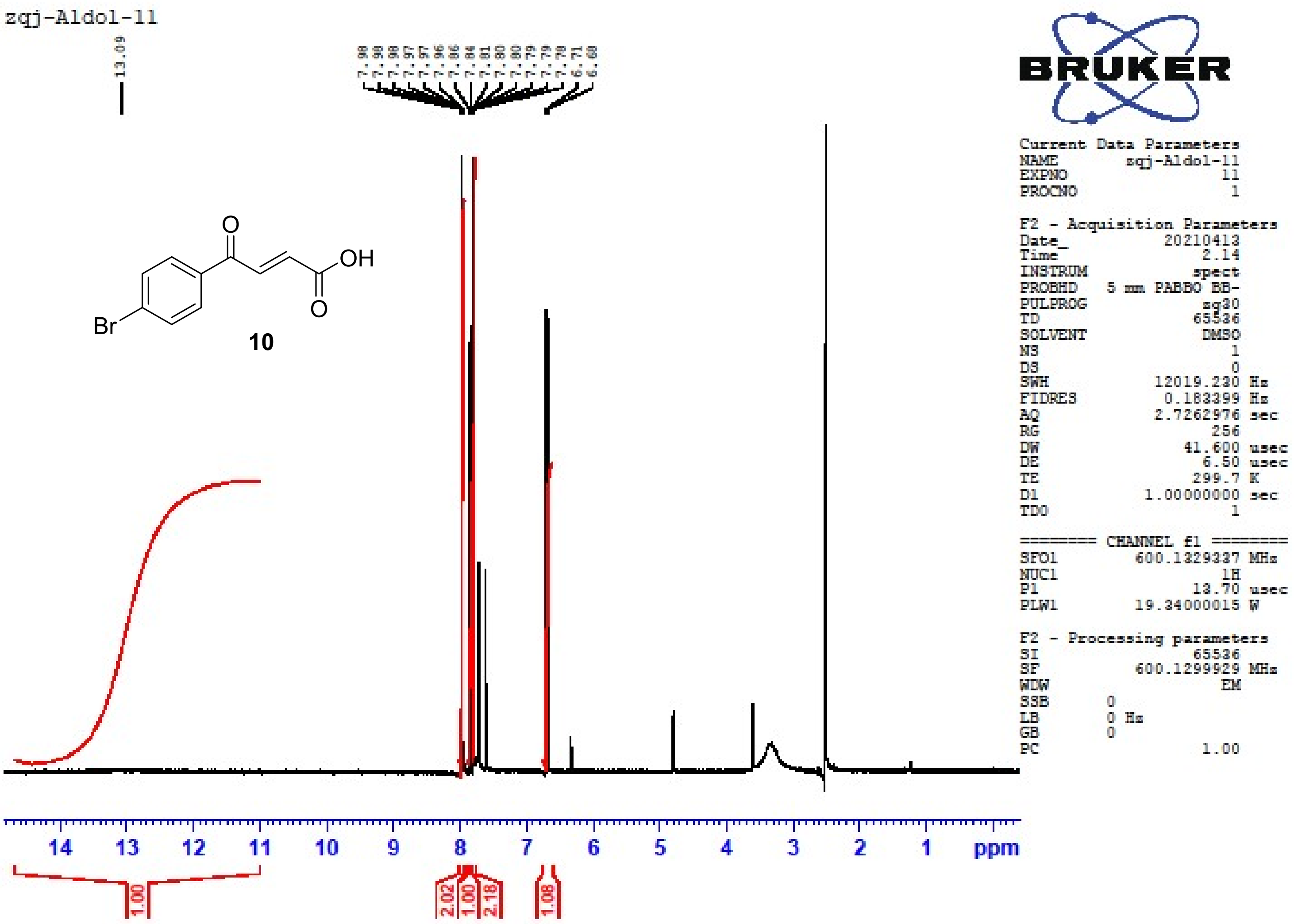
zqj-Aldol-11
13.09
7.98
7.98
7.98
7.97
7.96
7.86
7.84
7.81
7.80
7.80
7.79
7.79
7.78
6.71
O
OH
O
Br
10
BRUKER
Current Data Parameters
NAME zqj-Aldol-11
EXPNO 11
PROCNO 1
F2 - Acquisition Parameters
Date_ 20210413
Time 2.14
INSTRUM spect
PROBHD 5 mm PABBO BB-
PULPROG zg30
TD 65536
SOLVENT DMSO
NS 1
DS 0
SWH 12019.230 Hz
FIDRES 0.183399 Hz
AQ 2.7262976 sec
RG 256
DW 41.600 usec
DE 6.50 usec
TE 299.7 K
D1 1.00000000 sec
TD0 1
======== CHANNEL f1 ========
SFO1 600.1329337 MHz
NUC1 1H
P1 13.70 usec
PLW1 19.34000015 W
F2 - Processing parameters
SI 65536
SF 600.1299929 MHz
WDW EM
SSB 0
LB 0 Hz
GB 0
PC 1.00
14 13 12 11 10 9 8 7 6 5 4 3 2 1 ppm
1.00
2.02
1.00
2.18
1.08

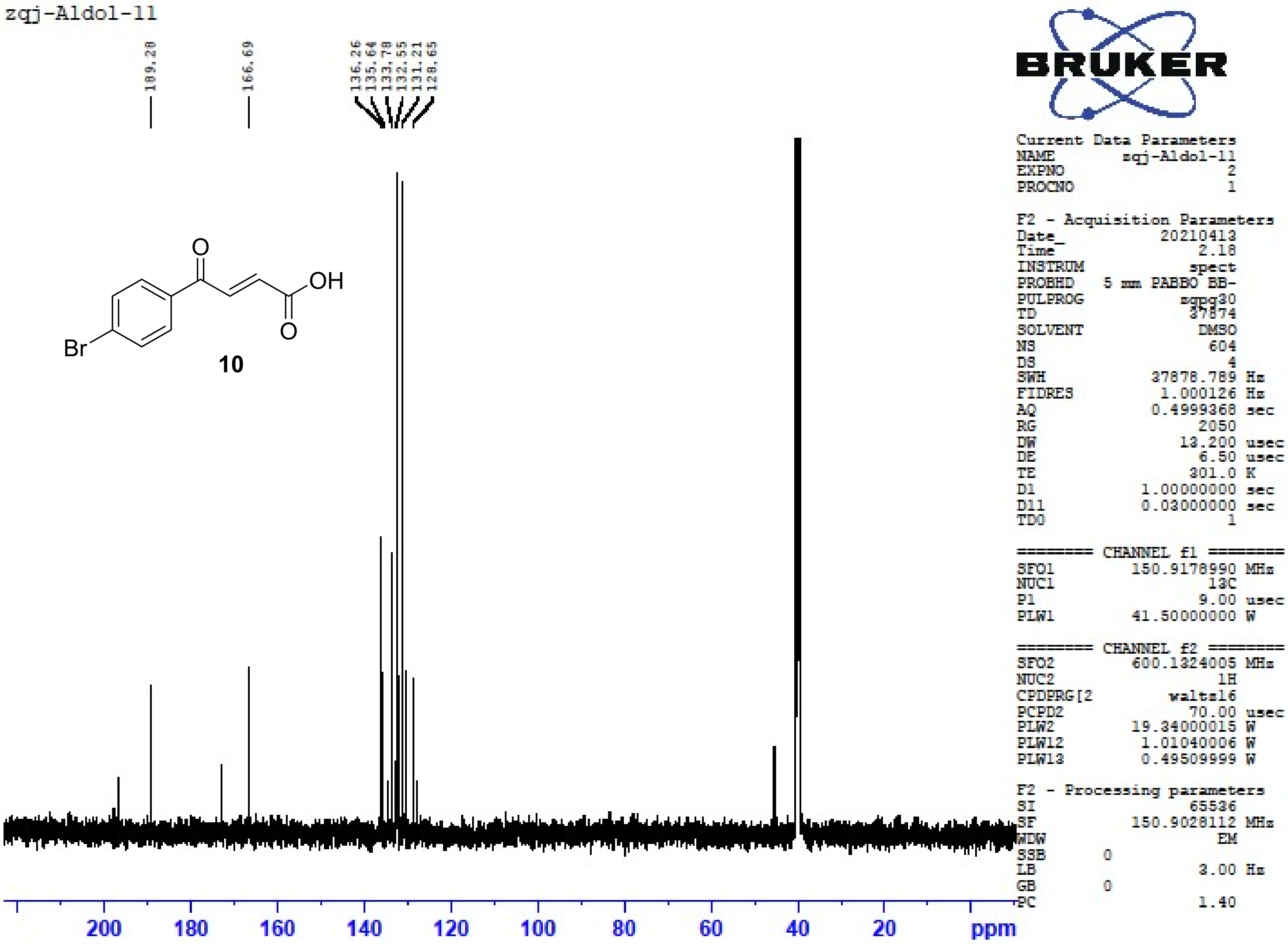
zqj-Aldol-11
189.28
166.69
136.26
135.64
133.78
132.55
131.21
128.65
OH
O
O
Br
10
BRUKER
Current Data Parameters
NAME zqj-Aldol-11
EXPNO 2
PROCNO 1
F2 - Acquisition Parameters
Date_ 20210413
Time 2.18
INSTRUM spect
PROBHD 5 mm PABBO BB-
PULPROG zgpg30
TD 37874
SOLVENT DMSO
NS 604
DS 4
SWH 37878.789 Hz
FIDRES 1.000126 Hz
AQ 0.4999368 sec
RG 2050
DW 13.200 usec
DE 6.50 usec
TE 301.0 K
D1 1.00000000 sec
D11 0.03000000 sec
TD0 1
======== CHANNEL f1 ========
SFO1 150.9178990 MHz
NUC1 13C
P1 9.00 usec
PLW1 41.50000000 W
======== CHANNEL f2 ========
SFO2 600.1324005 MHz
NUC2 1H
CPDPRG[2 waltz16
PCPD2 70.00 usec
PLW2 19.34000015 W
PLW12 1.01040006 W
PLW13 0.49509999 W
F2 - Processing parameters
SI 65536
SF 150.9028112 MHz
WDW EM
SSB 0
LB 3.00 Hz
GB 0
PC 1.40
200
180
160
140
120
100
80
60
40
20
ppm

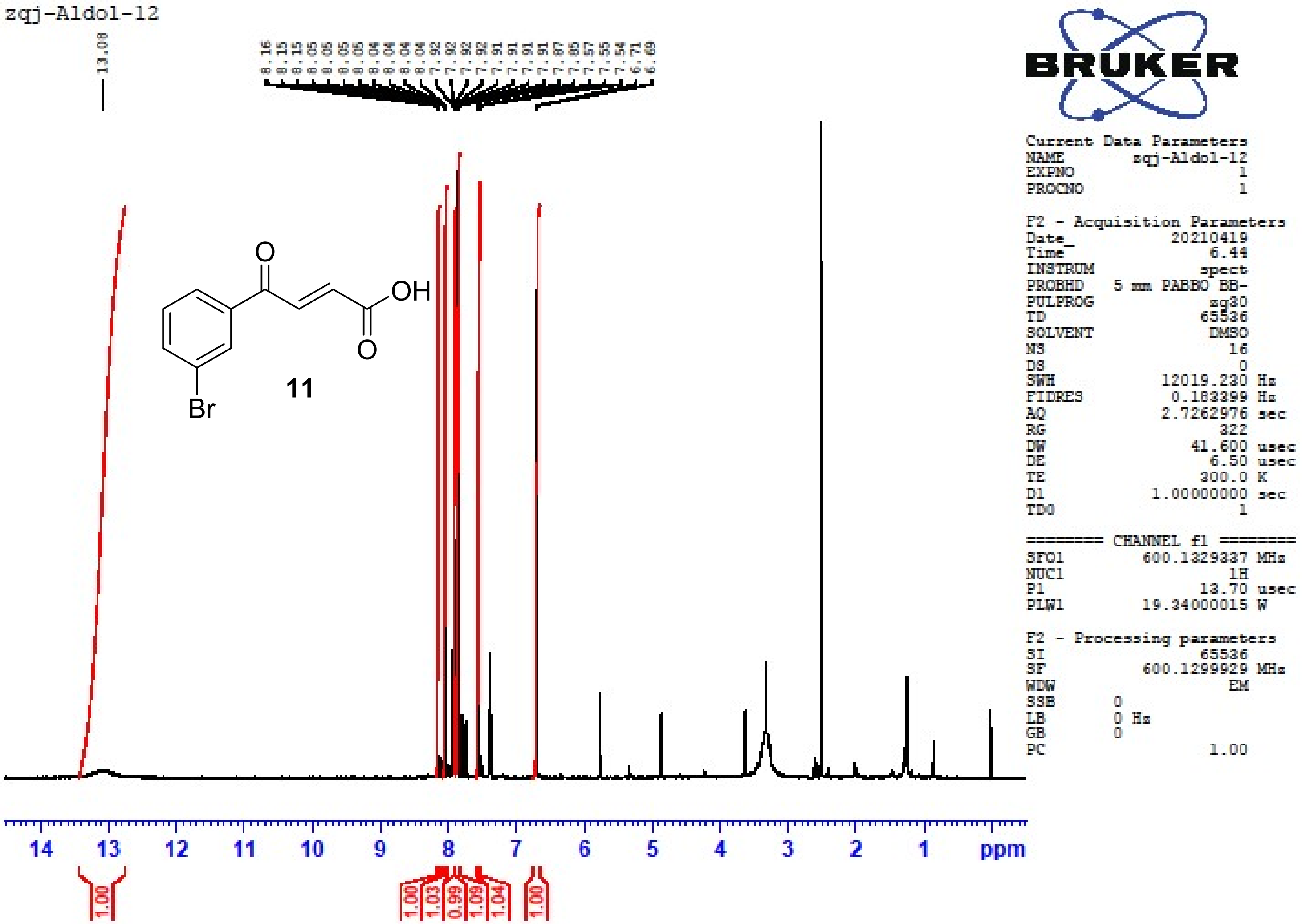
zqj-Aldol-12
13.08
8.16 8.15 8.15 8.05 8.05 8.05 8.04 8.04 8.04 8.04 7.92 7.92 7.92 7.91 7.91 7.91 7.91 7.87 7.85 7.57 7.55 7.54 6.71 6.69
OH
O
O
Br
11
14 13 12 11 10 9 8 7 6 5 4 3 2 1 ppm
1.00 1.00 1.03 0.99 1.09 1.04 1.00
BRUKER
Current Data Parameters
NAME zqj-Aldol-12
EXPNO 1
PROCNO 1
F2 - Acquisition Parameters
Date_ 20210419
Time 6.44
INSTRUM spect
PROBHD 5 mm PABBO BB-
PULPROG zg30
TD 65536
SOLVENT DMSO
NS 16
DS 0
SWH 12019.230 Hz
FIDRES 0.183399 Hz
AQ 2.7262976 sec
RG 322
DW 41.600 usec
DE 6.50 usec
TE 300.0 K
D1 1.00000000 sec
TD0 1
======== CHANNEL f1 ========
SFO1 600.1329337 MHz
NUC1 1H
P1 13.70 usec
PLW1 19.34000015 W
F2 - Processing parameters
SI 65536
SF 600.1299929 MHz
WDW EM
SSB 0
LB 0 Hz
GB 0
PC 1.00

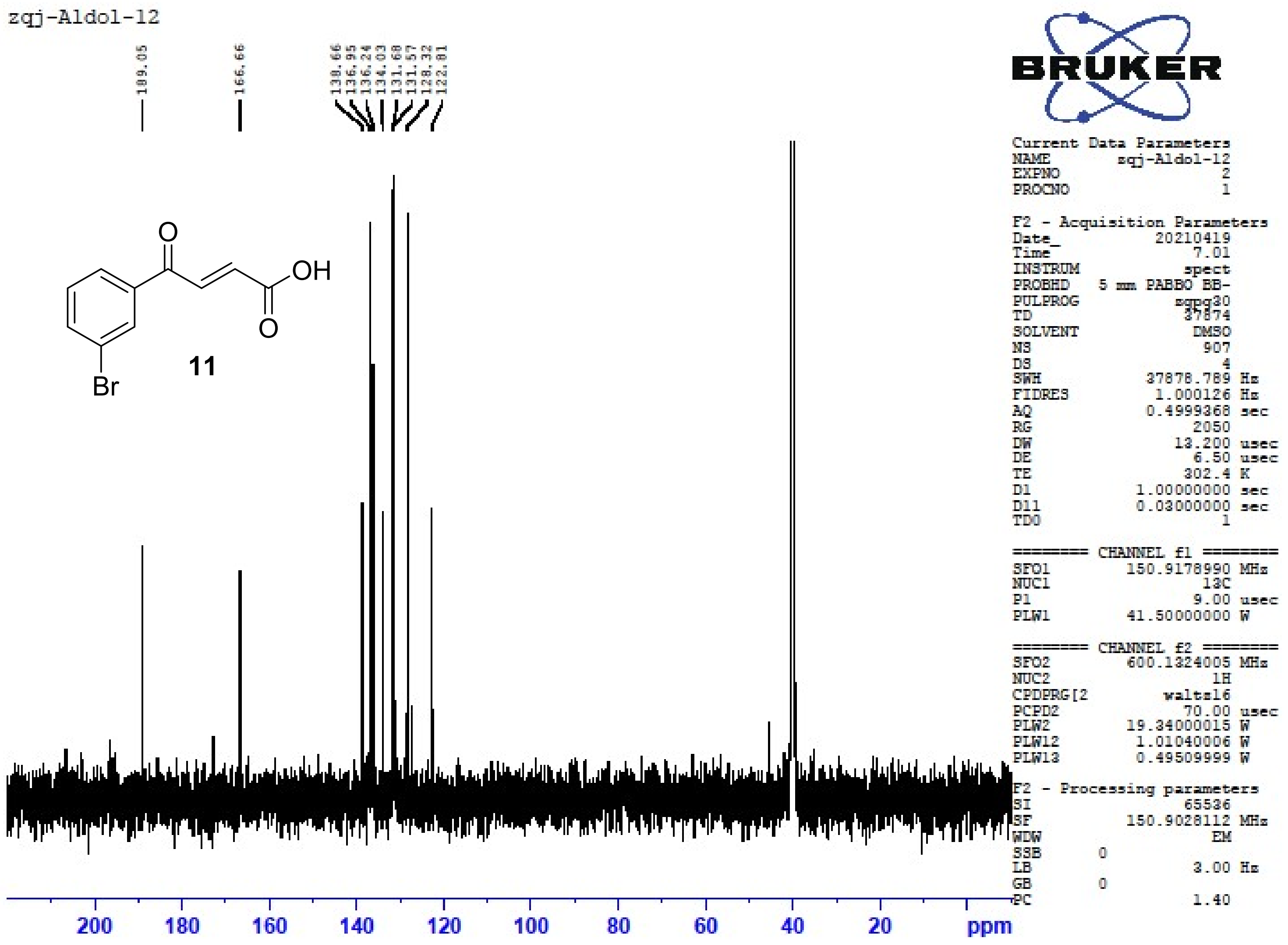
zqj-Aldol-12
189.05
166.66
138.66
136.95
136.24
134.03
131.68
131.57
128.32
122.81
O
OH
O
Br
11
200 180 160 140 120 100 80 60 40 20 ppm
BRUKER
Current Data Parameters
NAME zqj-Aldol-12
EXPNO 2
PROCNO 1
F2 - Acquisition Parameters
Date_ 20210419
Time 7.01
INSTRUM spect
PROBHD 5 mm PABBO BB-
PULPROG zgpg30
TD 37874
SOLVENT DMSO
NS 907
DS 4
SWH 37878.789 Hz
FIDRES 1.000126 Hz
AQ 0.4999368 sec
RG 2050
DW 13.200 usec
DE 6.50 usec
TE 302.4 K
D1 1.00000000 sec
D11 0.03000000 sec
TD0 1
======== CHANNEL f1 ========
SFO1 150.9178990 MHz
NUC1 13C
P1 9.00 usec
PLW1 41.50000000 W
======== CHANNEL f2 ========
SFO2 600.1324005 MHz
NUC2 1H
CPDPRG[2 waltz16
PCPD2 70.00 usec
PLW2 19.34000015 W
PLW12 1.01040006 W
PLW13 0.49509999 W
F2 - Processing parameters
SI 65536
SF 150.9028112 MHz
WDW EM
SSB 0
LB 3.00 Hz
GB 0
PC 1.40

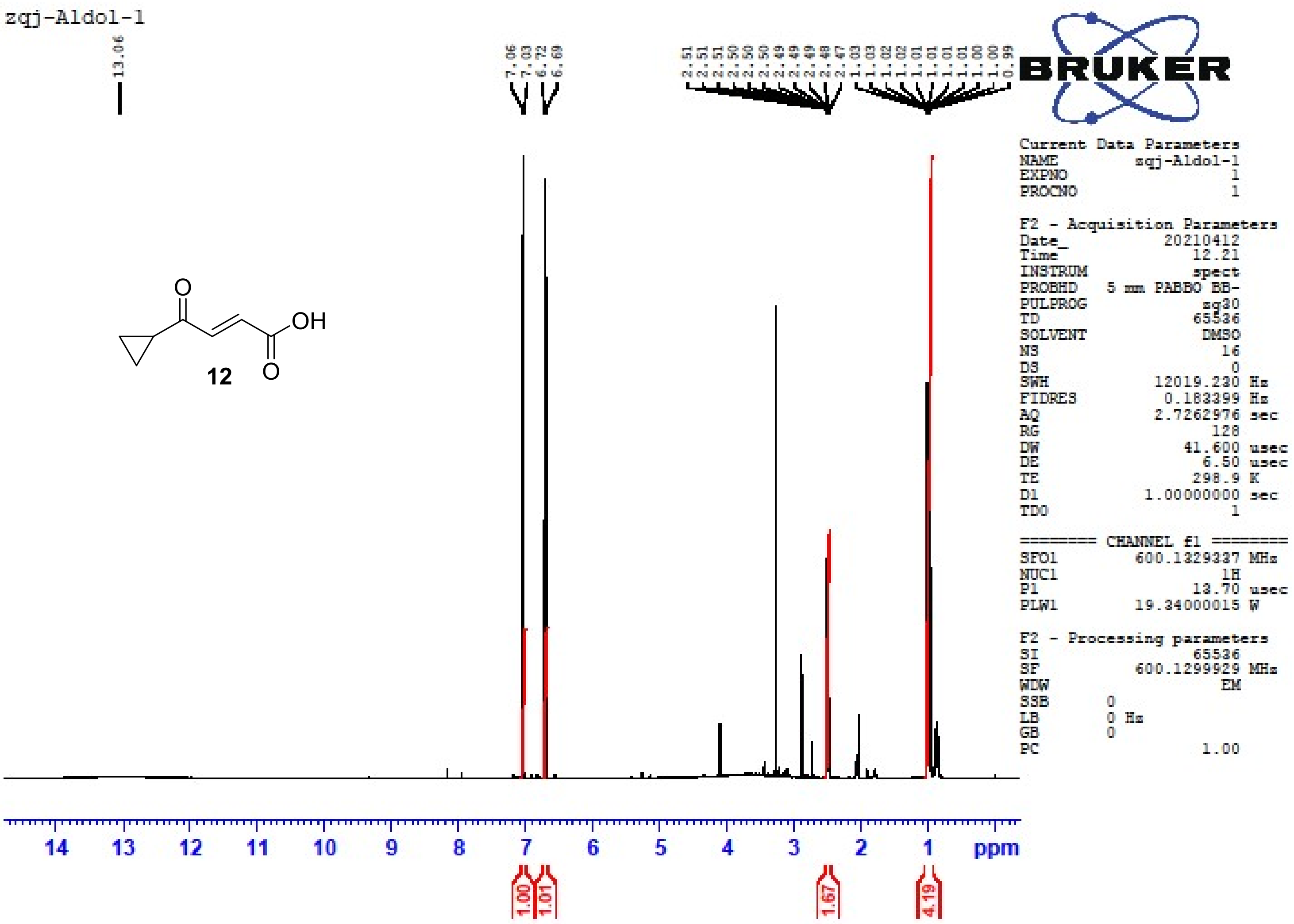
zqj-Aldol-1
13.06
7.06
7.03
6.72
6.69
2.51
2.51
2.51
2.50
2.50
2.50
2.49
2.49
2.49
2.48
2.47
1.03
1.03
1.02
1.02
1.01
1.01
1.01
1.01
1.00
1.00
0.99
BRUKER
O
OH
O
12
Current Data Parameters
NAME zqj-Aldol-1
EXPNO 1
PROCNO 1
F2 - Acquisition Parameters
Date_ 20210412
Time 12.21
INSTRUM spect
PROBHD 5 mm PABBO BB-
PULPROG zg30
TD 65536
SOLVENT DMSO
NS 16
DS 0
SWH 12019.230 Hz
FIDRES 0.183399 Hz
AQ 2.7262976 sec
RG 128
DW 41.600 usec
DE 6.50 usec
TE 298.9 K
D1 1.00000000 sec
TD0 1
======== CHANNEL f1 ========
SFO1 600.1329337 MHz
NUC1 1H
P1 13.70 usec
PLW1 19.34000015 W
F2 - Processing parameters
SI 65536
SF 600.1299929 MHz
WDW EM
SSB 0
LB 0 Hz
GB 0
PC 1.00
14 13 12 11 10 9 8 7 6 5 4 3 2 1 ppm
1.00
1.01
1.67
4.19

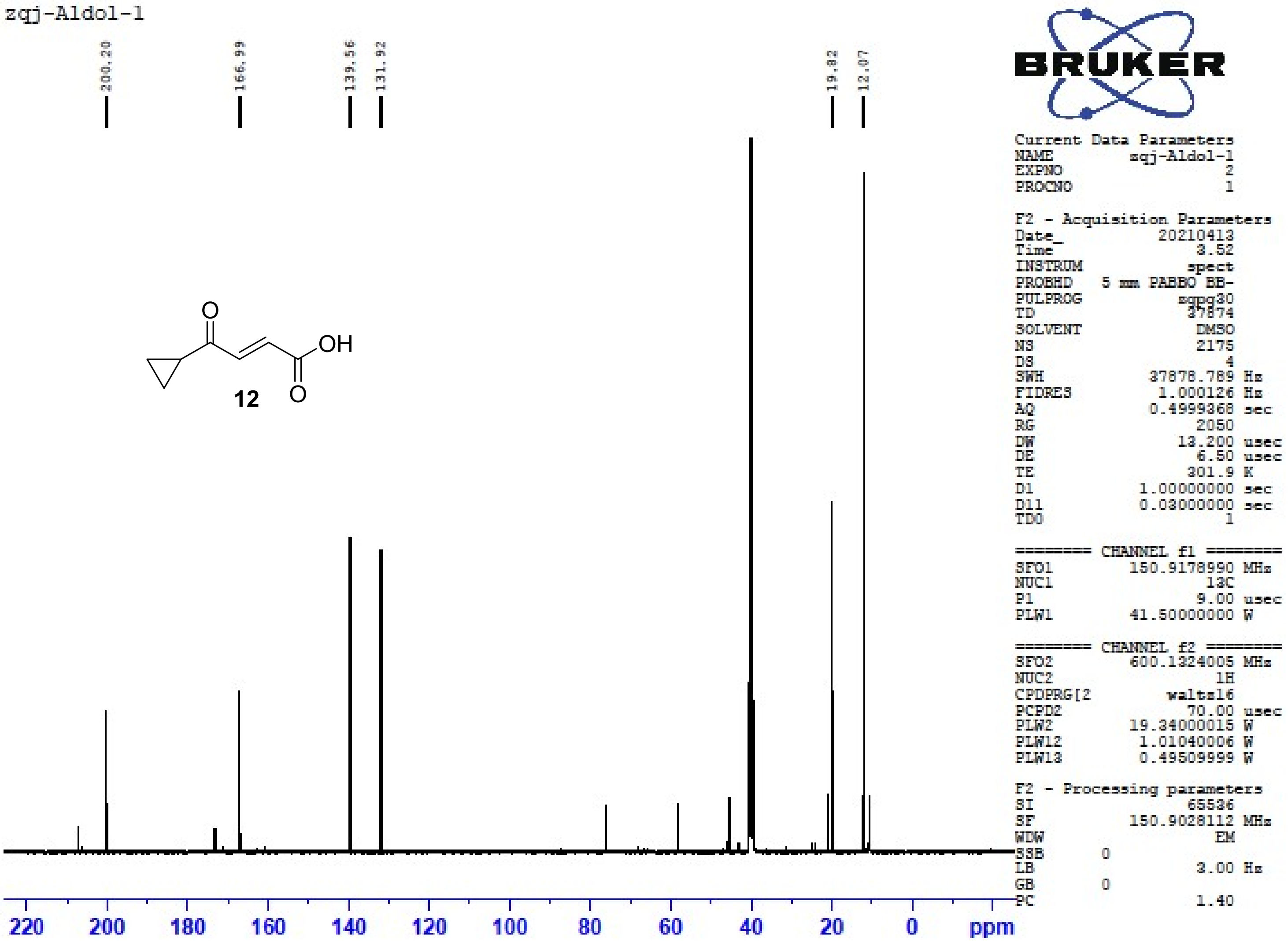
zqj-Aldol-1
200.20
166.99
139.56
131.92
19.82
12.07
O
OH
O
12
220 200 180 160 140 120 100 80 60 40 20 0 ppm
BRUKER
Current Data Parameters
NAME zqj-Aldol-1
EXPNO 2
PROCNO 1
F2 - Acquisition Parameters
Date_ 20210413
Time 3.52
INSTRUM spect
PROBHD 5 mm PABBO BB-
PULPROG zgpg30
TD 37874
SOLVENT DMSO
NS 2175
DS 4
SWH 37878.789 Hz
FIDRES 1.000126 Hz
AQ 0.4999368 sec
RG 2050
DW 13.200 usec
DE 6.50 usec
TE 301.9 K
D1 1.00000000 sec
D11 0.03000000 sec
TD0 1
======== CHANNEL f1 ========
SFO1 150.9178990 MHz
NUC1 13C
P1 9.00 usec
PLW1 41.50000000 W
======== CHANNEL f2 ========
SFO2 600.1324005 MHz
NUC2 1H
CPDPRG[2 waltz16
PCPD2 70.00 usec
PLW2 19.34000015 W
PLW12 1.01040006 W
PLW13 0.49509999 W
F2 - Processing parameters
SI 65536
SF 150.9028112 MHz
WDW EM
SSB 0
LB 3.00 Hz
GB 0
PC 1.40

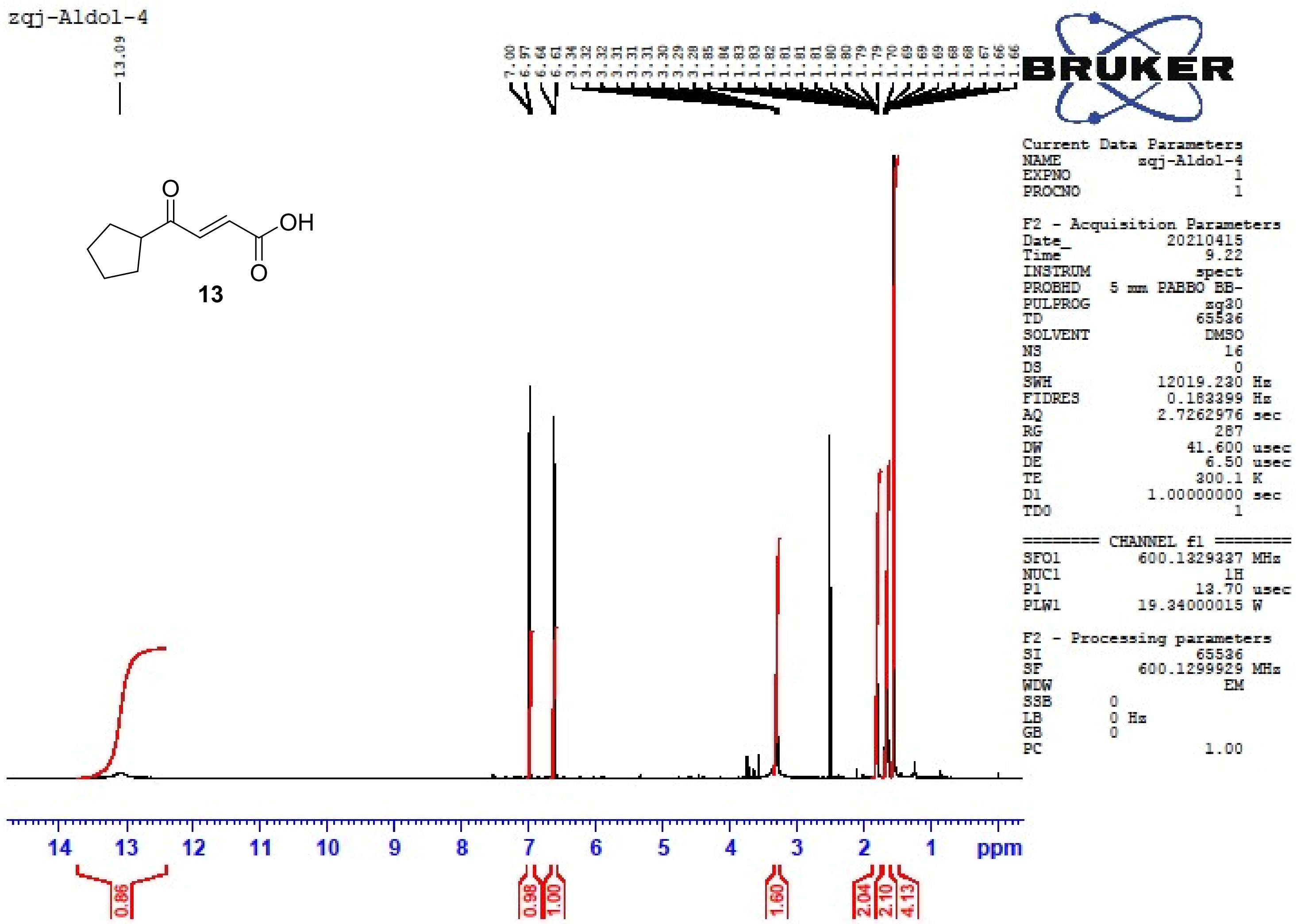
zqj-Aldol-4
13.09
7.00 6.97 6.64 6.61 3.34 3.32 3.32 3.31 3.31 3.30 3.29 3.28 1.85 1.84 1.83 1.82 1.81 1.81 1.81 1.80 1.80 1.79 1.79 1.70 1.69 1.69 1.69 1.68 1.68 1.67 1.66 1.66
OH
O
O
13
BRUKER
Current Data Parameters
NAME zqj-Aldol-4
EXPNO 1
PROCNO 1
F2 - Acquisition Parameters
Date_ 20210415
Time 9.22
INSTRUM spect
PROBHD 5 mm PABBO BB-
PULPROG zg30
TD 65536
SOLVENT DMSO
NS 16
DS 0
SWH 12019.230 Hz
FIDRES 0.183399 Hz
AQ 2.7262976 sec
RG 287
DW 41.600 usec
DE 6.50 usec
TE 300.1 K
D1 1.00000000 sec
TD0 1
======== CHANNEL f1 ========
SFO1 600.1329337 MHz
NUC1 1H
P1 13.70 usec
PLW1 19.34000015 W
F2 - Processing parameters
SI 65536
SF 600.1299929 MHz
WDW EM
SSB 0
LB 0 Hz
GB 0
PC 1.00
14 13 12 11 10 9 8 7 6 5 4 3 2 1 ppm
0.86 0.98 1.00 1.60 2.04 2.10 4.13

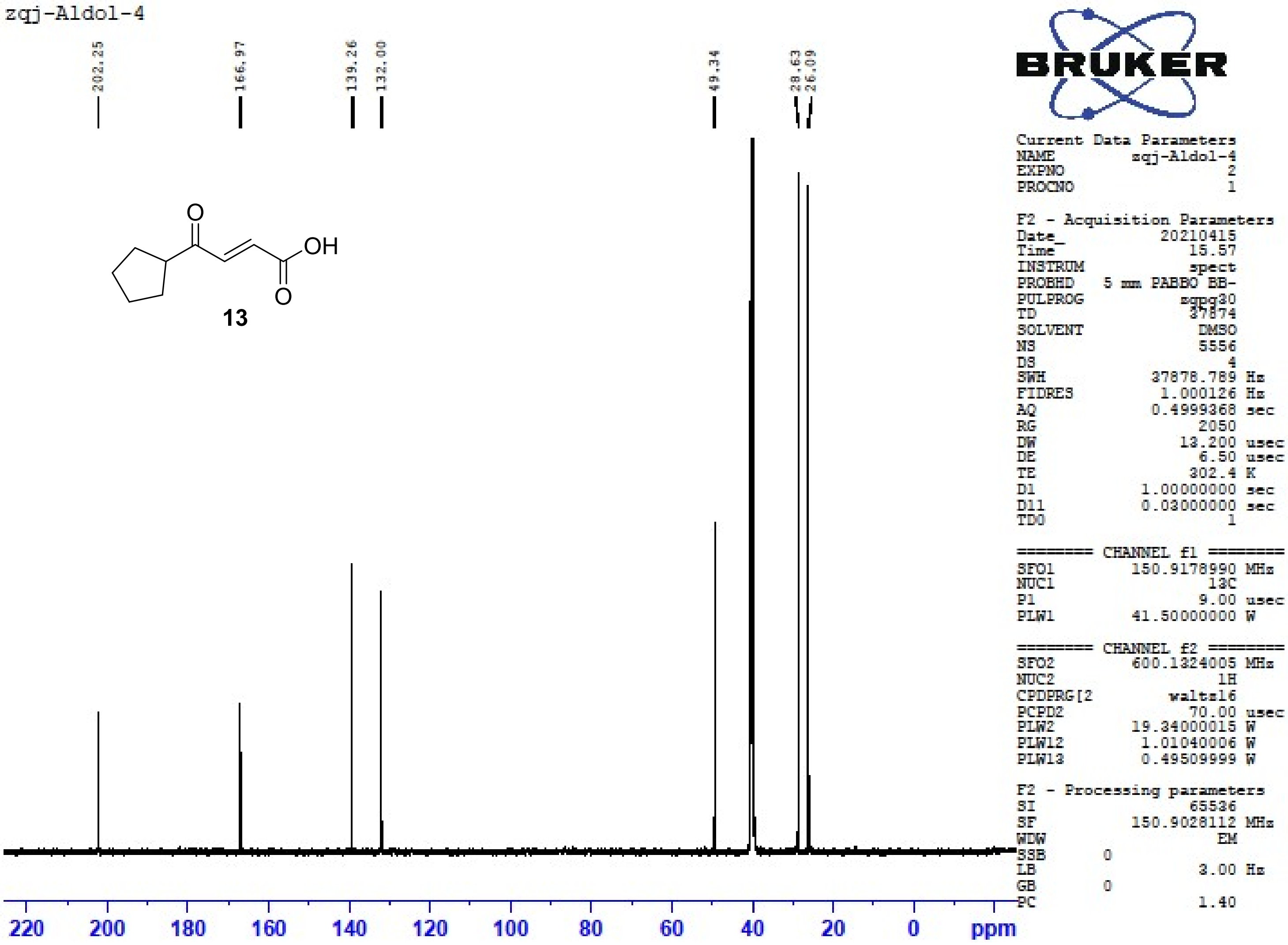
zqj-Aldol-4
202.25
166.97
139.26
132.00
49.34
28.63
26.09
OH
O
O
13
BRUKER
Current Data Parameters
NAME zqj-Aldol-4
EXPNO 2
PROCNO 1
F2 - Acquisition Parameters
Date_ 20210415
Time 15.57
INSTRUM spect
PROBHD 5 mm PABBO BB-
PULPROG zgpg30
TD 37874
SOLVENT DMSO
NS 5556
DS 4
SWH 37878.789 Hz
FIDRES 1.000126 Hz
AQ 0.4999368 sec
RG 2050
DW 13.200 usec
DE 6.50 usec
TE 302.4 K
D1 1.00000000 sec
D11 0.03000000 sec
TD0 1
======== CHANNEL f1 ========
SFO1 150.9178990 MHz
NUC1 13C
P1 9.00 usec
PLW1 41.50000000 W
======== CHANNEL f2 ========
SFO2 600.1324005 MHz
NUC2 1H
CPDPRG[2 waltz16
PCPD2 70.00 usec
PLW2 19.34000015 W
PLW12 1.01040006 W
PLW13 0.49509999 W
F2 - Processing parameters
SI 65536
SF 150.9028112 MHz
WDW EM
SSB 0
LB 3.00 Hz
GB 0
PC 1.40
220
200
180
160
140
120
100
80
60
40
20
0
ppm

Compound 6

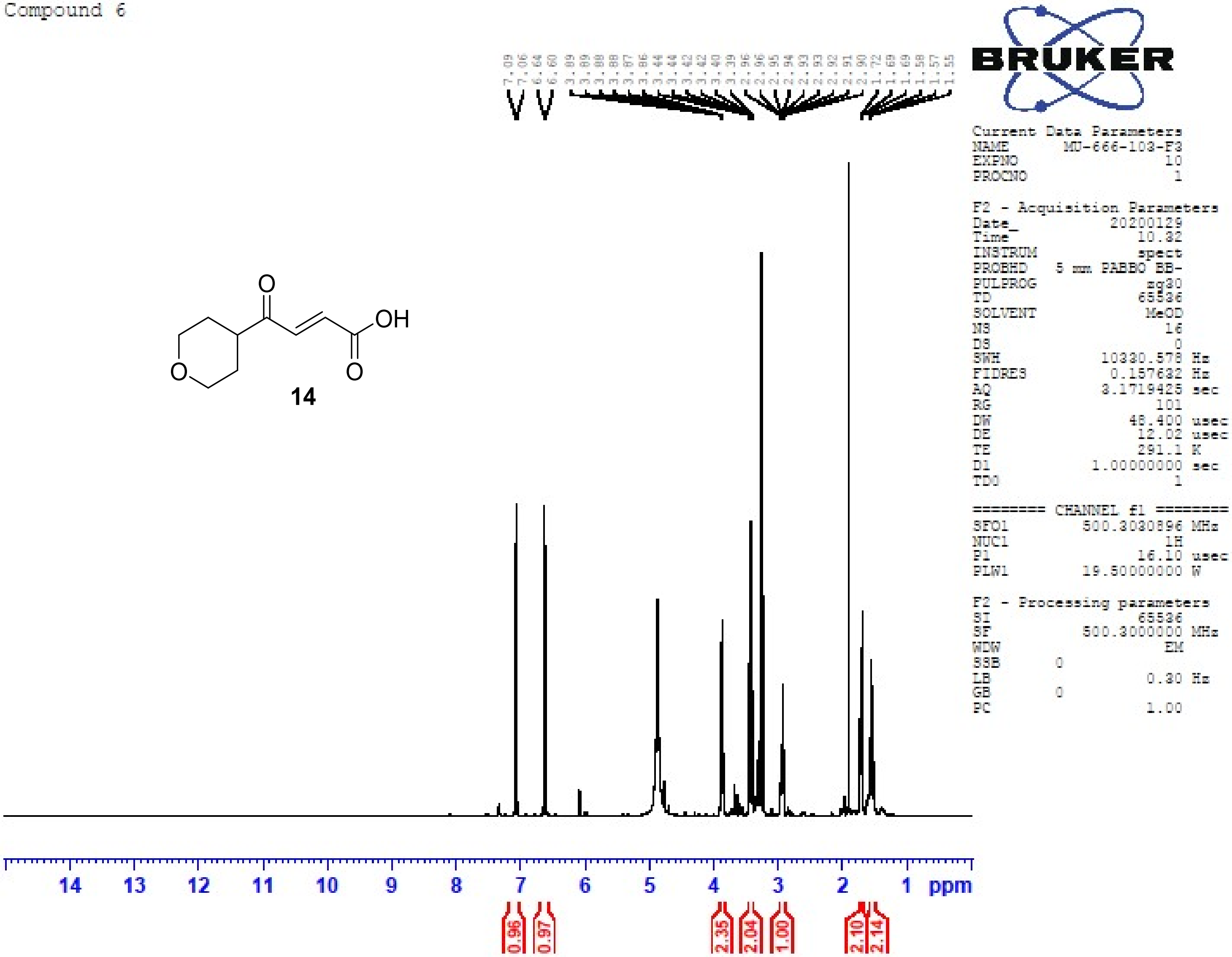
OH
O
O
O
14
7.09
7.06
6.64
6.60
3.89
3.89
3.88
3.87
3.86
3.44
3.44
3.42
3.42
3.40
3.39
2.96
2.96
2.95
2.94
2.93
2.93
2.92
2.91
2.90
1.72
1.69
1.69
1.58
1.57
1.55
Current Data Parameters
NAME MU-666-103-F3
EXPNO 10
PROCNO 1
F2 - Acquisition Parameters
Date_ 20200129
Time 10.32
INSTRUM spect
PROBHD 5 mm PABBO BB-
PULPROG zg30
TD 65536
SOLVENT MeOD
NS 16
DS 0
SWH 10330.578 Hz
FIDRES 0.157632 Hz
AQ 3.1719425 sec
RG 101
DW 48.400 usec
DE 12.02 usec
TE 291.1 K
D1 1.00000000 sec
TD0 1
======== CHANNEL f1 ========
SFO1 500.3030896 MHz
NUC1 1H
P1 16.10 usec
PLW1 19.50000000 W
F2 - Processing parameters
SI 65536
SF 500.3000000 MHz
WDW EM
SSB 0
LB 0.30 Hz
GB 0
PC 1.00
14 13 12 11 10 9 8 7 6 5 4 3 2 1 ppm
0.96
0.97
2.35
2.04
1.00
2.10
2.14

Compound 6

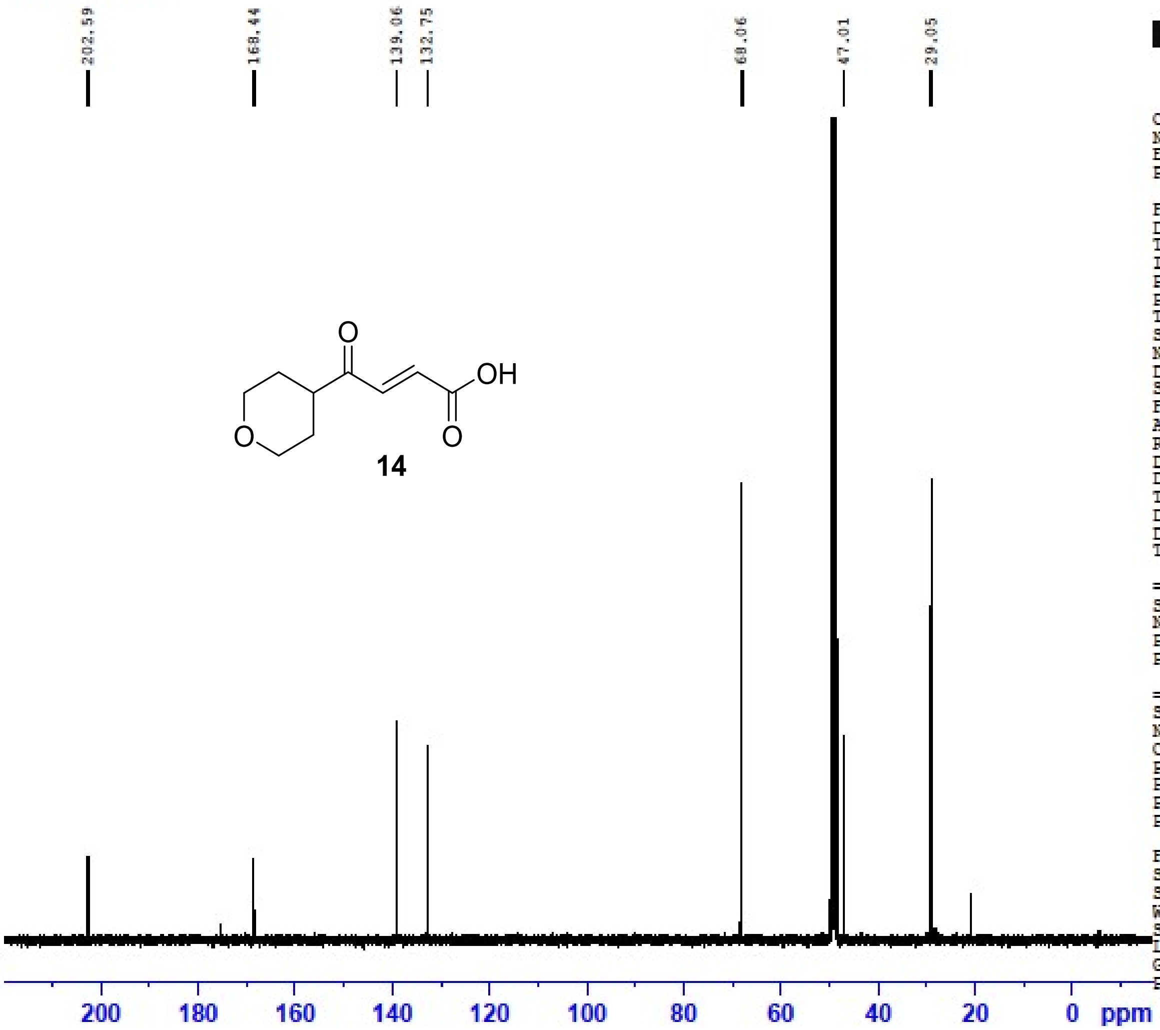

BRUKER

Current Data Parameters
NAME MU-666-103-F3
EXPNO 11
PROCNO 1

F2 - Acquisition Parameters
Date_ 20200129
Time 14.45
INSTRUM spect
PROBHD 5 mm PABBO BB-
PULPROG zgpg30
TD 65536
SOLVENT MeOD
NS 256
DS 2
SWH 29761.904 Hz
FIDRES 0.454131 Hz
AQ 1.1010048 sec
RG 456
DW 16.800 usec
DE 7.65 usec
TE 294.3 K
D1 2.00000000 sec
D11 0.03000000 sec
TD0 1

======== CHANNEL f1 ========
SFO1 125.8131151 MHz
NUC1 13C
P1 9.90 usec
PLW1 82.38999939 W

======== CHANNEL f2 ========
SFO2 500.3020012 MHz
NUC2 1H
CPDPRG[2 waltz16
PCPD2 80.00 usec
PLW2 19.50000000 W
PLW12 0.78978002 W
PLW13 0.39725000 W

F2 - Processing parameters
SI 65536
SF 125.8005350 MHz
WDW EM
SSB 0
LB 1.00 Hz
GB 0
PC 1.40

Compound 8

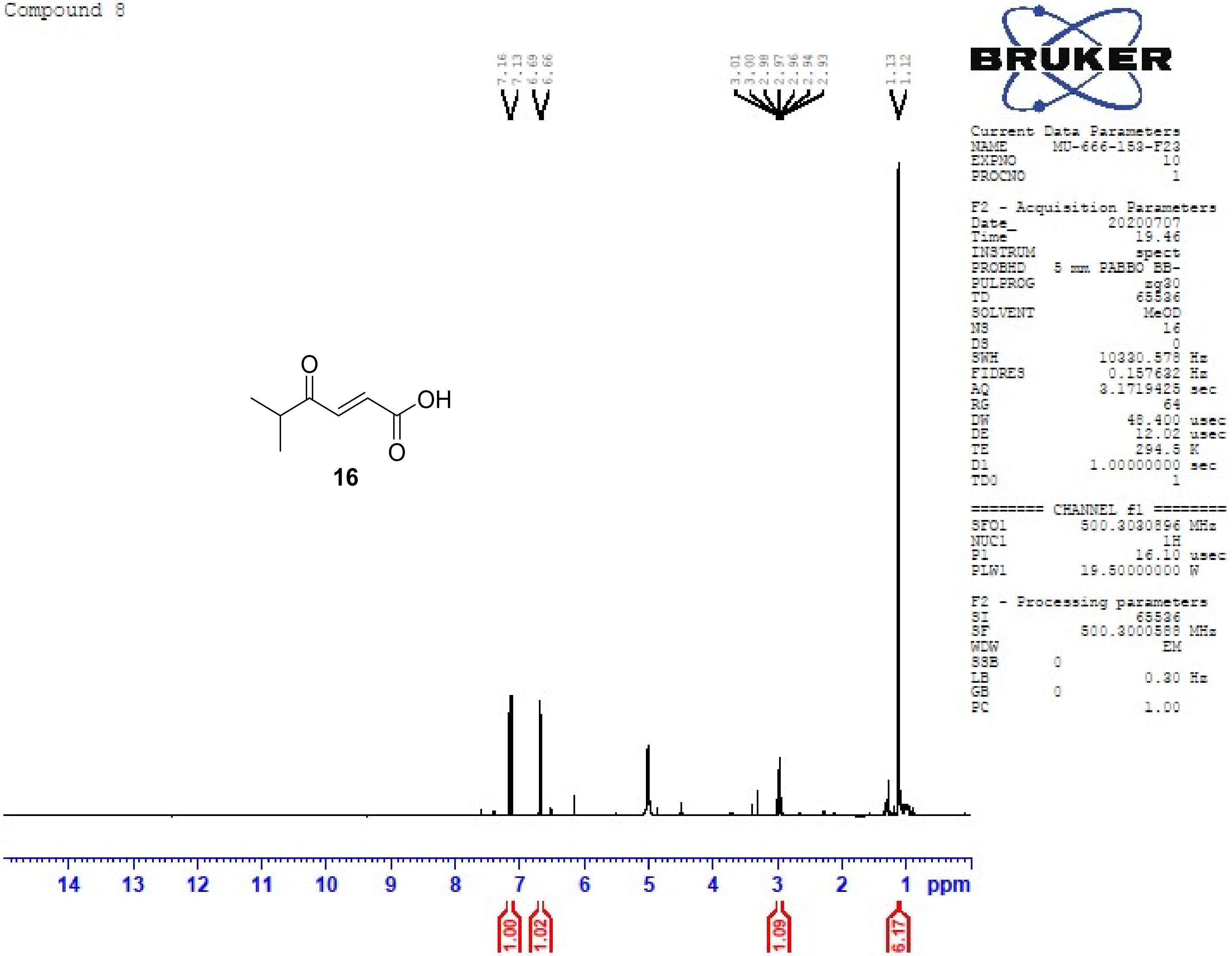
7.16
7.13
6.69
6.66
3.01
3.00
2.98
2.97
2.96
2.94
2.93
1.13
1.12
O
OH
O
16
BRUKER
Current Data Parameters
NAME MU-666-153-F23
EXPNO 10
PROCNO 1
F2 - Acquisition Parameters
Date_ 20200707
Time 19.46
INSTRUM spect
PROBHD 5 mm PABBO BB-
PULPROG zg30
TD 65536
SOLVENT MeOD
NS 16
DS 0
SWH 10330.578 Hz
FIDRES 0.157632 Hz
AQ 3.1719425 sec
RG 64
DW 48.400 usec
DE 12.02 usec
TE 294.5 K
D1 1.00000000 sec
TD0 1
======== CHANNEL f1 ========
SFO1 500.3030896 MHz
NUC1 1H
P1 16.10 usec
PLW1 19.50000000 W
F2 - Processing parameters
SI 65536
SF 500.3000588 MHz
WDW EM
SSB 0
LB 0.30 Hz
GB 0
PC 1.00
14 13 12 11 10 9 8 7 6 5 4 3 2 1 ppm
1.00
1.02
1.09
6.17

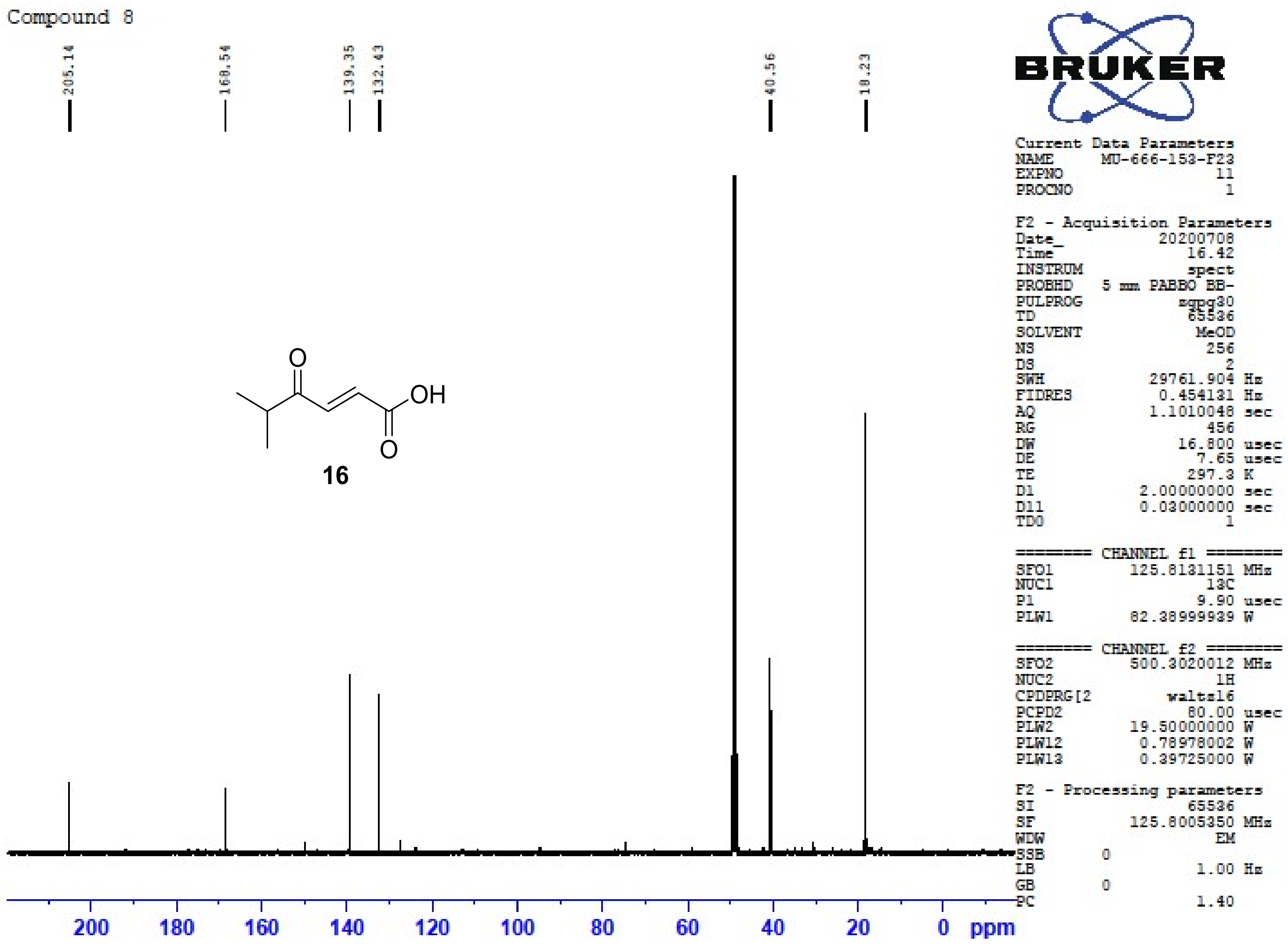
Compound 8
205.14
168.54
139.35
132.43
40.56
18.23
O
OH
O
16
BRUKER
Current Data Parameters
NAME MU-666-153-F23
EXPNO 11
PROCNO 1
F2 - Acquisition Parameters
Date_ 20200708
Time 16.42
INSTRUM spect
PROBHD 5 mm PABBO BB-
PULPROG zgpg30
TD 65536
SOLVENT MeOD
NS 256
DS 2
SWH 29761.904 Hz
FIDRES 0.454131 Hz
AQ 1.1010048 sec
RG 456
DW 16.800 usec
DE 7.65 usec
TE 297.3 K
D1 2.00000000 sec
D11 0.03000000 sec
TD0 1
======== CHANNEL f1 ========
SFO1 125.8131151 MHz
NUC1 13C
P1 9.90 usec
PLW1 82.38999939 W
======== CHANNEL f2 ========
SFO2 500.3020012 MHz
NUC2 1H
CPDPRG[2 waltz16
PCPD2 80.00 usec
PLW2 19.50000000 W
PLW12 0.78978002 W
PLW13 0.39725000 W
F2 - Processing parameters
SI 65536
SF 125.8005350 MHz
WDW EM
SSB 0
LB 1.00 Hz
GB 0
PC 1.40
200 180 160 140 120 100 80 60 40 20 0 ppm

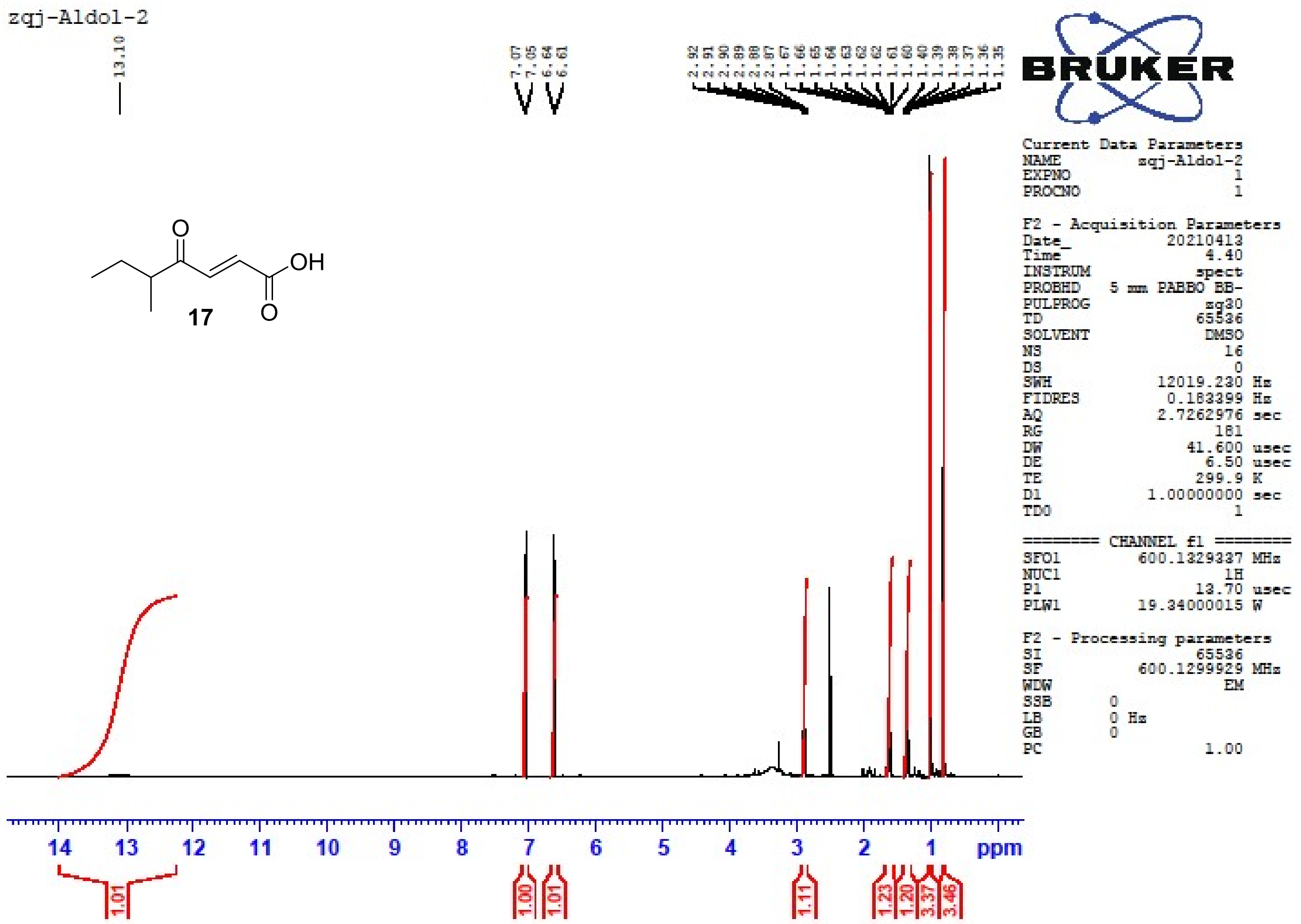
zqj-Aldol-2
13.10
7.07
7.05
6.64
6.61
2.92
2.91
2.90
2.89
2.88
2.87
1.67
1.66
1.65
1.64
1.63
1.62
1.62
1.61
1.60
1.40
1.39
1.38
1.37
1.36
1.35
O
OH
O
17
BRUKER
Current Data Parameters
NAME zqj-Aldol-2
EXPNO 1
PROCNO 1
F2 - Acquisition Parameters
Date_ 20210413
Time 4.40
INSTRUM spect
PROBHD 5 mm PABBO BB-
PULPROG zg30
TD 65536
SOLVENT DMSO
NS 16
DS 0
SWH 12019.230 Hz
FIDRES 0.183399 Hz
AQ 2.7262976 sec
RG 181
DW 41.600 usec
DE 6.50 usec
TE 299.9 K
D1 1.00000000 sec
TD0 1
======== CHANNEL f1 ========
SFO1 600.1329337 MHz
NUC1 1H
P1 13.70 usec
PLW1 19.34000015 W
F2 - Processing parameters
SI 65536
SF 600.1299929 MHz
WDW EM
SSB 0
LB 0 Hz
GB 0
PC 1.00
14 13 12 11 10 9 8 7 6 5 4 3 2 1 ppm
1.01
1.00
1.01
1.11
1.23
1.20
3.37
3.46

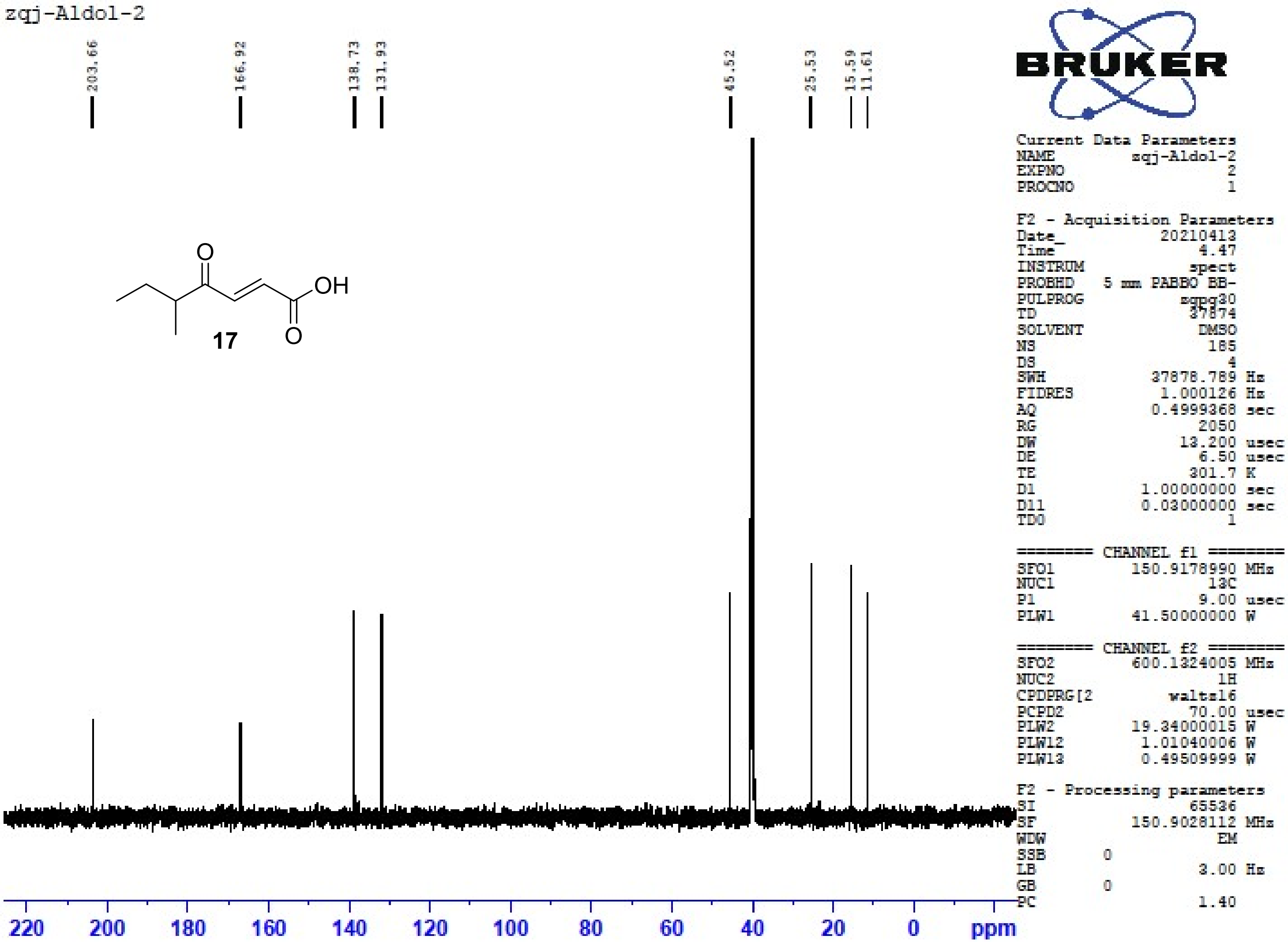
zqj-Aldol-2
203.66
166.92
138.73
131.93
45.52
25.53
15.59
11.61
OH
O
O
17
BRUKER
Current Data Parameters
NAME zqj-Aldol-2
EXPNO 2
PROCNO 1
F2 - Acquisition Parameters
Date_ 20210413
Time 4.47
INSTRUM spect
PROBHD 5 mm PABBO BB-
PULPROG zgpg30
TD 37874
SOLVENT DMSO
NS 185
DS 4
SWH 37878.789 Hz
FIDRES 1.000126 Hz
AQ 0.4999368 sec
RG 2050
DW 13.200 usec
DE 6.50 usec
TE 301.7 K
D1 1.00000000 sec
D11 0.03000000 sec
TD0 1
======== CHANNEL f1 ========
SFO1 150.9178990 MHz
NUC1 13C
P1 9.00 usec
PLW1 41.50000000 W
======== CHANNEL f2 ========
SFO2 600.1324005 MHz
NUC2 1H
CPDPRG[2 waltz16
PCPD2 70.00 usec
PLW2 19.34000015 W
PLW12 1.01040006 W
PLW13 0.49509999 W
F2 - Processing parameters
SI 65536
SF 150.9028112 MHz
WDW EM
SSB 0
LB 3.00 Hz
GB 0
PC 1.40
220
200
180
160
140
120
100
80
60
40
20
0
ppm

Compound 19

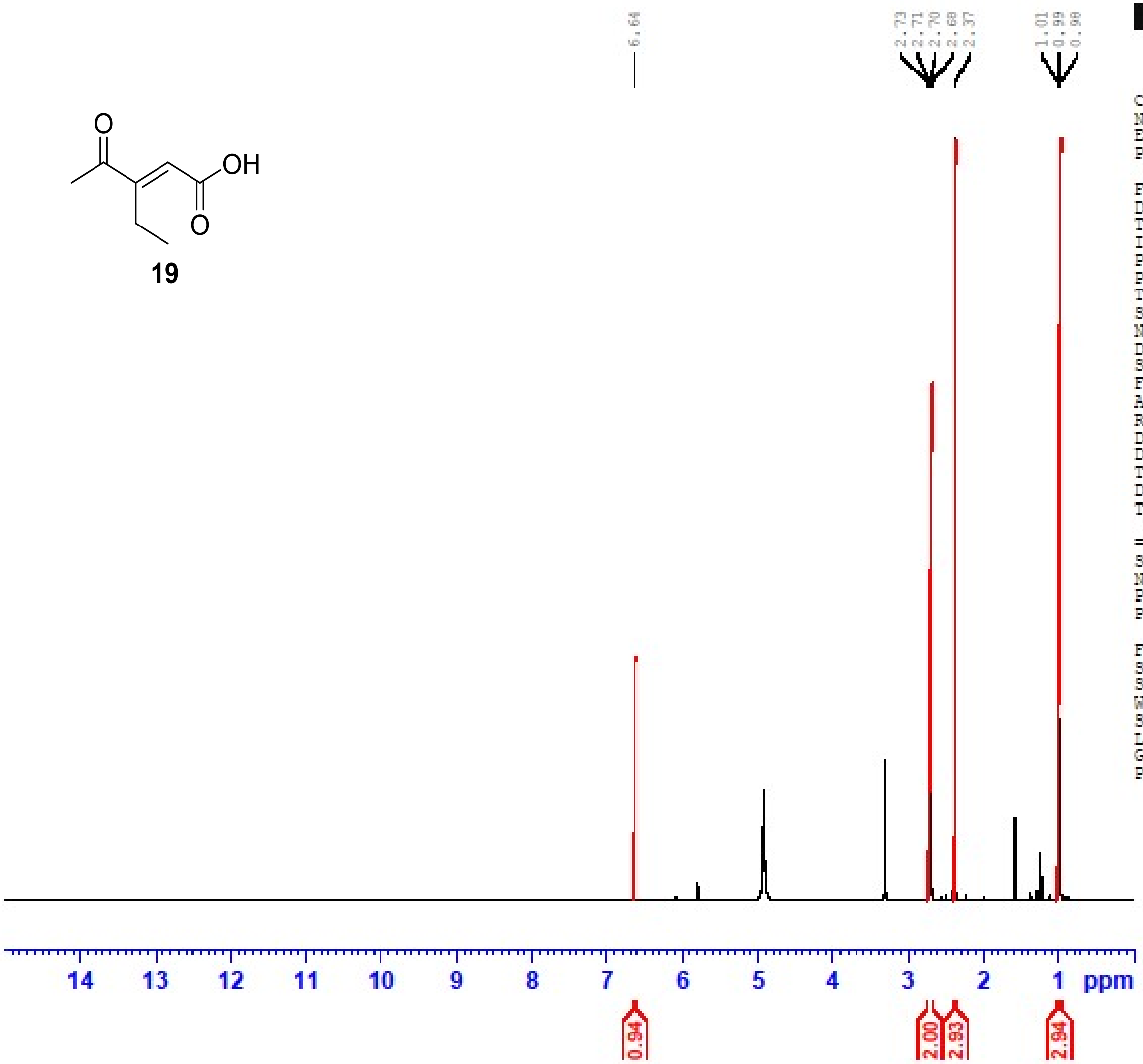

Current Data Parameters
NAME MU-666-121-F2-A
EXPNO 20
PROCNO 1

F2 - Acquisition Parameters
Date_ 20200214
Time 1.31
INSTRUM spect
PROBHD 5 mm PABBO BB-
PULPROG zg30
TD 65536
SOLVENT MeOD
NS 16
DS 0
SWH 10330.578 Hz
FIDRES 0.157632 Hz
AQ 3.1719425 sec
RG 287
DW 48.400 usec
DE 12.02 usec
TE 292.6 K
D1 1.00000000 sec
TD0 1

======== CHANNEL f1 ========
SFO1 500.3030896 MHz
NUC1 1H
P1 16.10 usec
PLW1 19.50000000 W

F2 - Processing parameters
SI 65536
SF 500.3000000 MHz
WDW EM
SSB 0
LB 0.30 Hz
GB 0
PC 1.00

Compound 19

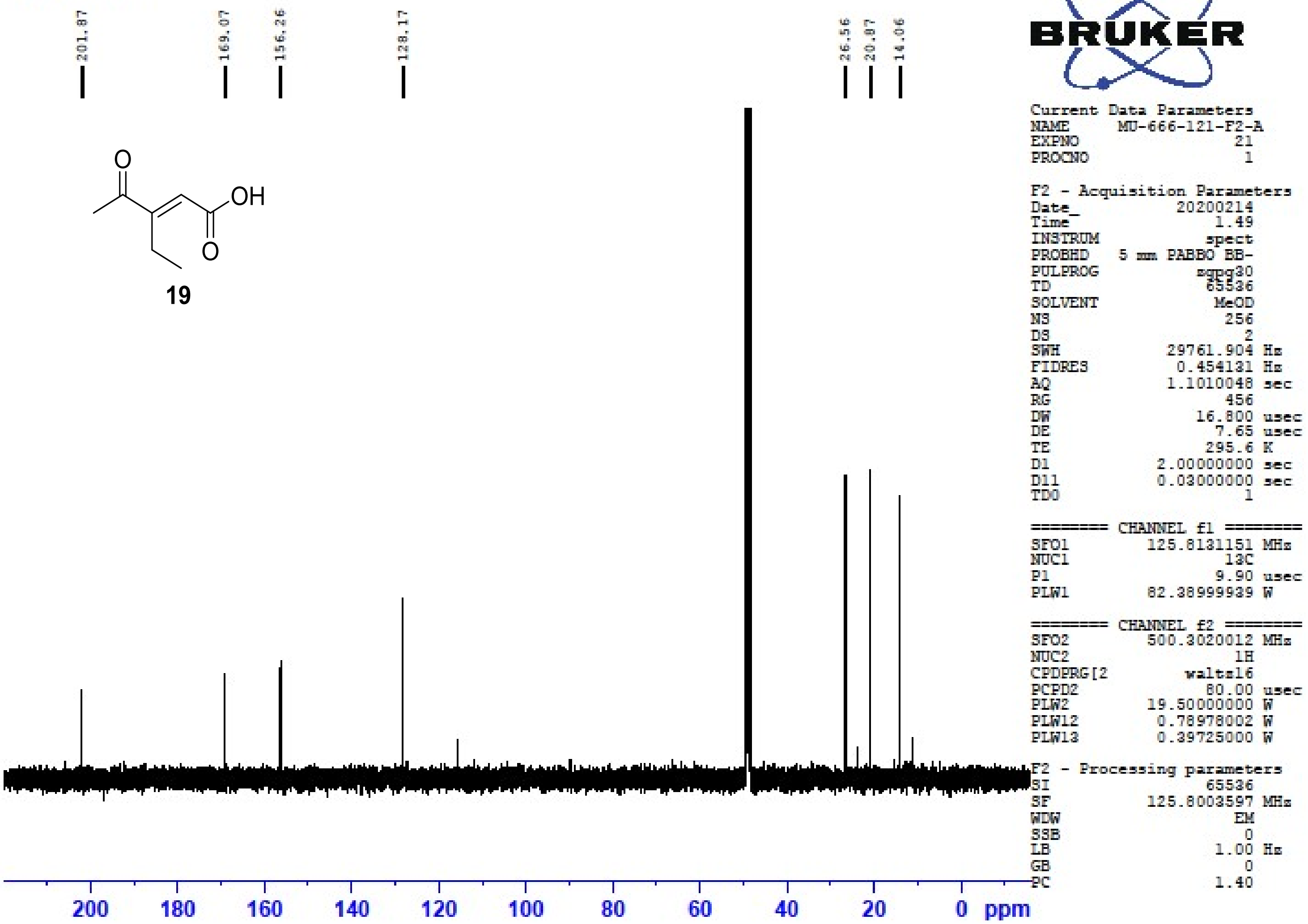

201.87
169.07
156.26
128.17
26.56
20.87
14.06
O
OH
O
19
BRUKER
Current Data Parameters
NAME MU-666-121-F2-A
EXPNO 21
PROCNO 1
F2 - Acquisition Parameters
Date_ 20200214
Time 1.49
INSTRUM spect
PROBHD 5 mm PABBO BB-
PULPROG zgpg30
TD 65536
SOLVENT MeOD
NS 256
DS 2
SWH 29761.904 Hz
FIDRES 0.454131 Hz
AQ 1.1010048 sec
RG 456
DW 16.800 usec
DE 7.65 usec
TE 295.6 K
D1 2.00000000 sec
D11 0.03000000 sec
TD0 1
======== CHANNEL f1 ========
SFO1 125.8131151 MHz
NUC1 13C
P1 9.90 usec
PLW1 82.38999939 W
======== CHANNEL f2 ========
SFO2 500.3020012 MHz
NUC2 1H
CPDPRG[2 waltz16
PCPD2 80.00 usec
PLW2 19.50000000 W
PLW12 0.78978002 W
PLW13 0.39725000 W
F2 - Processing parameters
SI 65536
SF 125.8003597 MHz
WDW EM
SSB 0
LB 1.00 Hz
GB 0
PC 1.40
200 180 160 140 120 100 80 60 40 20 0 ppm
